# Supplementary material for: Male sex determination maintains proteostasis and extends lifespan of daf-18/PTEN deficient C. elegans
Source: EMBO Rep. 2025 Jan 16;26(4):1084–113. doi: 10.1038/s44319-025-00368-x (PMC11850635; doi:10.1038/s44319-025-00368-x)
Supplement: Supplementary file 1 — Appendix [file 44319_2025_368_MOESM1_ESM.pdf]

Appendix

Male sex determination maintains proteostasis and extends lifespan of *daf-18/PTEN* deficient *C. elegans*

Zhi Qu [1,2,5], Lu Zhang[3,5], Xue Yin[3,5], Fangzhou Dai[3], Wei Huang[3], Yutong, Zhang[3], Dongyang Ran[3], Shanqing Zheng [1,3,4*]

[1]The Zhongzhou Laboratory for Integrative Biology, Zhengzhou, Henan, 450000, China

[2] School of Nursing and Health, Henan University, Kaifeng 475004, China

[3] School of Basic Medical Sciences, Henan University, Kaifeng 475004, China

[4] Laboratory of Cell Signal Transduction, Henan Provincial Engineering Centre for Tumor Molecular Medicine, Medical School of Henan University, Kaifeng 475004, China

[5]: These authors contributed equally: Zhi Qu, Lu Zhang, Xue Yin

*: Corresponding author: Shanqing Zheng. Email: zhengshanqing@henu.edu.cn

ORCID: 0000-0002-5039-0196

List of Appendix Figures and Tables

Appendix Figures	
**Appendix Figure S1. Survival experiments repeated using worms without *him-5(e1490)*.**	**Page 3**
**Appendix Figure S2. Lifespan of adult males and hermaphrodites tested using different alleles of *daf-18*.**	**Page 4**
**Appendix Figure S3. Survival experiments were repeated by culturing the adult *daf-18(ok480);him-5(e1490)* worms individually.**	**Page 5**
**Appendix Figure S4. The expression of ER-related protein processing genes in wild-type males was compared with that in hermaphrodites.**	**Page 6**
**Appendix Figure S5. Survival experiments were repeated by culturing the *unc-23* knockdown and overexpression adult *daf-18(ok480)* worms individually.**	**Page 7**
**Appendix Figure S6. UPR in mitochondrial and cytosol is not affected by DAF-18 protein phosphatase activity.**	**Page 8-9**
**Appendix Figure S7. The GO analysis of expression of changed genes in *daf-18(ok480)* males.**	**Page 10-12**
**Appendix Figure S8. The expression of male sex determination genes in *daf-18(ok480)* worms.**	**Page 13**
**Appendix Figure S9. The adult lifespan experiments of *daf-18(ok480)* worms, affected by sex determination genes, were repeated using worms without *him-5(e1490)*.**	**Page 14**
**Appendix Figure S10. Survival experiments were repeated by individually culturing *daf-18(ok480)* worms treated with *tra-3* and *fem-2* RNAi.**	**Page 15**
**Appendix Figure S11. Male sex determination gene upregulated in *C18E9.2* males.**	**Page 16**
**Appendix Tables**	
**Appendix Table S1. Lifespan experiments repeated using worms without *him-5* mutation.**	**Page 17-18**
**Appendix Table S2. Lifespan of *daf-18* worms repeated using different alleles.**	**Page 19-20**
**Appendix Table S3. Lifespan experiments of worms cultured individually.**	**Page 21-22**
**Appendix Table S4. The lifespan of worms.**	**Page 23-25**
**Appendix Table S5. The results of western blot.**	**Page 26**
**Appendix Table S6. Primers for real- time PCR.**	**Page 27**

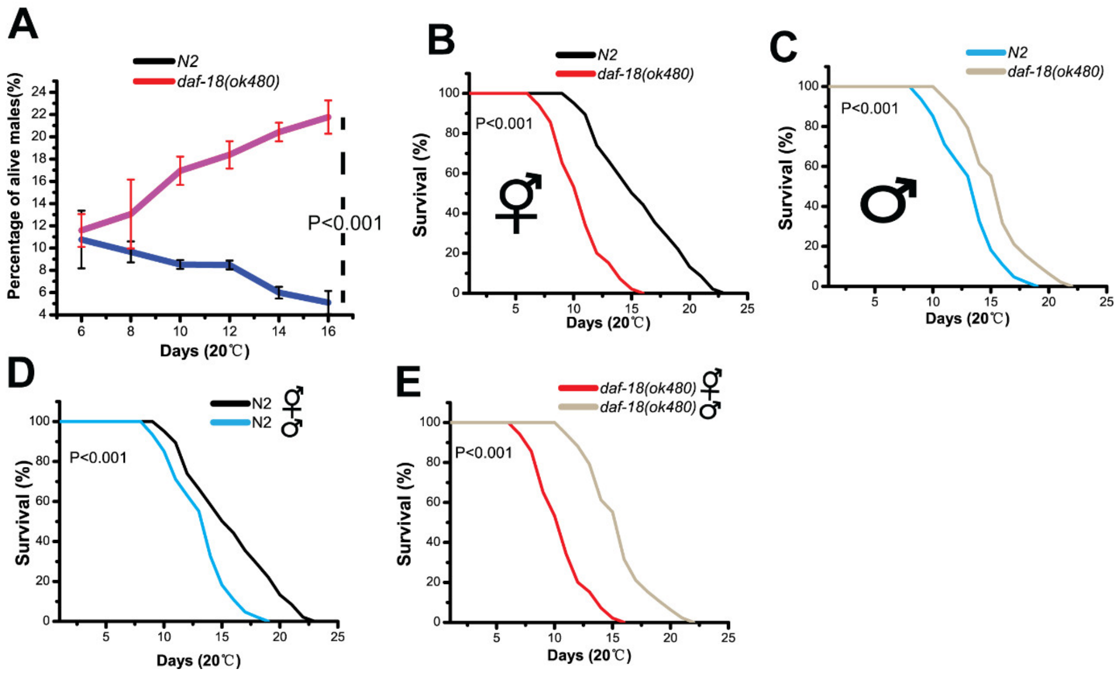

Appendix Figure S1. Survival experiments repeated using worms without *him-5(e1490)*.

(**A**) Percentages of live males recovered from L1 arrest each day. The data show the average of three independent repeats, and the error bars show the standard deviations. *P* values were determined by using a two-tailed t *test*. Survival curves of adult hermaphrodites (**B**) and males (**C**). Difference in survival between adult hermaphrodites and males of N2 (**D**) and *daf-18(ok480)* (**E**) worms. (**B-E**) *P* values were determined by using the log rank test. Control for RNAi: Feeding RNAi control clones with empty vector L4440. Each survival curve is representative of three independent repeats; please see the detailed data in Appendix Table S1.

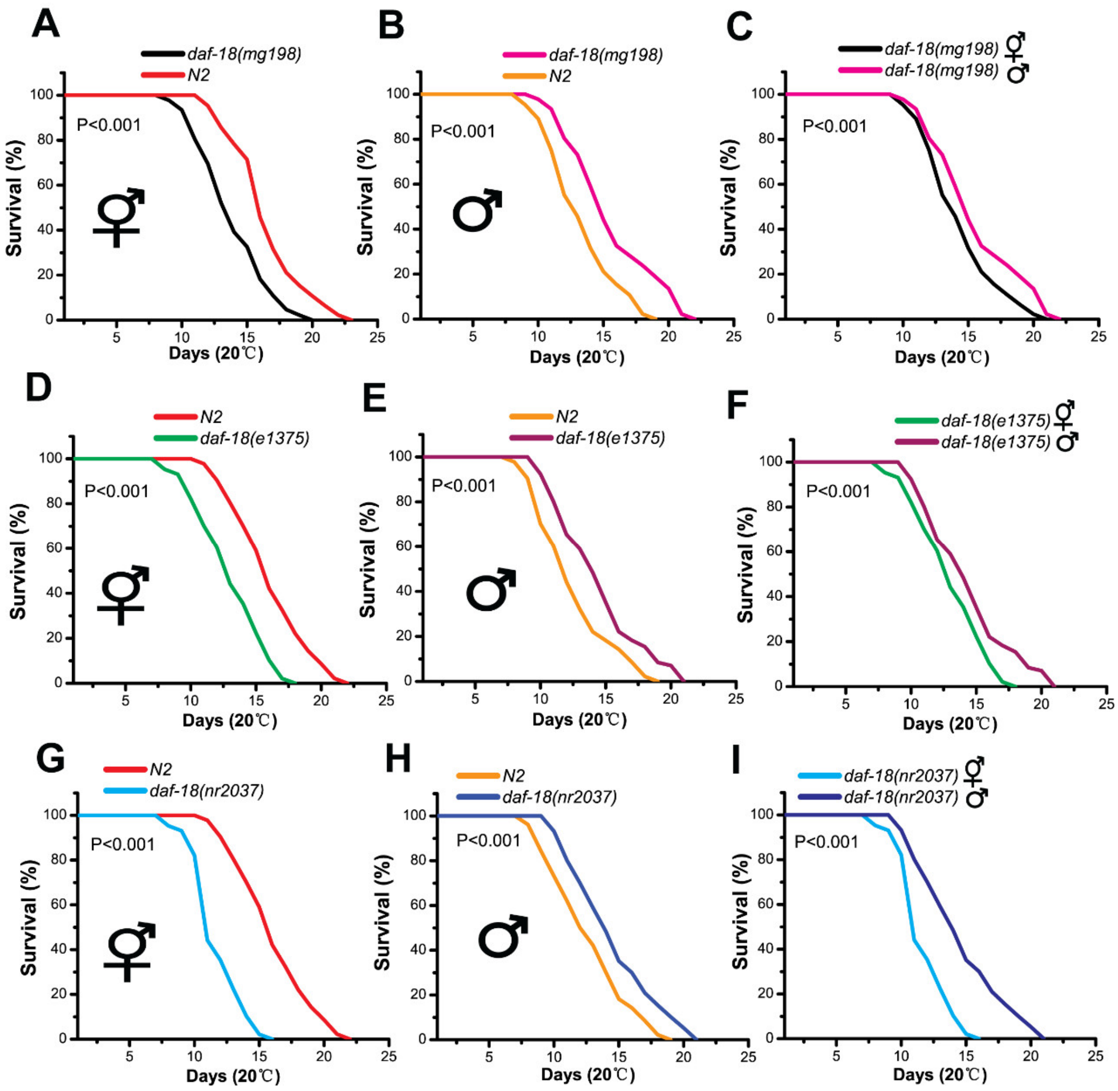

Appendix Figure S2. Lifespan of adult males and hermaphrodites tested using different alleles of *daf-18*.

(**A-C**) Survival curves of adult *daf-18 (mg198)* hermaphrodites and males. (**D-F**) Survival curves of adult *daf-18 (e1375)* hermaphrodites and males. (**G-I**) Survival curves of adult *daf-18 (nr2037)* hermaphrodites and males. Each survival curve is representative of three independent repeats. The mean survival rates were calculated using the Kaplan-Meier method, and *P* values were carried out by using log rank test. All the detailed lifespan data were summarized in Appendix Table S2.

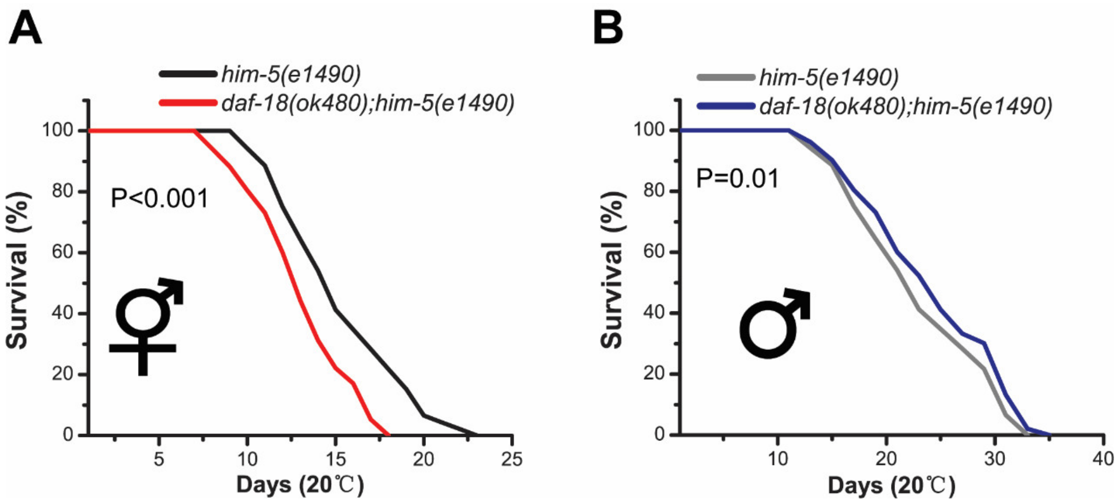

Appendix Figure S3. Survival experiments were repeated by culturing the adult *daf-18(ok480);him-5(e1490)* worms individually.

Survival curves of adult *daf-18(ok480);him-5(e1490)* hermaphrodites (**A**) and males (**B**). Each survival curve is representative of three independent repeats. The mean survival rates were calculated using the Kaplan-Meier method, and *P* values were carried out by using log rank test. All the detailed lifespan data were summarized in Appendix Table S3.

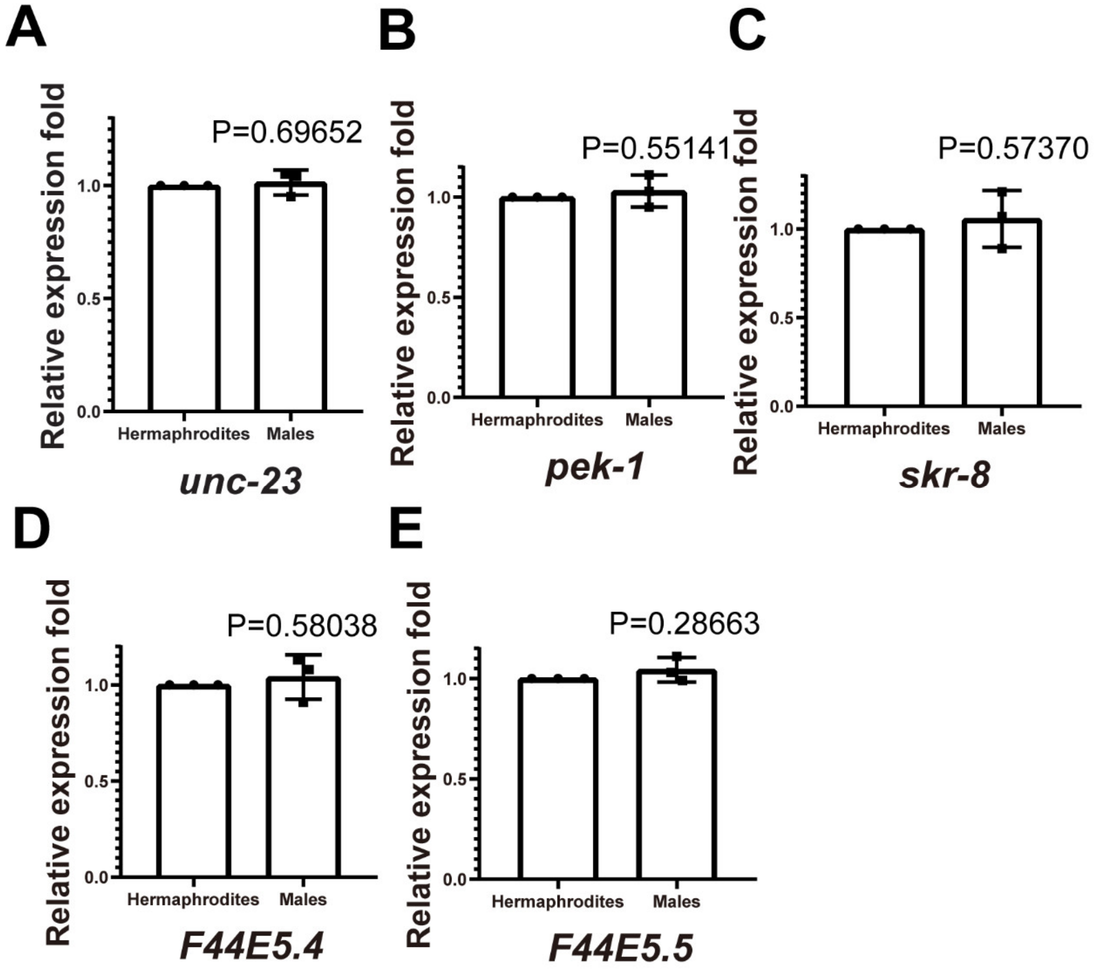

Appendix Figure S4. The expression of ER-related protein processing genes in wild-type males was compared with that in hermaphrodites. Real time PCR results of *unc-23* (**A**), *pek-1*(**B**), *skr-8* (**C**), *F44E5.4* (**D**) and *F44E5.5* (**E**) between two sexes. The experiment was repeated three times independently. The data show the average of three independent repeats, and the error bars show the standard deviations. P value was analyzed by two-tailed *t-test*.

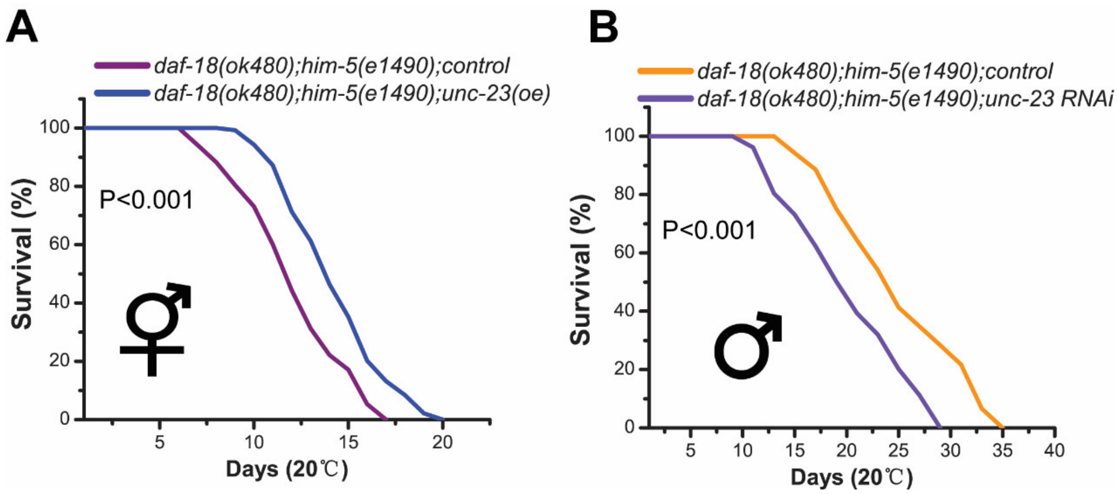

Appendix Figure S5. Survival experiments were repeated by culturing the *unc-23* knockdown and overexpression adult *daf-18(ok480)* worms individually. (**A**) Overexpression of *unc-23* improved the survival of adult *daf-18(ok480)* hermaphrodites. Control: transgenic injection strains with the empty expression vector L2528. (**B**) Knocking down *unc-23* significantly reduced the lifespan of adult *daf-18(ok480)* males. Control: RNAi control clones containing the empty vector L4440. Each survival curve is representative of three independent repeats. The mean survival rates were calculated using the Kaplan–Meier method, and *P* values were determined by using the log rank test. All the detailed lifespan data are summarized in Appendix Table S3.

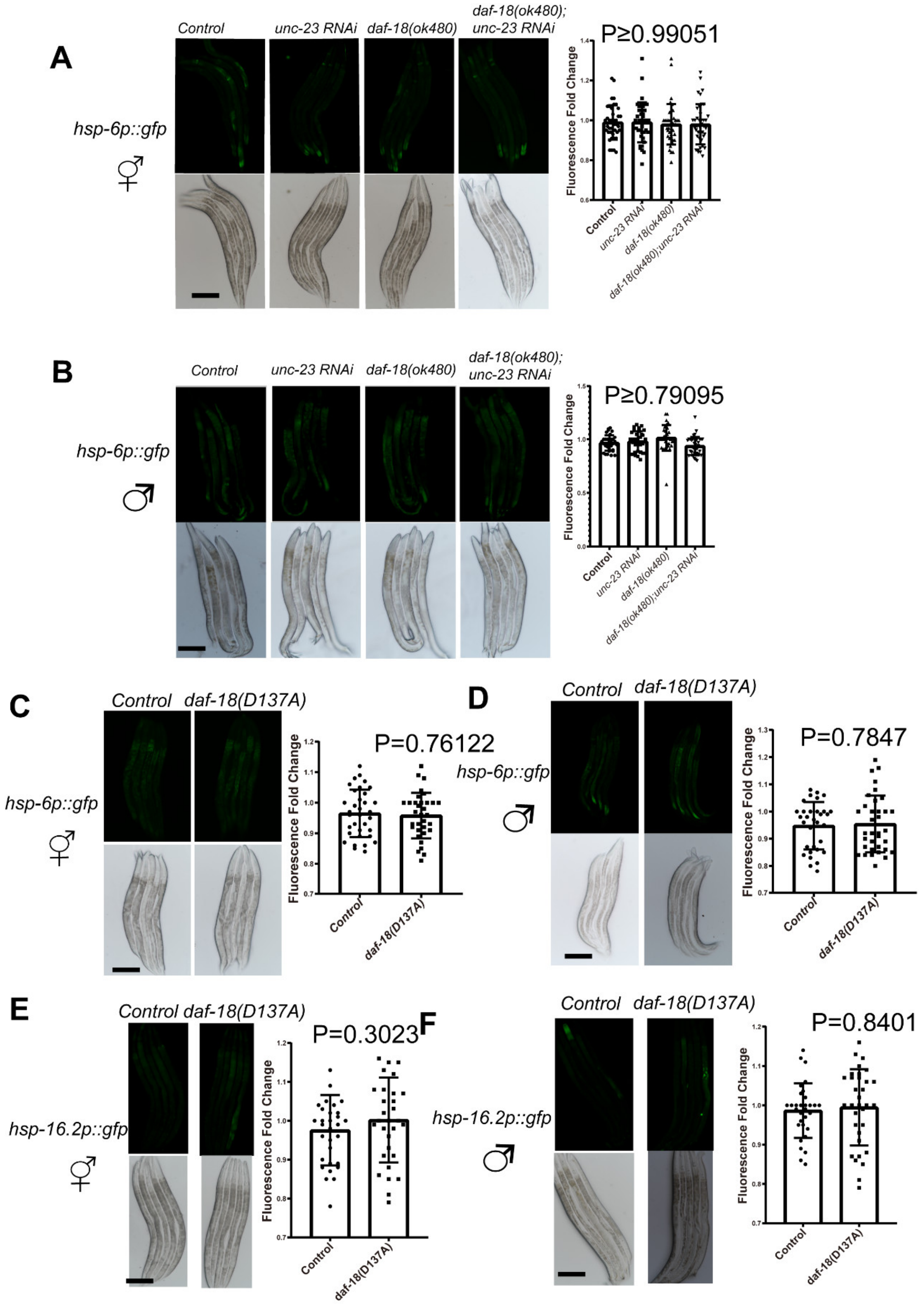

Appendix Figure S6. UPR in mitochondrial and cytosol is not affected by DAF-18 protein phosphatase activity.

The mitochondrial UPR is tested by using *hsp-6p::gfp* in hermaphrodites (**A**) and males (**B**). DAF-18 protein phosphatase deficient did not affect the mitochondrial UPR in hermaphrodites (**C**) and males (**D**). DAF-18 protein phosphatase deficient did not affect the cytosol UPR in hermaphrodites (**E**) and males (**F**). Each experimental set had three independent repeats, and the sample size was 30. The data shows the values of all samples from three replicates, with error bars representing the averages and standard deviations. *P* values were determined by using two-tailed *t-test*. Scale bar: 200 μm.

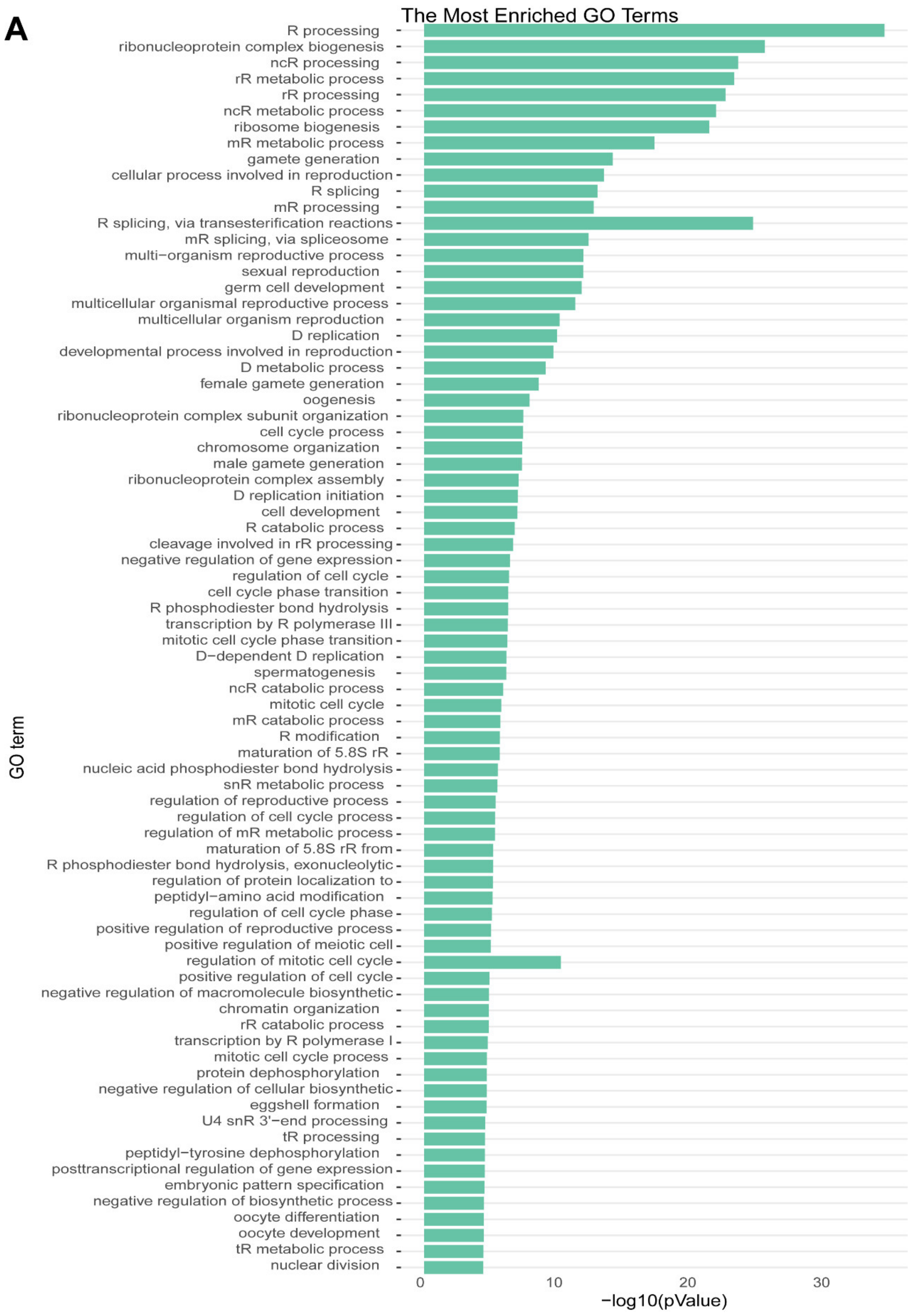
A
The Most Enriched GO Terms
R processing
ribonucleoprotein complex biogenesis
ncR processing
rR metabolic process
rR processing
ncR metabolic process
ribosome biogenesis
mR metabolic process
gamete generation
cellular process involved in reproduction
R splicing
mR processing
R splicing, via transesterification reactions
mR splicing, via spliceosome
multi−organism reproductive process
sexual reproduction
germ cell development
multicellular organismal reproductive process
multicellular organism reproduction
D replication
developmental process involved in reproduction
D metabolic process
female gamete generation
oogenesis
ribonucleoprotein complex subunit organization
cell cycle process
chromosome organization
male gamete generation
ribonucleoprotein complex assembly
D replication initiation
cell development
R catabolic process
cleavage involved in rR processing
negative regulation of gene expression
regulation of cell cycle
cell cycle phase transition
R phosphodiester bond hydrolysis
transcription by R polymerase III
mitotic cell cycle phase transition
D−dependent D replication
spermatogenesis
ncR catabolic process
mitotic cell cycle
mR catabolic process
R modification
maturation of 5.8S rR
nucleic acid phosphodiester bond hydrolysis
snR metabolic process
regulation of reproductive process
regulation of cell cycle process
regulation of mR metabolic process
maturation of 5.8S rR from
R phosphodiester bond hydrolysis, exonucleolytic
regulation of protein localization to
peptidyl−amino acid modification
regulation of cell cycle phase
positive regulation of reproductive process
positive regulation of meiotic cell
regulation of mitotic cell cycle
positive regulation of cell cycle
negative regulation of macromolecule biosynthetic
chromatin organization
rR catabolic process
transcription by R polymerase I
mitotic cell cycle process
protein dephosphorylation
negative regulation of cellular biosynthetic
eggshell formation
U4 snR 3'−end processing
tR processing
peptidyl−tyrosine dephosphorylation
posttranscriptional regulation of gene expression
embryonic pattern specification
negative regulation of biosynthetic process
oocyte differentiation
oocyte development
tR metabolic process
nuclear division
GO term
0
10
20
30
−log10(pValue)

B

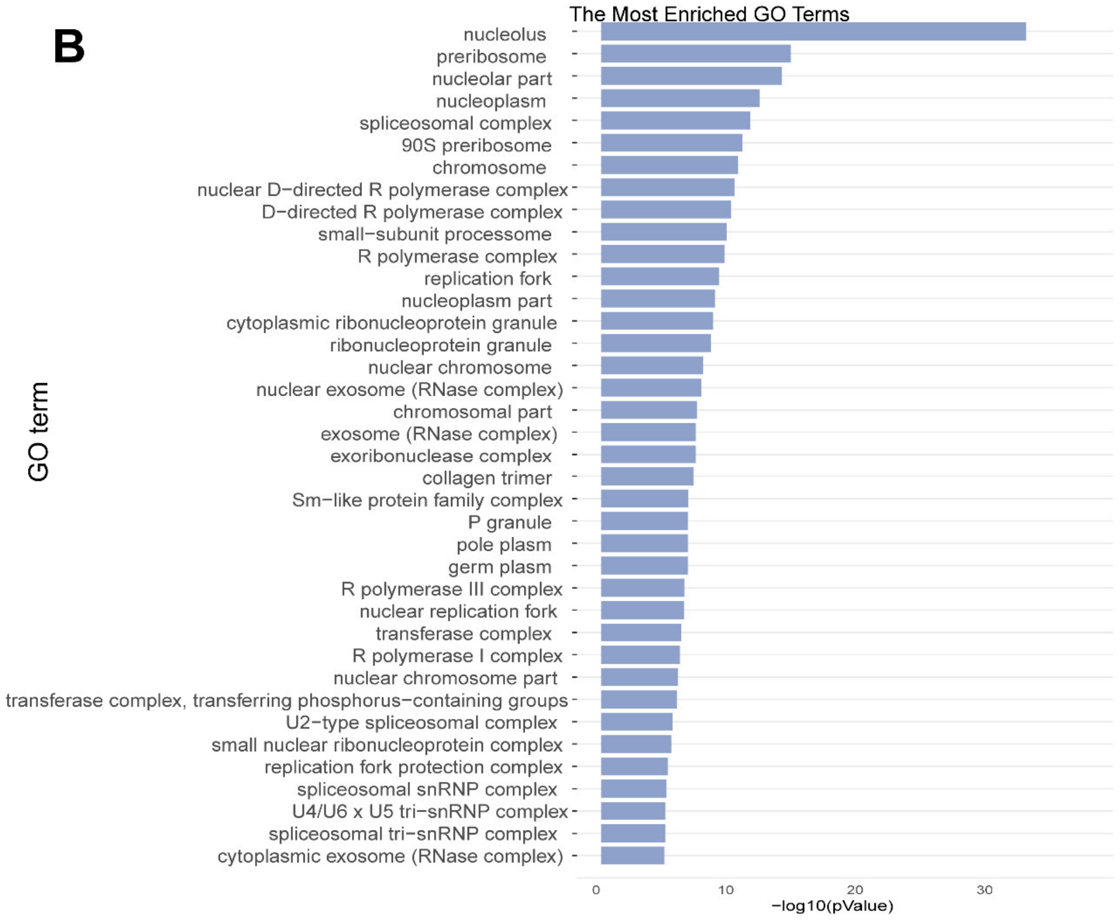
The Most Enriched GO Terms
GO term
nucleolus
preribosome
nucleolar part
nucleoplasm
spliceosomal complex
90S preribosome
chromosome
nuclear D−directed R polymerase complex
D−directed R polymerase complex
small−subunit processome
R polymerase complex
replication fork
nucleoplasm part
cytoplasmic ribonucleoprotein granule
ribonucleoprotein granule
nuclear chromosome
nuclear exosome (RNase complex)
chromosomal part
exosome (RNase complex)
exoribonuclease complex
collagen trimer
Sm−like protein family complex
P granule
pole plasm
germ plasm
R polymerase III complex
nuclear replication fork
transferase complex
R polymerase I complex
nuclear chromosome part
transferase complex, transferring phosphorus−containing groups
U2−type spliceosomal complex
small nuclear ribonucleoprotein complex
replication fork protection complex
spliceosomal snRNP complex
U4/U6 x U5 tri−snRNP complex
spliceosomal tri−snRNP complex
cytoplasmic exosome (RNase complex)
0
10
20
30
−log10(pValue)

C

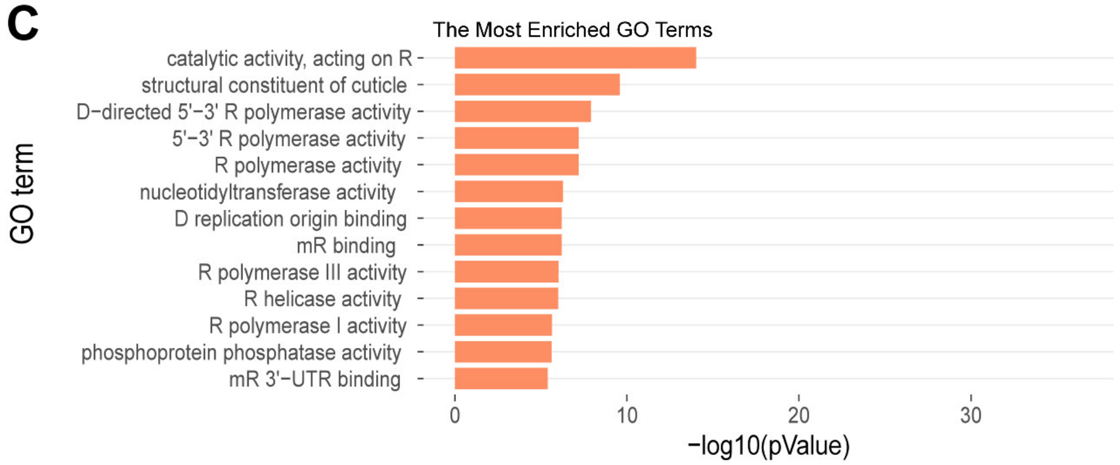
The Most Enriched GO Terms
GO term
catalytic activity, acting on R
structural constituent of cuticle
D−directed 5'−3' R polymerase activity
5'−3' R polymerase activity
R polymerase activity
nucleotidyltransferase activity
D replication origin binding
mR binding
R polymerase III activity
R helicase activity
R polymerase I activity
phosphoprotein phosphatase activity
mR 3'−UTR binding
0
10
20
30
−log10(pValue)

D

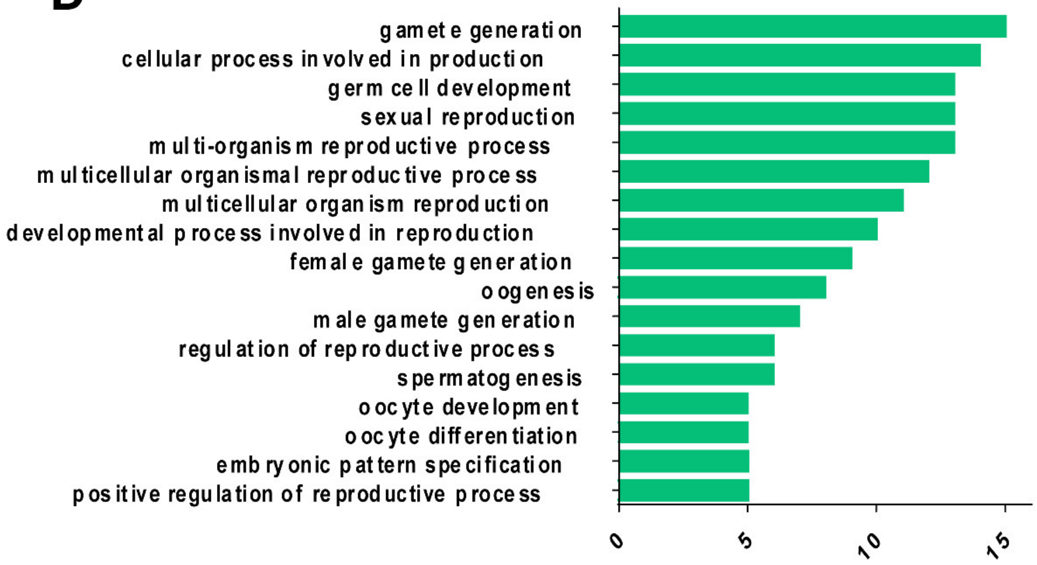

Appendix Figure S7. The GO analysis of expression of changed genes in *daf-18(ok480)* males.

The up- and down-regulated genes more than 2X were used to generate the GO results. (**A**) GO term: Biological process. (**B**) GO term: Cellular component. (**C**) GO term: Molecular function. (**D**) Most changed genes are involved sex determination and sex development phenotypes.

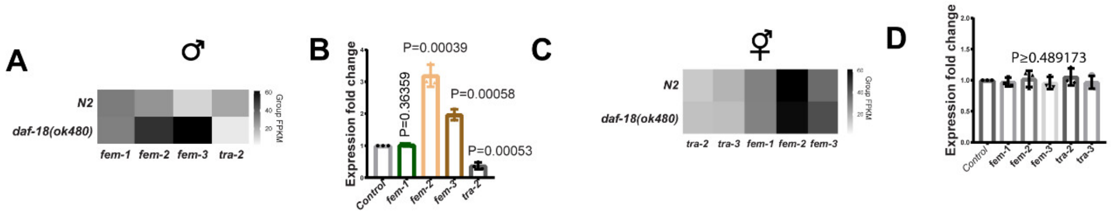

Appendix Figure S8. The expression of male sex determination genes in *daf-18(ok480)* worms.

(**A**) The average expression of the male sex determination genes *fem-2/3* and *tra-2* was altered in *daf-18(ok480)* males according to RNA-seq data. Group FPKM: average of the log-scaled FPKM: log2(FPKM+1) of the gene in groups. (**B**) Real-time PCR confirmed the expression changes in males. The data show the average of three independent repeats, and the error bars show the standard deviations. P value was analyzed by two-tailed *t-test*. (**C**) The average expression of the male sex determination genes *fem-1/2/3* and *tra-2/3* was not changed in *daf-18(ok480)* hermaphrodites according to RNA-seq data. Group FPKM: average of the log-scaled FPKM: log2(FPKM+1) of the gene in groups. (**D**) Real-time PCR confirmed the expression changes in hermaphrodites. The experiment was repeated three times independently. The data show the average of three independent repeats, and the error bars show the standard deviations. P value was analyzed by two-tailed *t-test*.

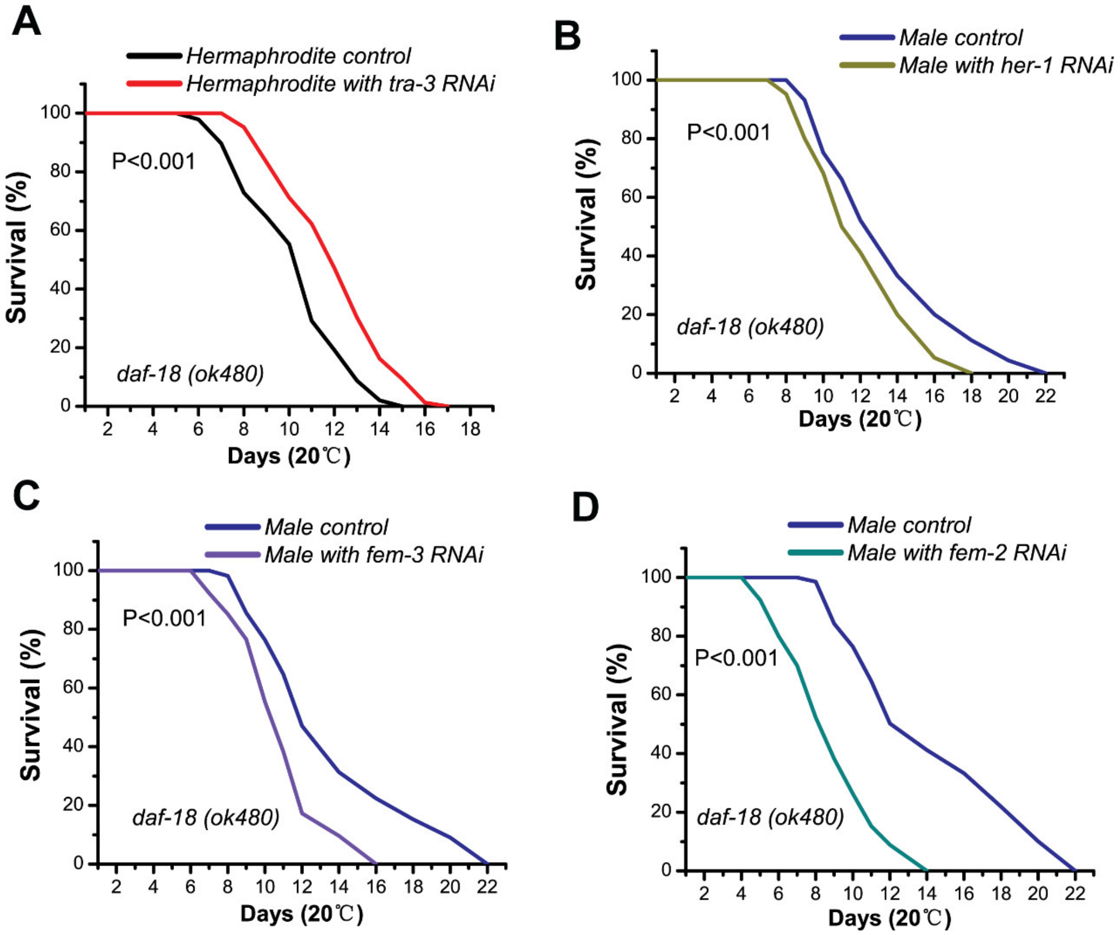

Appendix Figure S9. The adult lifespan experiments of *daf-18(ok480)* worms, affected by sex determination genes, were repeated using worms without *him-5(e1490)*. (**A**) Enhancing male sex determination signaling by knocking down *tra-3* extended lifespan of *daf-18(ok480)* hermaphrodites. Reducing male sex determination signaling by knocking down *her-1* (**B**), *fem-3* (**C**) or *fem-2* (**D**) decreased lifespan of *daf-18(ok480)* males. Control for RNAi: Feeding RNAi control clones with empty vector L4440. Each survival curve is representative of three independent repeats. The mean survival rates were calculated using the Kaplan–Meier method, and *P* values were determined by using the log rank test. All the detailed lifespan data are summarized in Appendix Table S1.

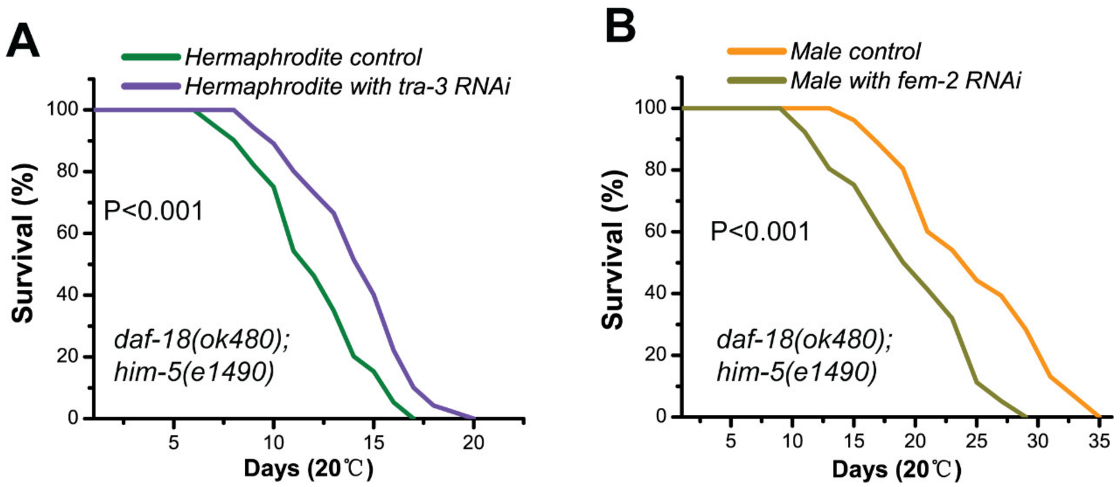

Appendix Figure S10. Survival experiments were repeated by individually culturing *daf-18(ok480)* worms treated with *tra-3* and *fem-2* RNAi.

(**A**) Enhancing male sex determination signaling by knocking down *tra-3* extended adult lifespan of *daf-18(ok480)* hermaphrodites. (**B**) Reducing male sex determination signaling by knocking down *fem-2* decreased adult lifespan of *daf-18(ok480)* males. Control for RNAi: Feeding RNAi control clones with empty vector L4440. Each survival curve is representative of three independent repeats. The mean survival rates were calculated using the Kaplan–Meier method, and *P* values were determined by using the log rank test. All the detailed lifespan data are summarized in Appendix Table S3.

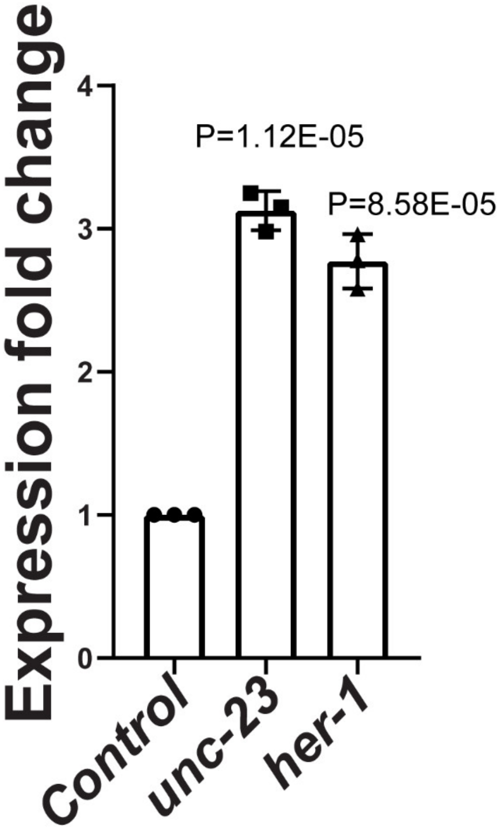

Appendix Figure S11. Male sex determination gene upregulated in *C18E9.2* males. *unc-23* and male sex determination gene *her-1* were upregulated in *daf-18(ok480); C18E9.2 (S301A)* worms. The experiment was repeated three times independently. The data show the average of three independent repeats, and the error bars show the standard deviations. P value was analyzed by two-tailed *t-test*.

Appendix Table S1. Lifespan experiments repeated using worms without *him-5* mutation.

Strains		Repeats	mean lifespan (days)	Maximum lifespan(days)	*P* value	N	
N2	Hermaphrodite	Exp 1	15.76±0.25	23		101	Appendix Figure S1B
		Exp 2	15.69±0.33	23		93	
		Exp 3	15.38±0.13	23		88	
daf-18(ok480)		Exp 1	10.95±0.36	16	0.00029	61	
		Exp 2	11.01±0.22	16	0.00087	66	
		Exp 3	10.54±0.37	16	0.00099	97	
N2	Male	Exp 1	12.83±0.25	19		75	Appendix Figure S1C, D
		Exp 2	12.96±0.21	20		77	
		Exp 3	12.62±0.19	19		107	
daf-18(ok480)		Exp 1	15.23±0.14	23	0.00082	99	
		Exp 2	15.36±0.11	22	0.00017	90	
		Exp 3	15.74±0.92	22	0.00037	105	
daf-18(ok480)	Hermaphrodite	Exp 1	10.95±0.36	16		61	Appendix Figure S1E
		Exp 2	11.01±0.22	16		66	
		Exp 3	10.54±0.37	16		97	
daf-18(ok480)	Male	Exp 1	15.23±0.14	23	0.00082	99	
		Exp 2	15.36±0.11	22	0.00012	90	
		Exp 3	15.74±0.92	22	0.00043	105	
daf-18(ok480); control	Hermaphrodite	Exp 1	10.58±0.26	15		77	Appendix Figure S9A
		Exp 2	10.44±0.18	15		63	
		Exp 3	10.56±0.21	15		60	
daf-18(ok480) tra-3 RNAi	Hermaphrodite	Exp 1	12.56±0.21	17	0.00068	81	
		Exp 2	12.64±0.11	17	0.00053	85	
		Exp 3	12.73±0.44	17	0.00065	101	
daf-18(ok480); control	Male	Exp 1	13.26±0.33	20		70	Appendix Figure S9B
		Exp 2	13.78±0.19	21		98	
		Exp 3	13.45±0.08	22		118	
daf-18(ok480) her-1 RNAi	Male	Exp 1	11.02±0.08	15	0.00041	125	
		Exp 2	10.92±0.15	15	0.00057	109	
		Exp 3	10.84±0.46	15	0.00065	68	
daf-18(ok480); control	Male	Exp 1	12.68±0.18	20		99	Appendix Figure S9C
		Exp 2	13.05±0.14	21		64	
		Exp 3	13.14±0.17	22		118	
daf-18(ok480) fem-3 RNAi	Male	Exp 1	10.35±0.69	14	0.00085	107	
		Exp 2	10.68±0.15	14	0.00055	112	
		Exp 3	10.77±0.09	14	0.00099	93	
daf-18(ok480);	Male	Exp 1	13.05±0.55	21		75	Appendix Figure

control		Exp 2	13.14±0.47	22		77	S9D
		Exp 3	12.98±0.16	20		92	
daf-18(ok480) fem-2 RNAi	Male	Exp 1	8.89±0.36	13	0.00098	114	
		Exp 2	9.04±0.11	13	0.00099	118	
		Exp 3	8.75±0.34	13	0.00036	107	

Control for RNAi: Feeding RNAi control clones with empty vector L4440. Mean lifespans were calculated by using Kaplan-Meier method; *P* values were determined using the log-rank test; SEM: standard error of the mean; N: number of worms used.

Appendix Table S2. Lifespan of *daf-18* worms repeated using different alleles.

Strains		Repeats	mean lifespan (days)	Maximum lifespan(days)	*P* value	N	
N2	Hermaphrodite	Exp 1	16.01±0.22	23		111	Appendix Figure S2A
		Exp 2	15.34±018	22		96	
		Exp 3	15.22±0.07	22		80	
daf-18(mg198)		Exp 1	12.35±0.09	20	0.00015	79	
		Exp 2	13.05±0.85	20	0.00044	104	
		Exp 3	12.28±0.36	20	0.00085	95	
N2	Male	Exp 1	13.23±0.11	20		115	Appendix Figure S2B
		Exp 2	12.58±0.37	19		94	
		Exp 3	12.63±0.14	19		92	
daf-18(mg198)		Exp 1	15.01±0.12	23	0.00036	88	
		Exp 2	14.96±0.33	22	0.00059	76	
		Exp 3	14.85±0.24	22	0.00077	68	
daf-18(mg198)	Hermaphrodite	Exp 1	12.35±0.09	20		79	Appendix Figure S2C
		Exp 2	13.05±0.85	20		104	
		Exp 3	12.28±0.36	20		95	
	Male	Exp 1	15.01±0.12	23	0.00065	88	
		Exp 2	14.96±0.33	22	0.00027	76	
		Exp 3	14.85±0.24	22	0.00099	68	
N2	Hermaphrodite	Exp 1	16.01±0.22	23		111	Appendix Figure S2D
		Exp 2	15.34±018	22		96	
		Exp 3	15.22±0.07	22		80	
daf-18(e1375)		Exp 1	12.53±0.22	18	0.00061	114	
		Exp 2	12.64±0.15	18	0.00019	162	
		Exp 3	12.59±0.34	18	0.00074	84	
N2	Male	Exp 1	13.23±0.11	20		115	Appendix Figure S2E
		Exp 2	12.58±0.37	19		94	
		Exp 3	12.63±0.14	19		92	
daf-18(e1375)		Exp 1	14.39±0.22	23	0.00087	90	
		Exp 2	14.83±0.16	22	0.00034	110	
		Exp 3	14.55±0.08	22	0.00013	87	
daf-18(e1375)	Hermaphrodite	Exp 1	12.53±0.22	18		114	Appendix Figure S2F
		Exp 2	12.64±0.15	18		162	
		Exp 3	12.59±0.34	18		84	
	Male	Exp 1	14.39±0.22	23	0.00094	90	
		Exp 2	14.83±0.16	22	0.00084	110	
		Exp 3	14.55±0.08	22	0.00063	87	
N2	Hermaphrodite	Exp 1	16.01±0.22	23		111	Appendix Figure S2G
		Exp 2	15.34±018	22		96	
		Exp 3	15.22±0.07	22		80	

daf-18 (nr2037)		Exp 1	10.59±0.25	18	0.00011	59	
		Exp 2	11.02±0.19	18	0.00073	61	
		Exp 3	10.36±0.52	18	0.00020	115	
N2	Male	Exp 1	13.23±0.11	20		74	Appendix Figure S2H
		Exp 2	12.58±0.37	19		95	
		Exp 3	12.63±0.14	19		83	
daf-18 (nr2037)		Exp 1	14.66±0.32	21	0.00057	120	
		Exp 2	14.57±0.11	22	0.00064	91	
		Exp 3	14.68±0.12	22	0.00067	88	
daf-18 (nr2037)	Hermaphrodite	Exp 1	10.59±0.25	18		59	Appendix Figure S2I
		Exp 2	11.02±0.19	18		61	
		Exp 3	10.36±0.52	18		115	
	Male	Exp 1	14.66±0.32	21	0.00015	120	
		Exp 2	14.57±0.11	22	0.00035	91	
		Exp 3	14.68±0.12	22	0.00029	88	

Mean lifespans were calculated by using Kaplan-Meier method; *P* values were determined using the log-rank test; SEM: standard error of the mean; N: number of worms used.

Appendix Table S3. Lifespan experiments of worms cultured individually.

Strains		Repeats	mean lifespan (days)	Maximum lifespan(days)	*P* value	N	
him-5 (e1490)	Hermaphrodite	Exp 1	15.32±0.15	23		68	Appendix Figure S3A
		Exp 2	14.98±0.12	23		66	
		Exp 3	15.33±0.22	23		62	
daf-18 (ok480); him-5 (e1490)		Exp 1	12.58±0.22	17	0.000361	53	
		Exp 2	12.63±0.13	17	0.000644	56	
		Exp 3	12.33±0.45	17	0.000454	65	
him-5 (e1490)	male	Exp 1	20.70±0.26	33		60	Appendix Figure S3B
		Exp 2	20.54±0.15	33		56	
		Exp 3	19.94±0.25	33		67	
daf-18 (ok480); him-5 (e1490)		Exp 1	21.36±0.91	35	0.01	73	
		Exp 2	22.25±0.16	35	0.0054	68	
		Exp 3	22.86±0.37	35	0.0075	55	
daf-18 (ok480); him-5 (e1490); control	Hermaphrodite	Exp 1	12.03±0.11	17		66	Appendix Figure S5A
		Exp 2	12.25±0.25	17		61	
		Exp 3	12.86±0.17	17		60	
daf-18 (ok480); him-5 (e1490); unc-23 (oe)		Exp 1	14.98±0.65	20	0.000172	55	
		Exp 2	15.04±0.12	20	0.000238	68	
		Exp 3	14.88±0.21	20	0.000777	70	
daf-18 (ok480); him-5 (e1490); control	male	Exp 1	24.59±0.22	35		72	Appendix Figure S5B
		Exp 2	25.01±0.45	35		66	
		Exp 3	23.93±0.61	35		60	
daf-18 (ok480); him-5 (e1490); unc-23 RNAi		Exp 1	20.78±0.19	29	0.000894	58	
		Exp 2	20.04±0.46	29	0.00037	63	
		Exp 3	20.96±0.33	29	0.00087	69	
daf-18 (ok480); him-5 (e1490); control	Hermaphrodite	Exp 1	11.95±0.23	17		66	Appendix Figure S10A
		Exp 2	12.01±0.44	17		60	
		Exp 3	12.33±0.16	17		55	
daf-18 (ok480); him-5 (e1490); tra-3 RNAi		Exp 1	15.26±0.12	20	0.00011	58	
		Exp 2	15.09±0.16	20	0.000648	62	
		Exp 3	15.73±0.39	20	0.000788	68	
daf-18 (ok480); him-5 (e1490); control	male	Exp 1	25.13±0.11	35		64	Appendix Figure S10B
		Exp 2	24.98±0.48	35		58	
		Exp 3	25.11±0.67	35		61	
daf-18 (ok480); him-5 (e1490); fem-2 RNAi		Exp 1	18.44±0.63	29	0.000471	70	
		Exp 2	18.63±0.19	29	0.000195	65	
		Exp 3	19.11±0.27	29	0.000513	66	

Control for RNAi: Feeding RNAi control clones with empty vector L4440. Control for overexpression: the transgenic injection strains with empty expression vector

L2528. Mean lifespans were calculated by using Kaplan-Meier method; *P* values were determined using the log-rank test; SEM: standard error of the mean; N: number of worms used.

Appendix Table S4. The lifespan of worms.

Strains				Mean life Span ±SEM（Days）	Maximum life Span(Days)	*P* value	N	
daf-18 (ok480); him-5 (e1490)	Hermaphrodite	L4440 RNAi	Exp 1	10.29±0.32	15		96	
			Exp 2	10.57±0.35	15		92	
			Exp 3	10.49±0.27	15		107	
		pek-1 RNAi	Exp 1	10.49±0.38	16		90	
			Exp 2	11.22±0.39	18	0.50747	102	
			Exp 3	10.59±0.97	18	0.88864	73	
		skr-8 RNAi	Exp 1	10.31±0.15	15	0.35825	76	
			Exp 2	10.48±0.77	15	0.62139	97	
			Exp 3	10.51±0.31	15	0.82383	85	
		F44E5.4 RNAi	Exp 1	10.27±0.14	15	0.95762	76	
			Exp 2	10.60±0.47	15	0.32171	72	
			Exp 3	10.55±0.24	15	0.24694	98	
		F44E5.5 RNAi	Exp 1	10.27±0.17	15	0.82018	101	
			Exp 2	10.55±0.78	15	0.47627	65	
			Exp 3	10.54±0.67	15	0.42014	78	
daf-18 (ok480); him-5 (e1490)	Male	L4440 RNAi	Exp 1	12.70±0.33	20		106	
			Exp 2	12.26±0.42	19		82	
			Exp 3	12.29±0.36	21		70	
		pek-1 RNAi	Exp 1	11.17±0.51	18	0.75897	82	
			Exp 2	12.41±0.45	18	0.25601	102	
			Exp 3	12.35±0.18	17	0.83002	75	
		skr-8 RNAi	Exp 1	12.67±0.58	17	0.79787	98	
			Exp 2	12.20±0.46	18	0.48129	68	
			Exp 3	12.33±0.47	17	0.70127	89	
		F44E5.4 RNAi	Exp 1	12.68±0.88	17	0.31844	121	
			Exp 2	12.31±0.24	18	0.51712	107	
			Exp 3	12.34±0.94	17	0.91142	87	
		F44E5.5 RNAi	Exp 1	12.58±0.77	17	0.24985	69	
			Exp 2	12.28±0.42	17	0.66783	73	
			Exp 3	12.35±0.78	17	0.63577	85	
daf-18 (ok480); him-5 (e1490)	Hermaphrodite	L4440 RNAi	Exp 1	10.29±0.32	15		96	
			Exp 2	10.57±0.35	15		92	
			Exp 3	10.49±0.27	15		107	
		hsp-70 RNAi	Exp 1	10.20±0.39	15	0.71943	100	
			Exp 2	11.01±0.40	16	0.41821	92	
			Exp 3	10.48±0.18	15	0.62956	97	
		hsp-110	Exp 1	11.44±0.51	15	0.61856	92	
			Exp 2	10.69±0.48	15	0.21965	108	

		RNAi	Exp 3	10.58±0.35	15	0.66345	74	
daf-18 (ok480); him-5 (e1490)	Male	L4440 RNAi	Exp 1	12.70±0.33	20		106	
			Exp 2	12.26±0.42	19		82	
			Exp 3	12.29±0.36	21		70	
		hsp-70 RNAi	Exp 1	12.28±0.33	17	0.32294	114	
			Exp 2	12.07±0.42	17	0.67097	90	
			Exp 3	12.52±0.32	16	0.54378	110	
		hsp-110 RNAi	Exp 1	12.30±0.40	18	0.58147	80	
			Exp 2	13.11±0.47	18	0.69735	76	
			Exp 3	12.31±0.54	17	0.69899	68	
		xbp-1 RNAi	Exp 1	11.20±0.41	17	0.00027	65	Fig.EV4B
			Exp 2	11.15±0.13	17	0.00098	69	
			Exp 3	10.31±0.22	16	0.00086	85	
		atf-6 RNAi	Exp 1	11.59±0.36	16	0.00089	102	
			Exp 2	11.25±0.24	17	0.00024	88	
			Exp 3	11.01±0.12	17	0.00049	74	
		ire-1 RNAi	Exp 1	11.13±0.55	17	0.00075	69	
			Exp 2	11.42±0.28	16	0.00045	91	
			Exp 3	11.03±0.67	16	0.0006	114	Fig.EV4C
		sel-1 RNAi	Exp 1	12.66±0.53	21	0.92478	95	
			Exp 2	12.32±0.13	19	0.42334	68	
			Exp 3	12.40±0.19	20	0.70827	77	
		sel-11 RNAi	Exp 1	10.85±0.55	15	0.00071	83	
			Exp 2	10.94±0.21	16	0.00098	94	
			Exp 3	11.16±0.08	16	0.00007	97	
him-5(e1490)		*L4440*	Exp 1	10.15±0.55	14	0.04562	93	
			Exp 2	10.64±0.21	15	0.01278	85	
			Exp 3	10.36±0.08	14	0.01895	80	
him-5(e1490) Vector	Hermaphrodite		Exp 1	15.03±0.12	22		68	Fig.EV5
			Exp 2	15.53±0.27	23		66	
			Exp 3	15.16±0.38	22		69	
C18E9.2(S301E); him-5(e1490) Vector			Exp 1	13.22±0.13	20	0.00071	74	
			Exp 2	13.15±0.29	20	0.00098	81	
			Exp 3	12.98±0.82	20	0.00072	99	
C18E9.2(S301E); him-5(e1490);unc-23(oe)			Exp 1	16.77±0.18	23	0.00087	124	
			Exp 2	17.25±0.82	24	0.00064	85	
			Exp 3	16.97±0.54	24	0.0008	63	
daf-18(ok480)	Hermaphrodite		Exp 1	10.35±0.23	15		96	Fig.EV6B
			Exp 2	10.11±0.16	15		117	
			Exp 3	1048±0.33	15		85	
daf-18(ok480); unc-23(e25)	Hermaphrodite	L4440 RNAi	Exp 1	10.26±0.58	15	0.07782	49	
			Exp 2	9.98±0.36	15	0.94274	156	

			Exp 3	10.56±0.17	15	0.21953	105	
		tra-2 RNAi	Exp 1	10.25±0.59	15	0.29834	73	
			Exp 2	10.18±0.54	15	0.33101	55	
			Exp 3	10.62±0.47	15	0.68255	88	
daf-18(ok480)	Male		Exp 1	12.38±0.15	17		94	Fig.EV 6C
			Exp 2	12.28±0.19	17		76	
			Exp 3	12.57±0.31	17		101	
daf-18(ok480); her-1(n695)	Male	*L2528*	Exp 1	9.56±0.24	14	0.00052	72	
			Exp 2	8.58±0.21	14	0.00023	96	
			Exp 3	8.89±0.11	14	0.00009	59	
		unc-23 (oe)	Exp 1	12.96±0.03	19	0.00066	63	
			Exp 2	13.01±0.51	22	0.00074	76	
			Exp 3	12.84±0.48	21	0.00082	74	

Control for RNAi: Feeding RNAi control clones with empty vector L4440. Control for overexpression: the transgenic injection strains with empty expression vector L2528. Mean lifespans were calculated by using Kaplan-Meier method; *P* values were determined using the log-rank test; SEM: standard error of the mean; N: number of worms used.

Appendix Table S5. The results of western blot.

			Relative fold change				
			Exp 1	Exp2	Exp3	*P* value	
K48 linked proteins	Hermaphrodites	*daf-18(ok480); unc-23(e25)control*	1	1	1		Fig.EV6F
		daf-18(ok480); unc-23(e25); tra-2RNAi	0.92	0.98	1.20	0.7153(n.s.)	
	Males	*daf-18(ok480); her-1(n695)control*	1	1	1		
		daf-18(ok480); her-1(n695);unc-23(oe)	2.00	2.04	2.61	0.0035	
			Relative fold change				
			Exp 1	Exp 2	Exp3	*P* value	
	Hermaphrodites	*daf-18(ok480) control*	1	1	1		Fig.EV3C
		daf-18(ok480);unc-23 (oe)	2.41	3.77	4.33	<0.001	

The *P* value was determined by using a two-tailed *t test*. n.s.: no significant difference. Control in RNAi experiments: RNAi control clones containing the empty vector L4440.Control in overexpression experiments: transgenic injection strains with the empty expression vector L2528.

Appendix Table S6. Primers for real- time PCR.

gene	forward	reverse
her-1	ACCCCAATTCGATGTGGTTA	TGCAAACAACACATTTTGAAGA
fem-2	TCTCGACGAACGAATGACTG	CTGATCCATGTCGATTGCAC
fem-3	TTGTACGGCCTTTTCCAATC	GTGGATAAAAAGCGGCGATA
pek-1	GCAGCGTGAACAACACAACT	TGTTGAAAGCTCTGGCAATG
skr-8	CCAACATGGCTATCGGAAAA	TCTCCTTTGCAGCTCTCTCC
F44E5.4	TGTTGCTGGTTGATGTTGCT	TGTTTTGCAGGCTTTTGTTG
F44E5.5	GGATGTGTTGCTGGTTGATG	TGTTTTGCAGGCTTTTGTTG
hsp-70	AGCCCGTTGTTGAGGTTGAA	CCCGTACAGAATGCCCAAGT
hsp-110	GCAATTGCTCTAGCCTACGG	AAGCAACCAATGAAGCCTGT
unc-23	TGATCAGAAACGCATCAAGC	CCATCAAATCTGCTTTCGTTC
tra-3	ACATACAGCCATGCATCCAA	TGTCAACGGTGAAAAATGGA
tra-2	TTCATTGAATGGATGCAGGA	ACAGGACAATTTCCGTTTGC
sel-1	GCTCAATTGGGACTCGGACA	CACTTCCTGACTCAGCAGCA
sel-11	CCTCAACATCCTCAACCGCT	CCTAGAAGACGTGCTAGGCG
atf-6	TGTTCAGCCCTTGATGCCAT	TCGGACACTTGTCGAACCAG
xbp-1	TCGCAGCCCAAAATGCTAGA	AAGACGTTCGTTTTCAGCGC
ire-1	GTTCCGGAGAGGCTGTCTTC	GACAGGTTTTGGTGCTCGTG
cdc-42	CTGCTGGACAGGAAGATTACG	CTCGGACATTCTCGAATGAAG
