# Supplementary figures and images for: Male sex determination maintains proteostasis and extends lifespan of daf-18/PTEN deficient C. elegans
Source: EMBO Rep. 2025 Jan 16;26(4):1084–113. doi: 10.1038/s44319-025-00368-x (PMC11850635; doi:10.1038/s44319-025-00368-x)

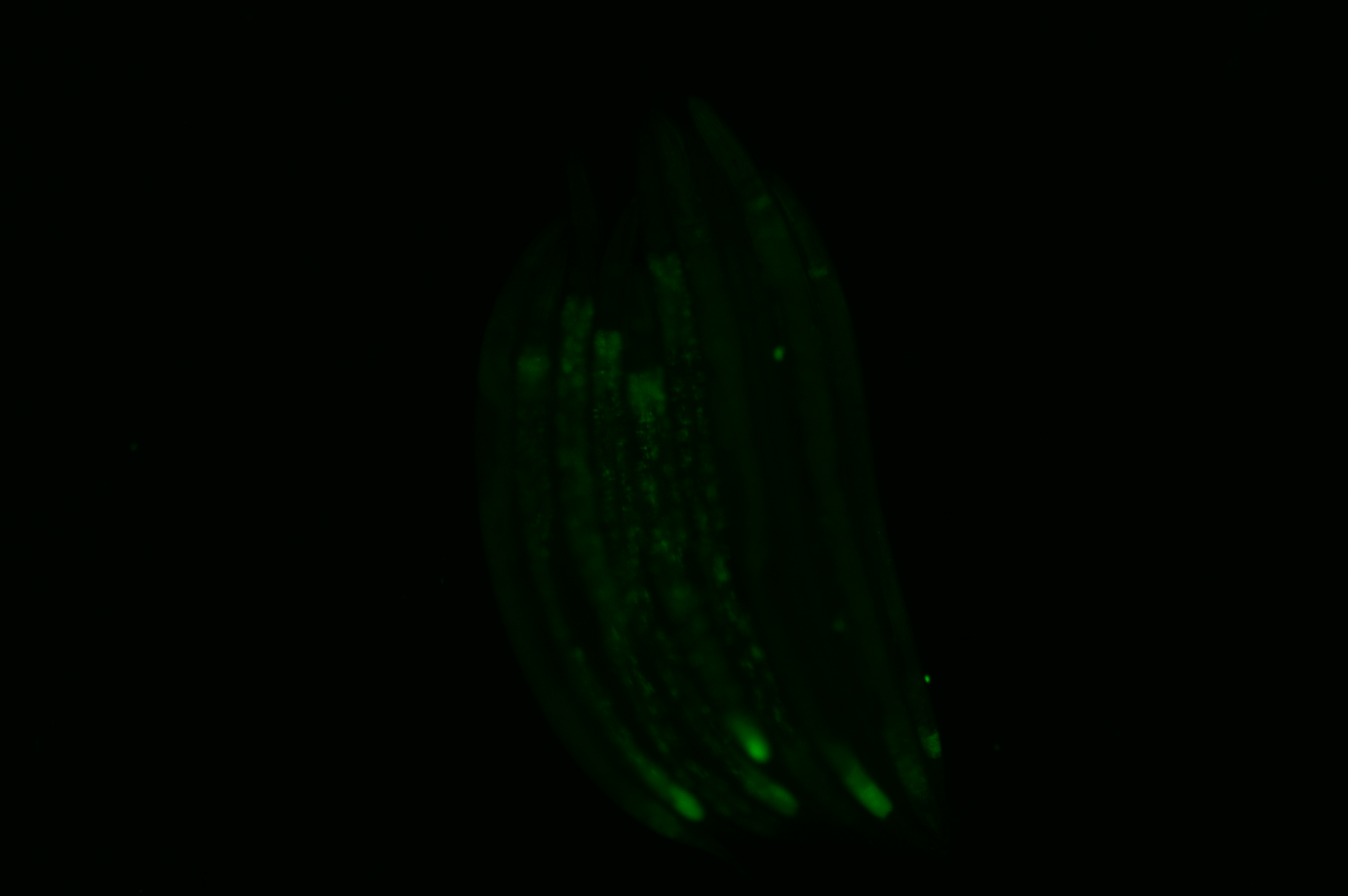

Supplement: Supplementary file 5 — Source data Fig. 3 [file 44319_2025_368_MOESM5_ESM.zip › Fig. 3 source data/3A/control.tif]

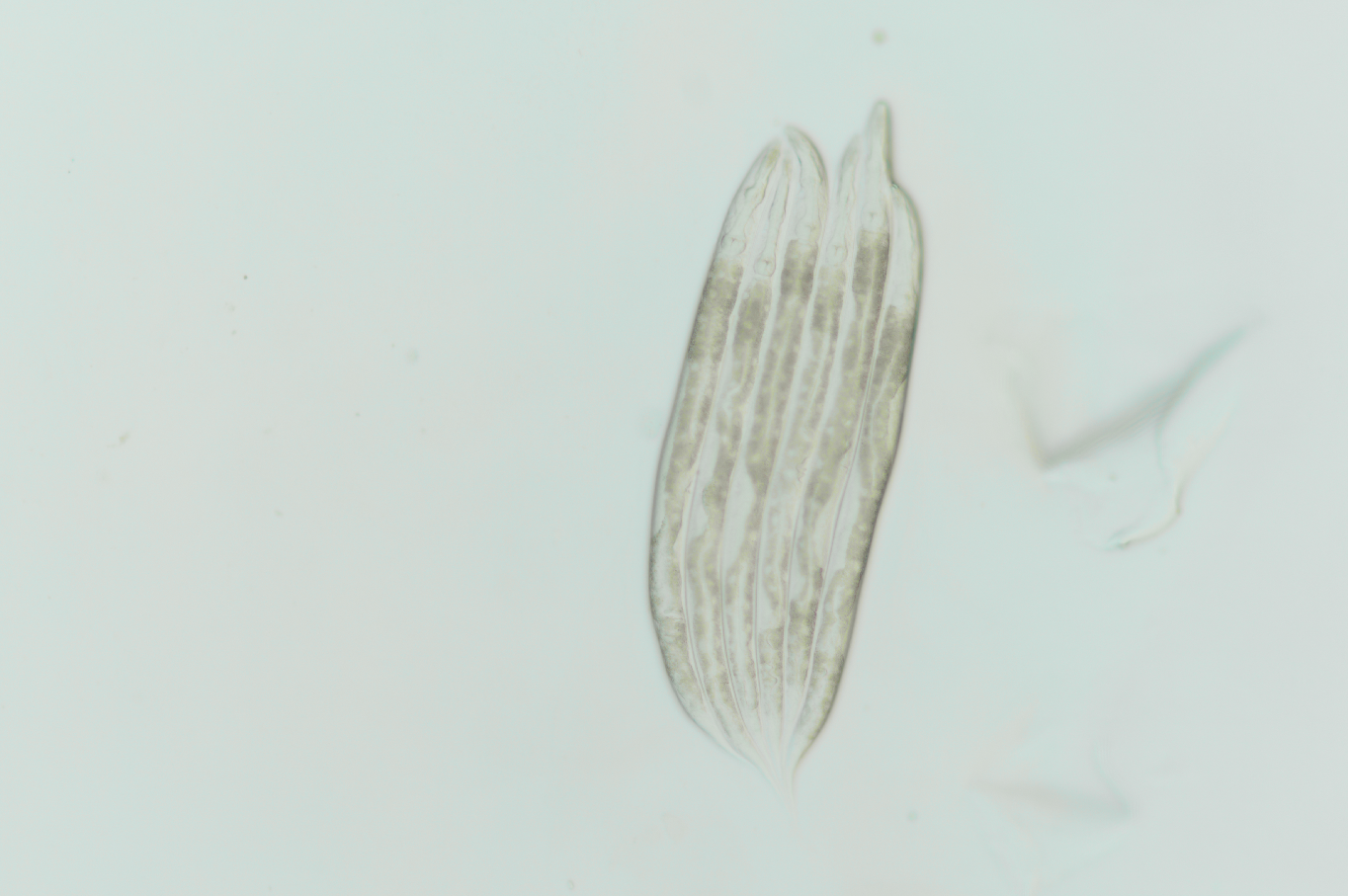

Supplement: Supplementary file 5 — Source data Fig. 3 [file 44319_2025_368_MOESM5_ESM.zip › Fig. 3 source data/3A/daf-18(ok480);control,.tif]

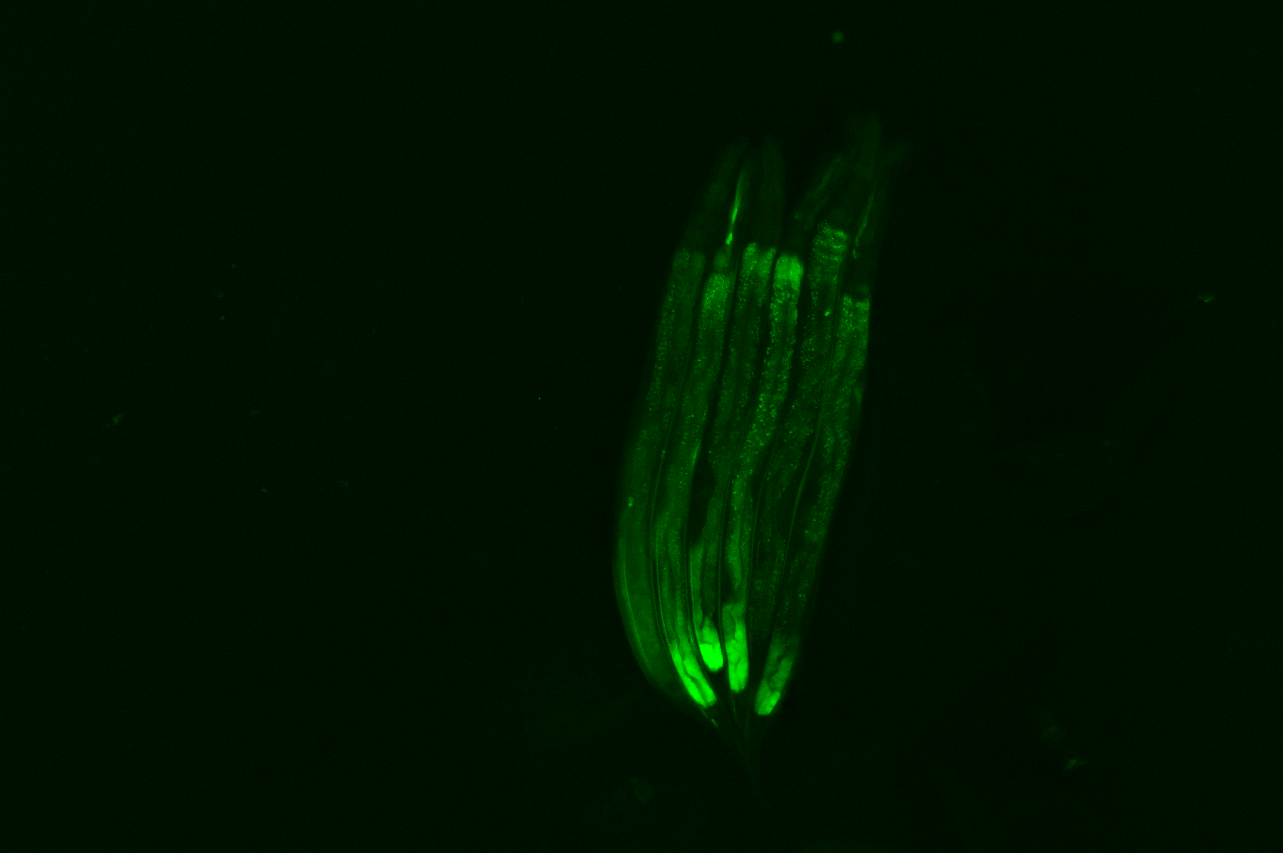

Supplement: Supplementary file 5 — Source data Fig. 3 [file 44319_2025_368_MOESM5_ESM.zip › Fig. 3 source data/3A/daf-18(ok480);control.tif]

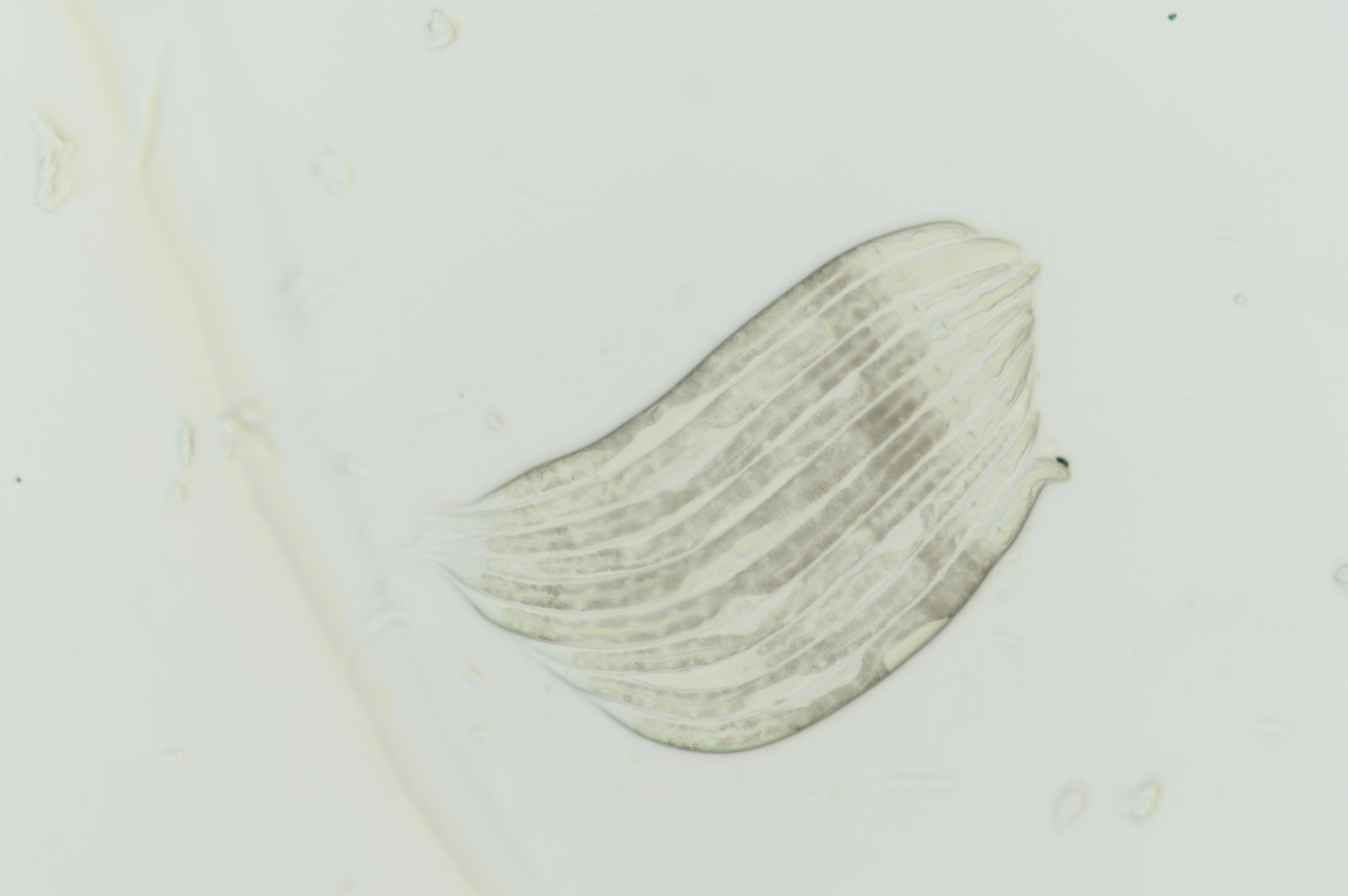

Supplement: Supplementary file 5 — Source data Fig. 3 [file 44319_2025_368_MOESM5_ESM.zip › Fig. 3 source data/3A/daf-18(ok480);unc-23 RNAi,.tif]

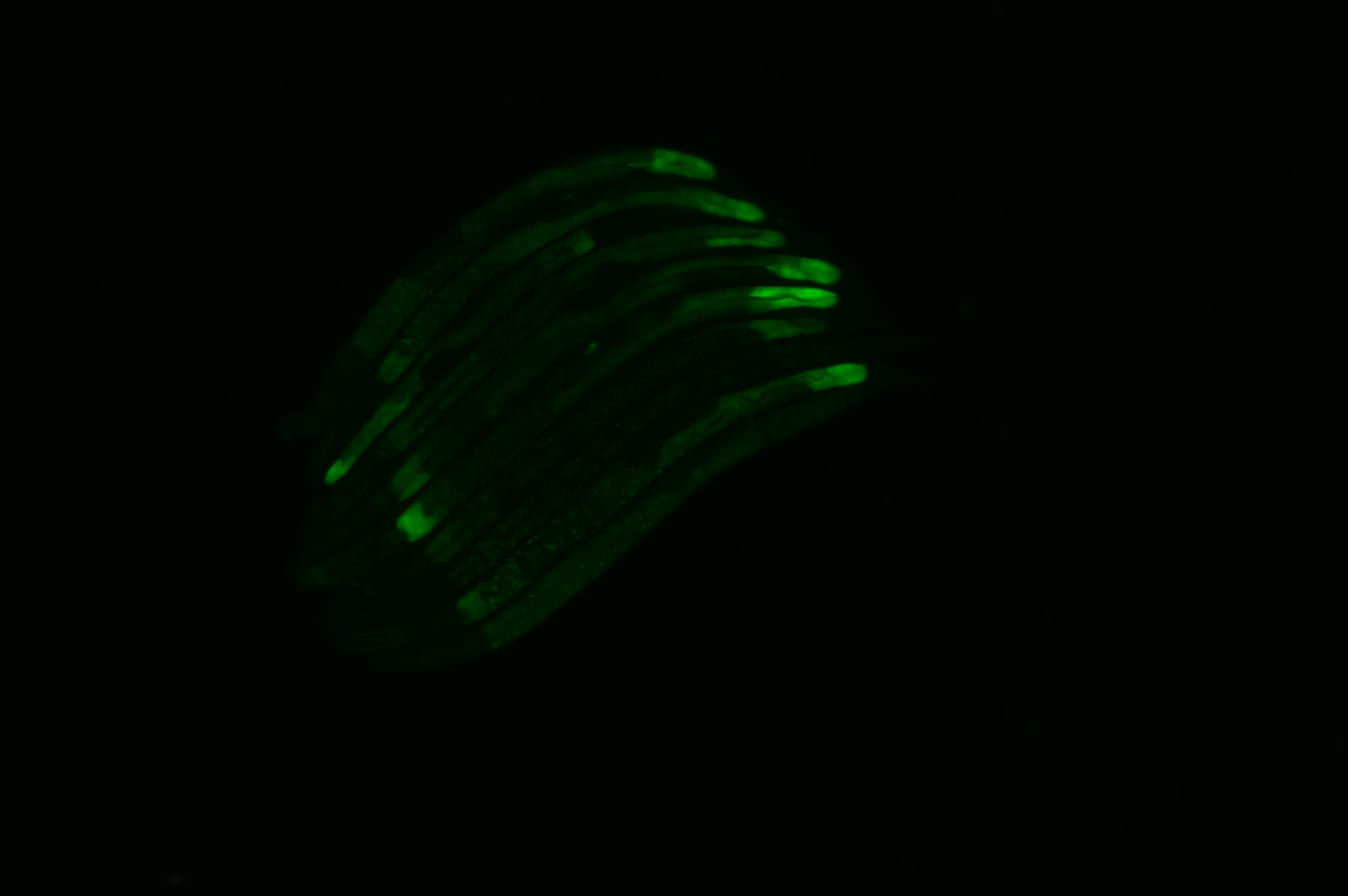

Supplement: Supplementary file 5 — Source data Fig. 3 [file 44319_2025_368_MOESM5_ESM.zip › Fig. 3 source data/3A/daf-18(ok480);unc-23 RNAi.tif]

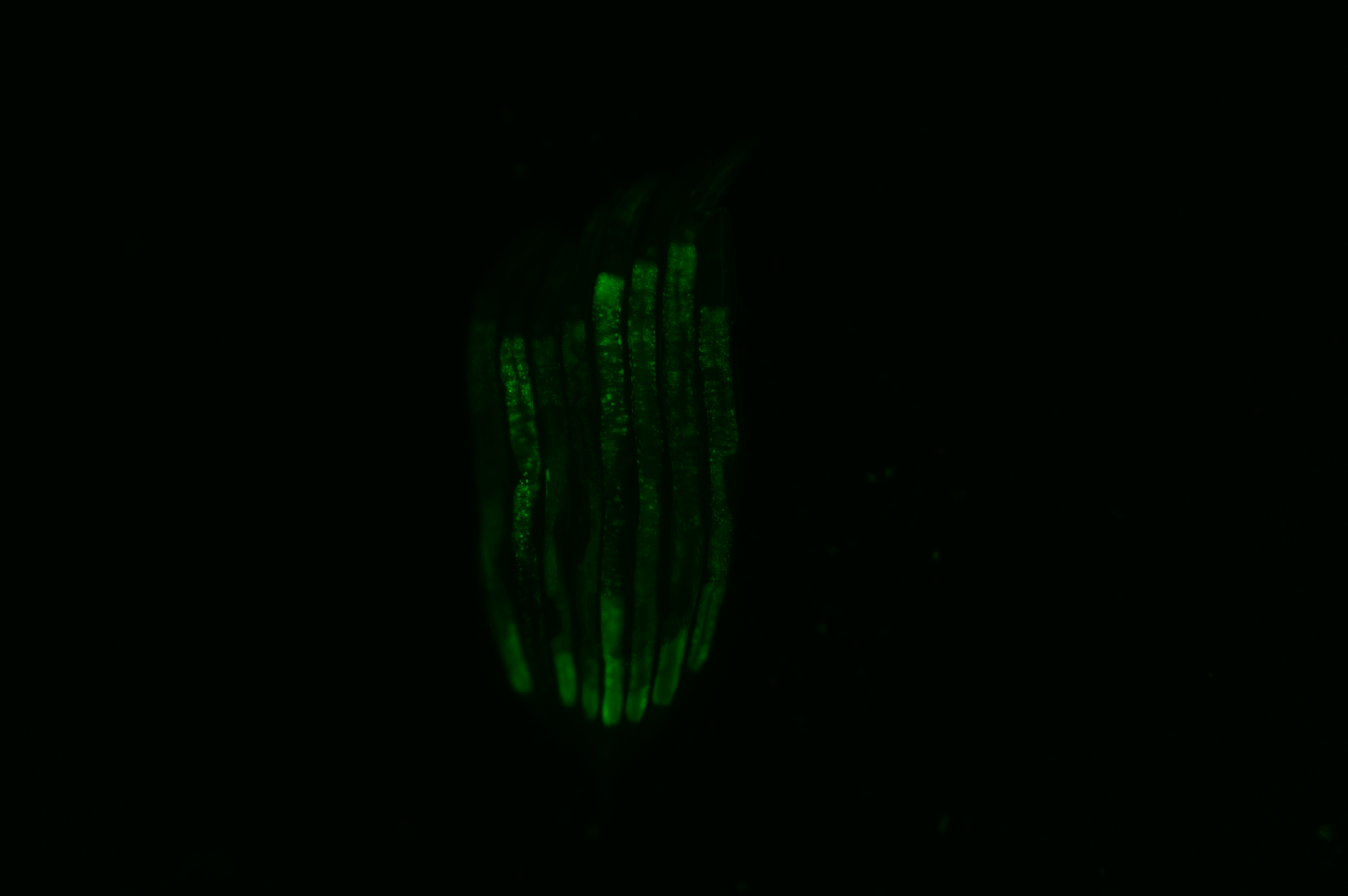

Supplement: Supplementary file 5 — Source data Fig. 3 [file 44319_2025_368_MOESM5_ESM.zip › Fig. 3 source data/3A/unc-23 RNAi,.tif]

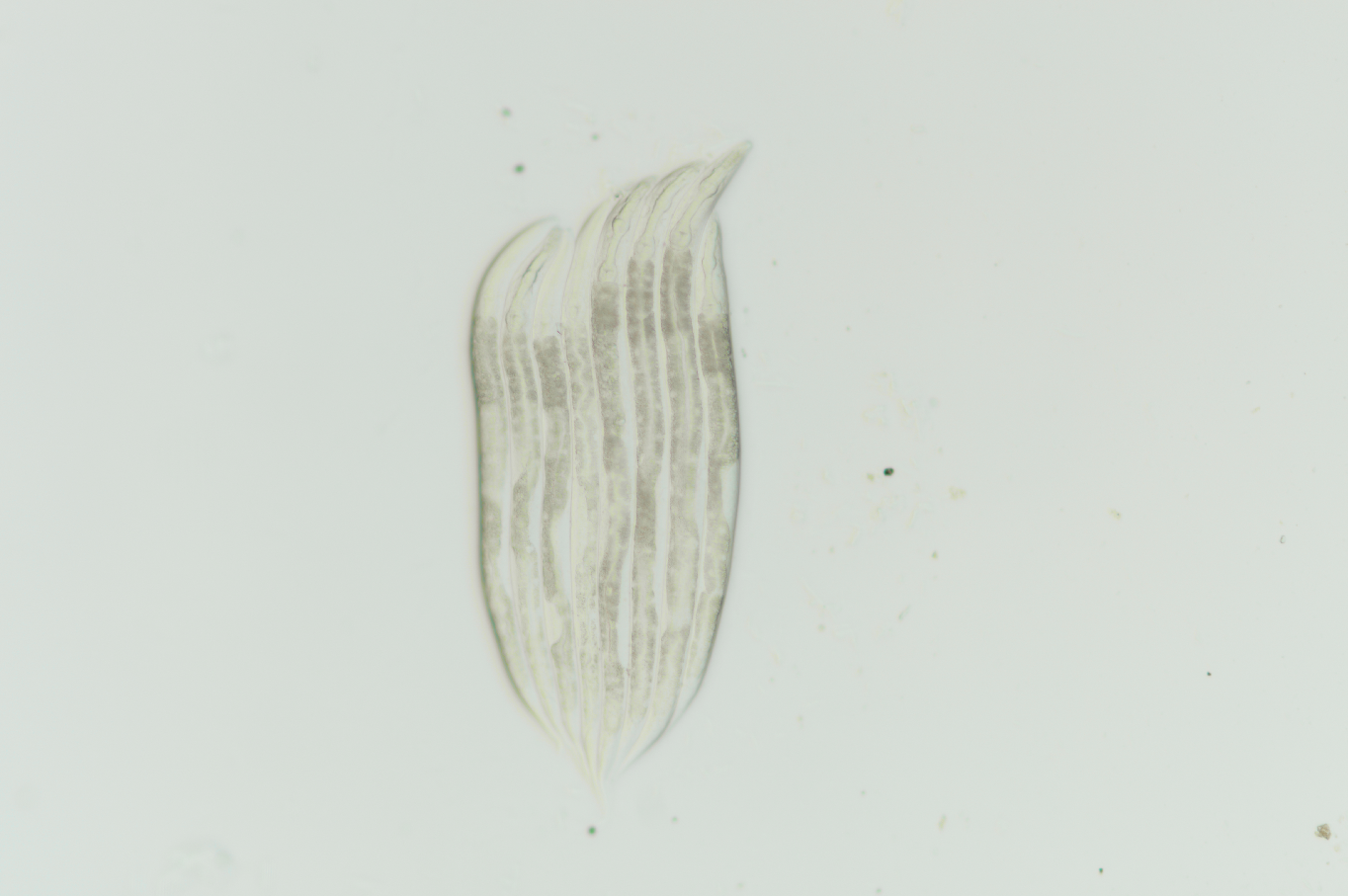

Supplement: Supplementary file 5 — Source data Fig. 3 [file 44319_2025_368_MOESM5_ESM.zip › Fig. 3 source data/3A/unc-23 RNAi.tif]

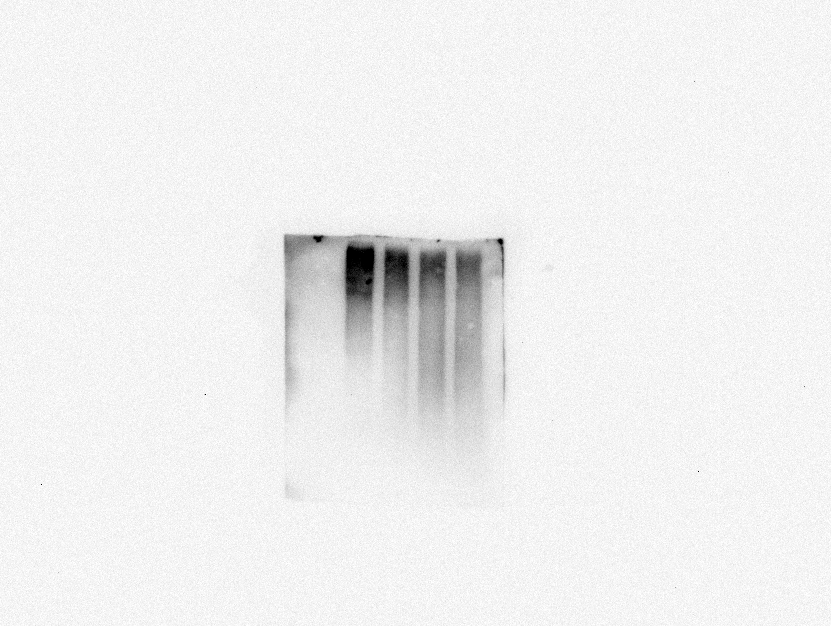

Supplement: Supplementary file 5 — Source data Fig. 3 [file 44319_2025_368_MOESM5_ESM.zip › Fig. 3 source data/3G/Fig.3G-Total Ub.png]

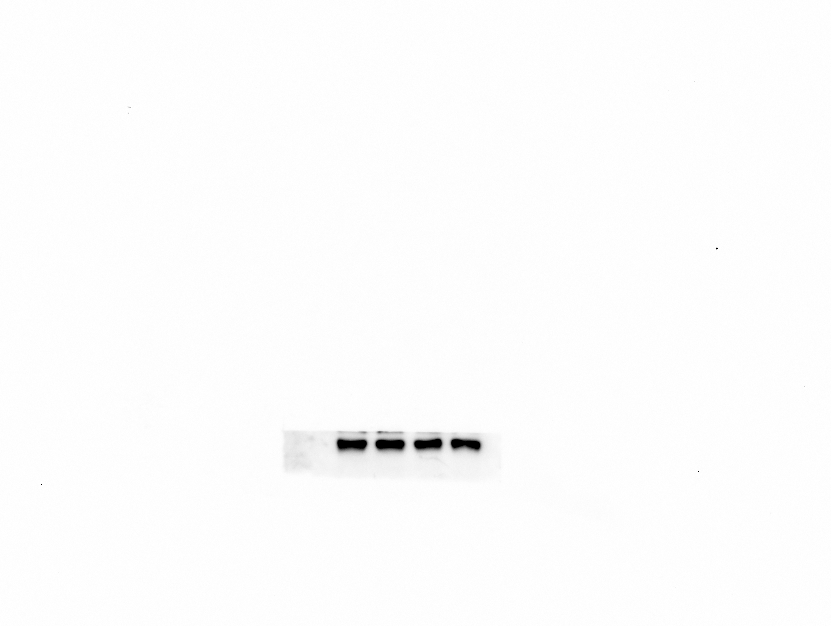

Supplement: Supplementary file 5 — Source data Fig. 3 [file 44319_2025_368_MOESM5_ESM.zip › Fig. 3 source data/3G/Fig.3G-tubulin.png]

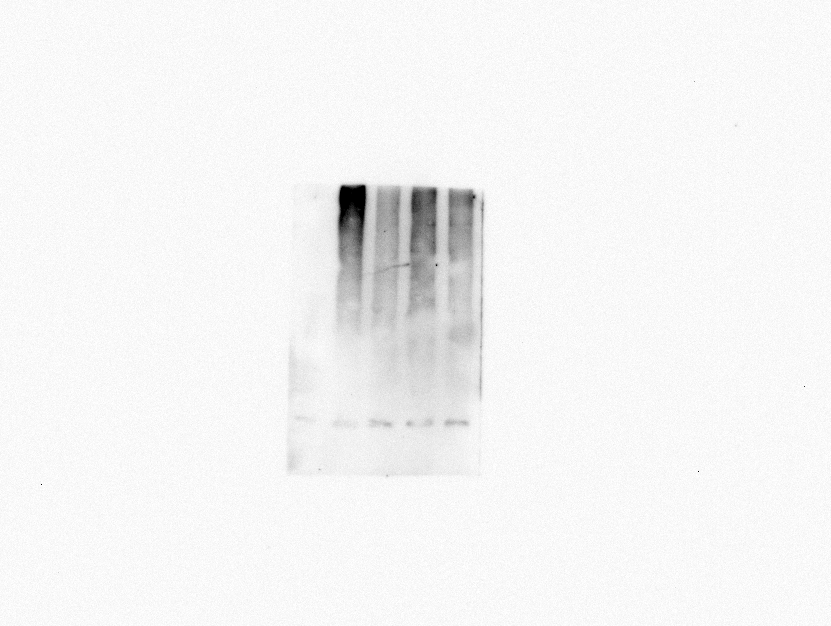

Supplement: Supplementary file 5 — Source data Fig. 3 [file 44319_2025_368_MOESM5_ESM.zip › Fig. 3 source data/3H/Fig.3H-k48.png]

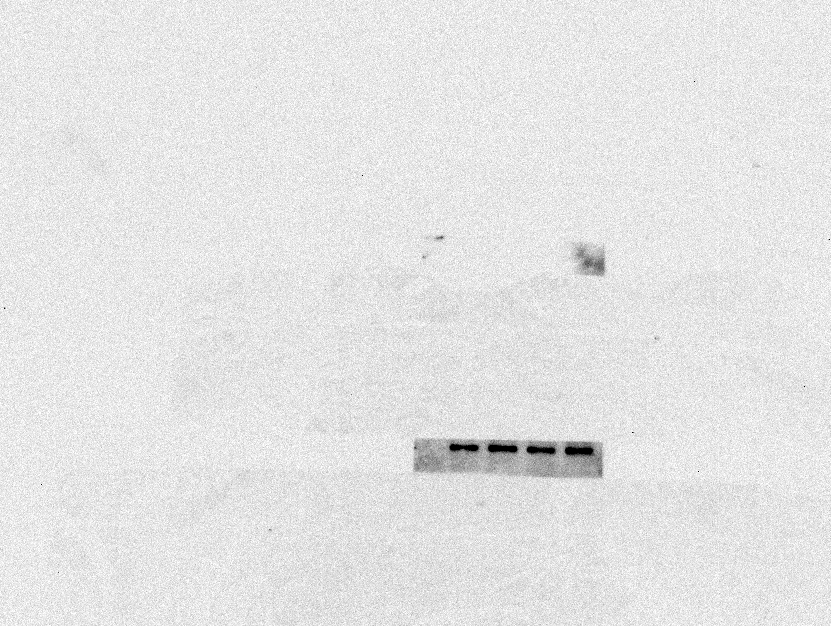

Supplement: Supplementary file 5 — Source data Fig. 3 [file 44319_2025_368_MOESM5_ESM.zip › Fig. 3 source data/3H/Fig.3H-tubulin.png]

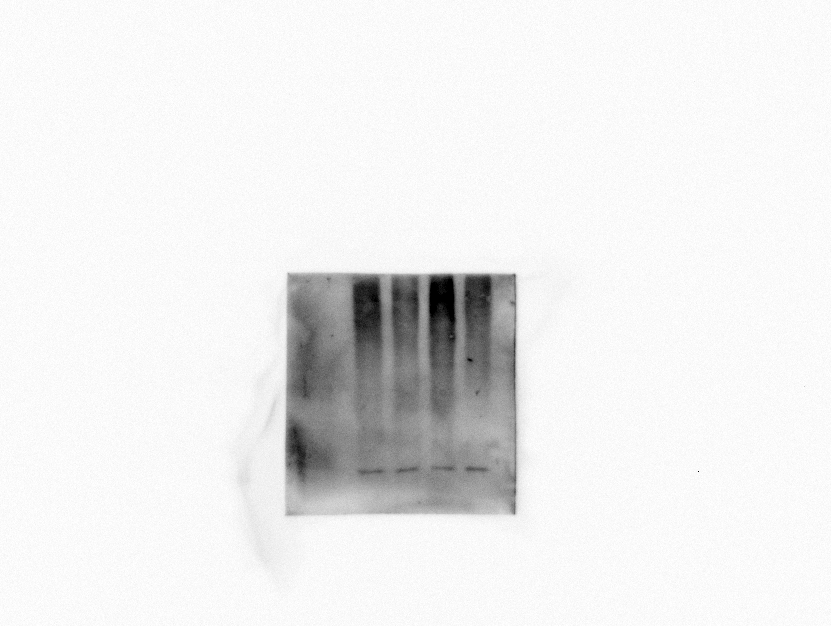

Supplement: Supplementary file 5 — Source data Fig. 3 [file 44319_2025_368_MOESM5_ESM.zip › Fig. 3 source data/3I/Fig. 3I- Total Ub.png]

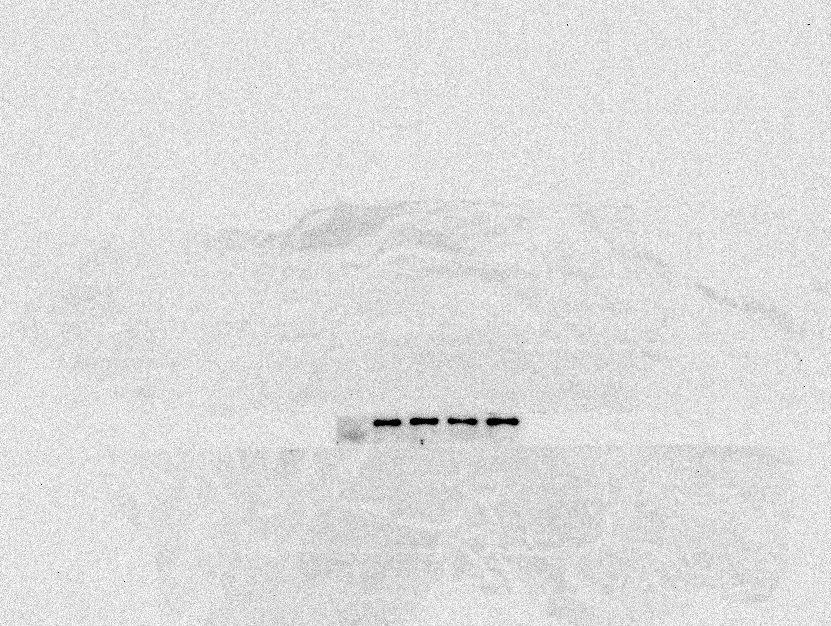

Supplement: Supplementary file 5 — Source data Fig. 3 [file 44319_2025_368_MOESM5_ESM.zip › Fig. 3 source data/3I/Fig. 3I- tubulin.png]

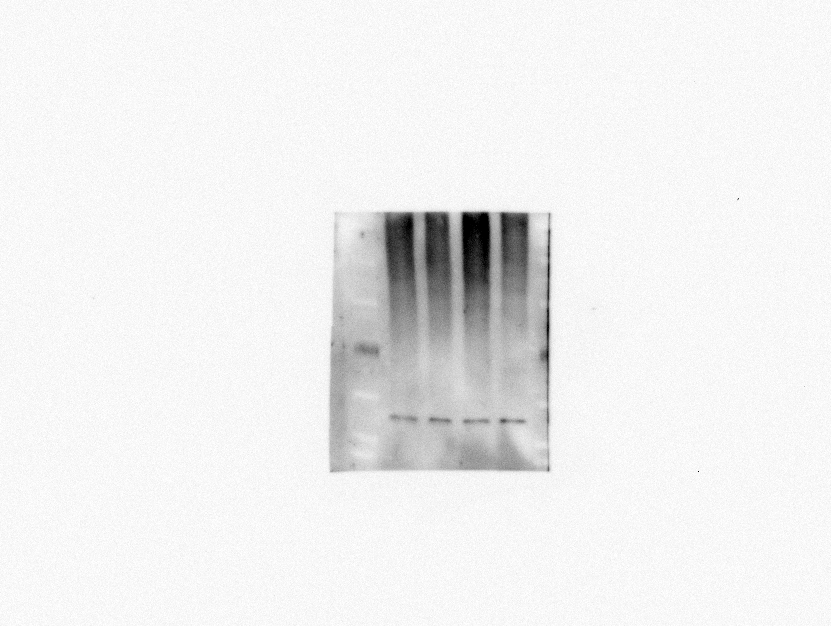

Supplement: Supplementary file 5 — Source data Fig. 3 [file 44319_2025_368_MOESM5_ESM.zip › Fig. 3 source data/3J/Fig.3J-k48.png]

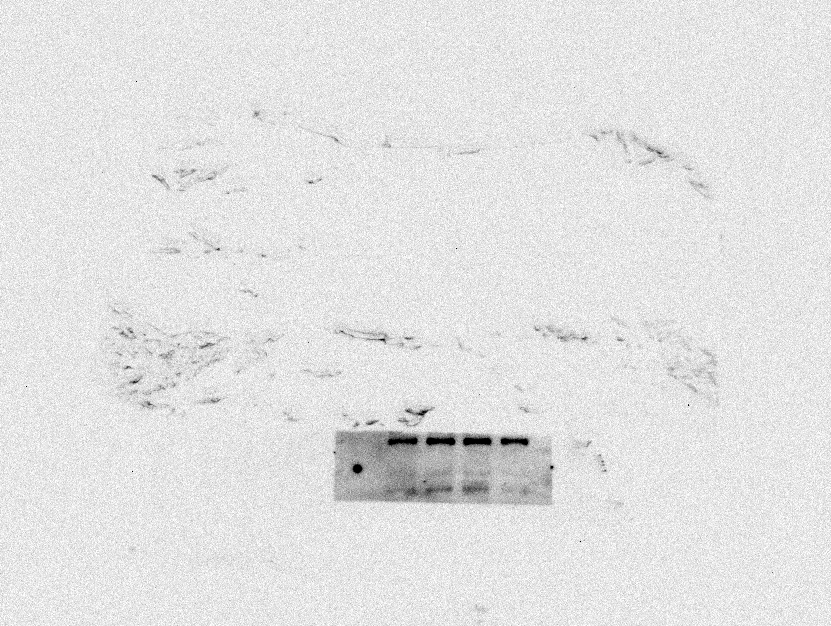

Supplement: Supplementary file 5 — Source data Fig. 3 [file 44319_2025_368_MOESM5_ESM.zip › Fig. 3 source data/3J/Fig.3J-tubulin.png]

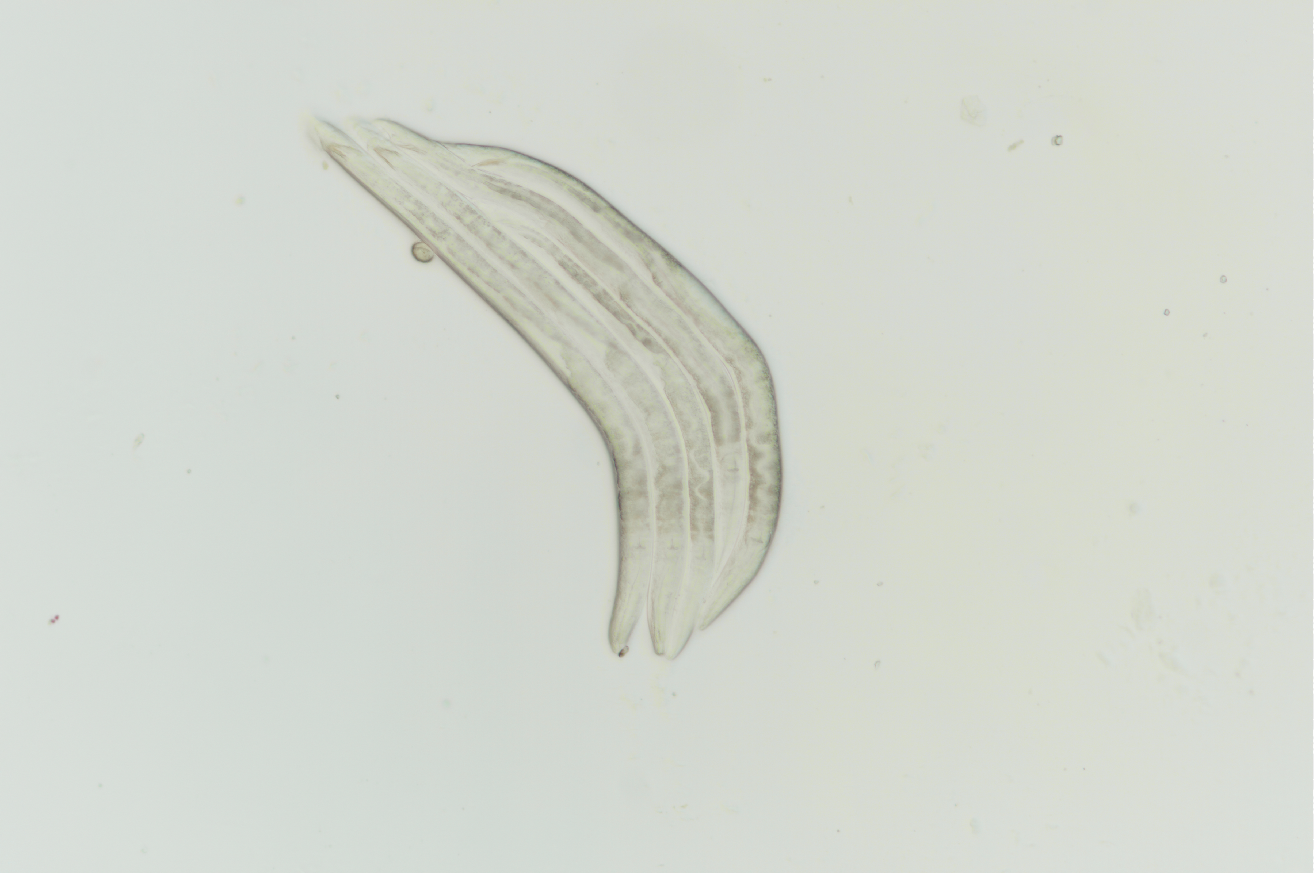

Supplement: Supplementary file 6 — Source data Fig. 3 [file 44319_2025_368_MOESM6_ESM.zip › Fig.3B source figure part 1/control,.tif]

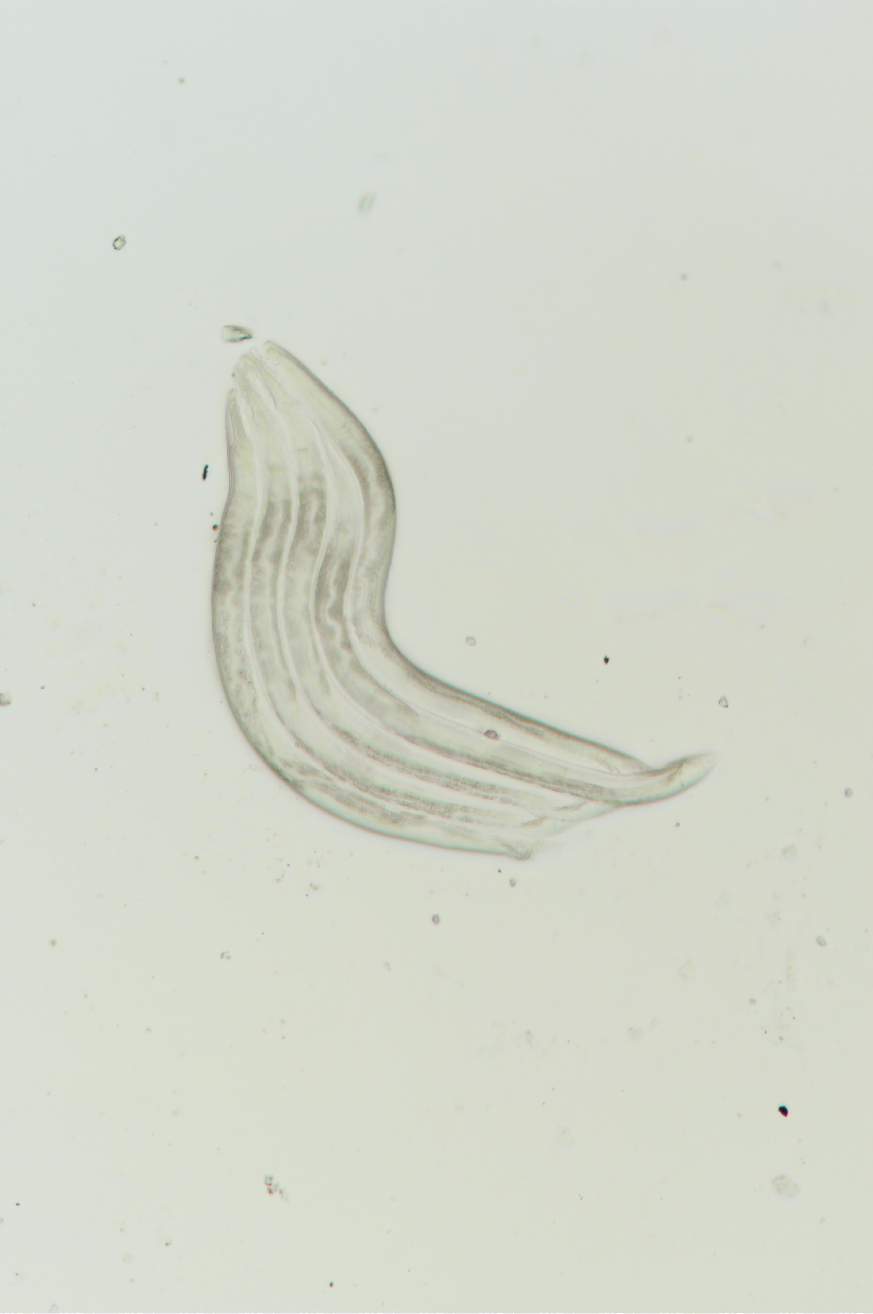

Supplement: Supplementary file 6 — Source data Fig. 3 [file 44319_2025_368_MOESM6_ESM.zip › Fig.3B source figure part 1/daf-18(ok480);control,.tif]

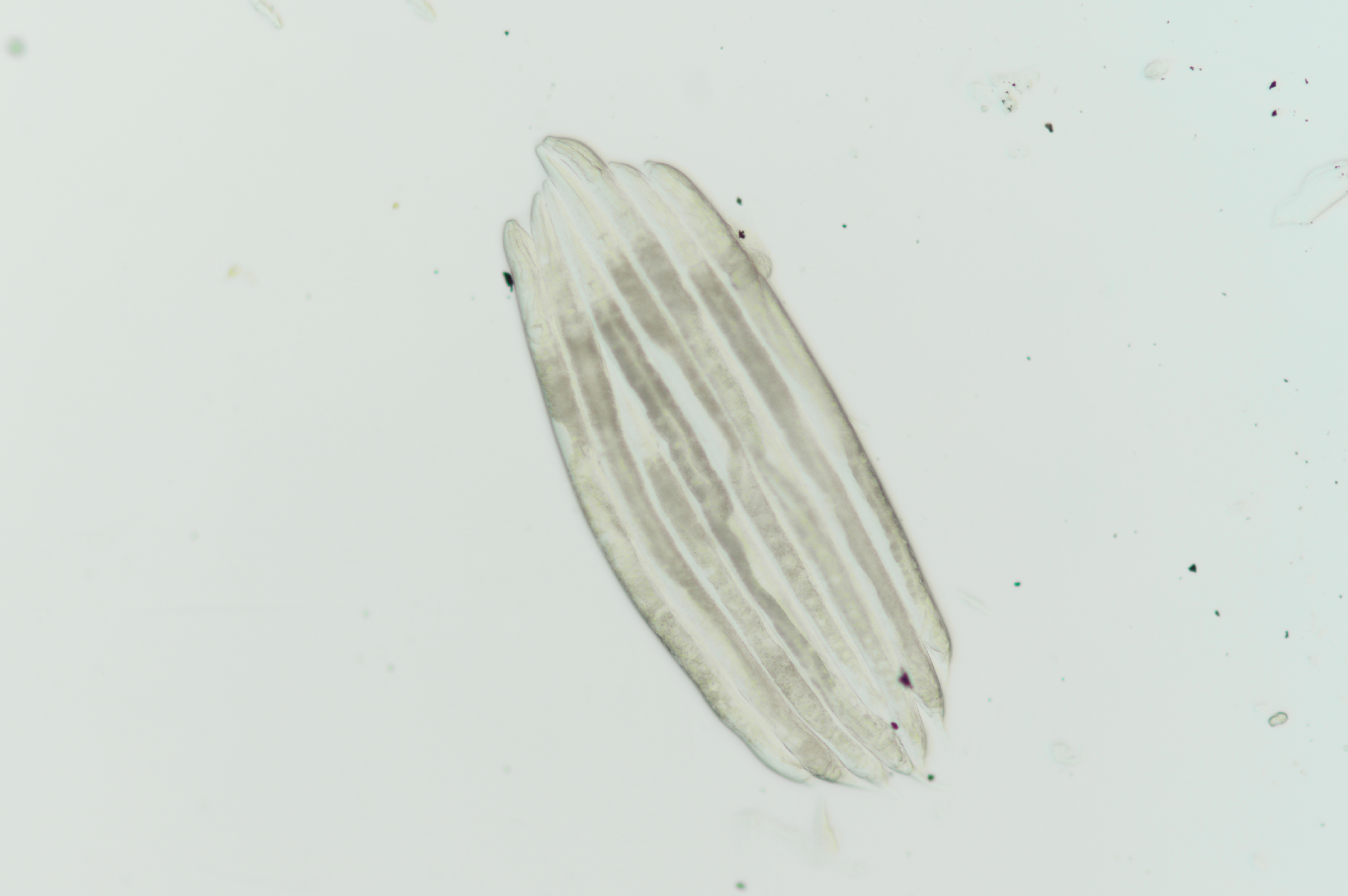

Supplement: Supplementary file 7 — Source data Fig. 3 [file 44319_2025_368_MOESM7_ESM.zip › Fig.3B source figure part 2/daf-18(ok480);unc-23 RNAi,.tif]

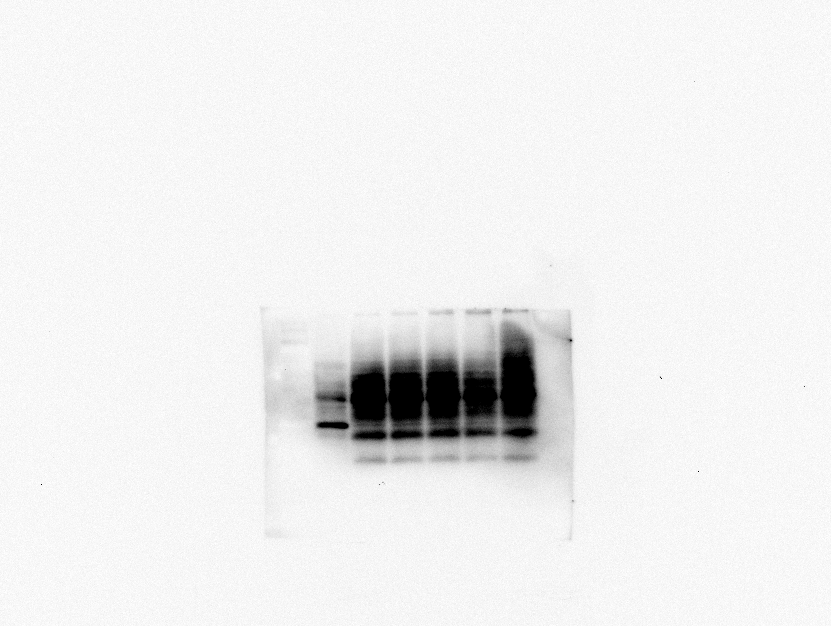

Supplement: Supplementary file 9 — Source data Fig. 5 [file 44319_2025_368_MOESM9_ESM.zip › Fig. 5 source data/5F/Fig.5F- a beta.png]

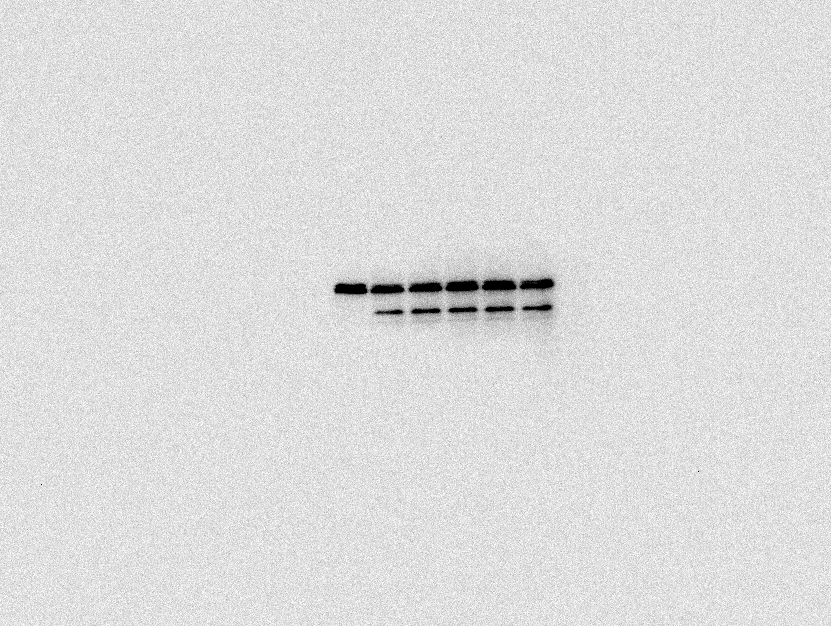

Supplement: Supplementary file 9 — Source data Fig. 5 [file 44319_2025_368_MOESM9_ESM.zip › Fig. 5 source data/5F/Fig.5F-tubulin.png]

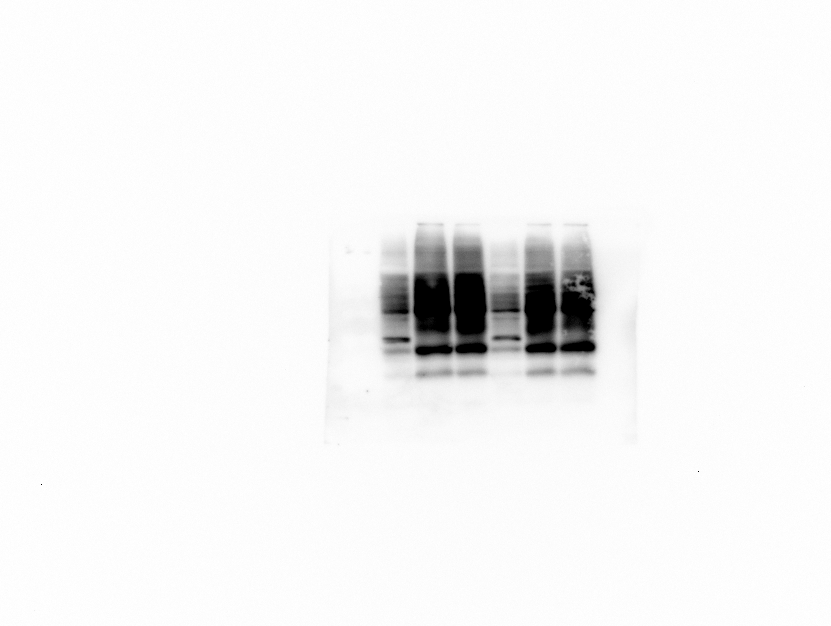

Supplement: Supplementary file 9 — Source data Fig. 5 [file 44319_2025_368_MOESM9_ESM.zip › Fig. 5 source data/5G/Fig.5G-a beta.png]

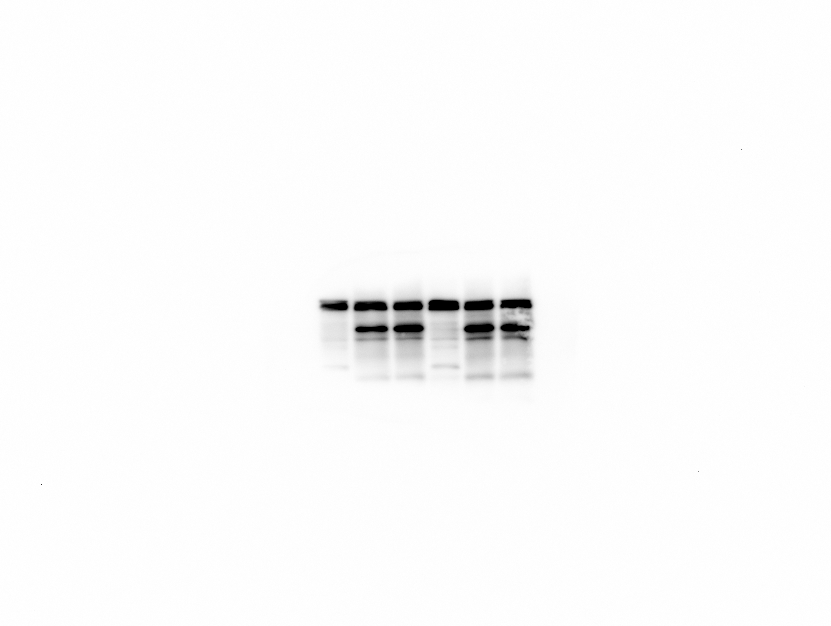

Supplement: Supplementary file 9 — Source data Fig. 5 [file 44319_2025_368_MOESM9_ESM.zip › Fig. 5 source data/5G/Fig.5G-tubulin.png]

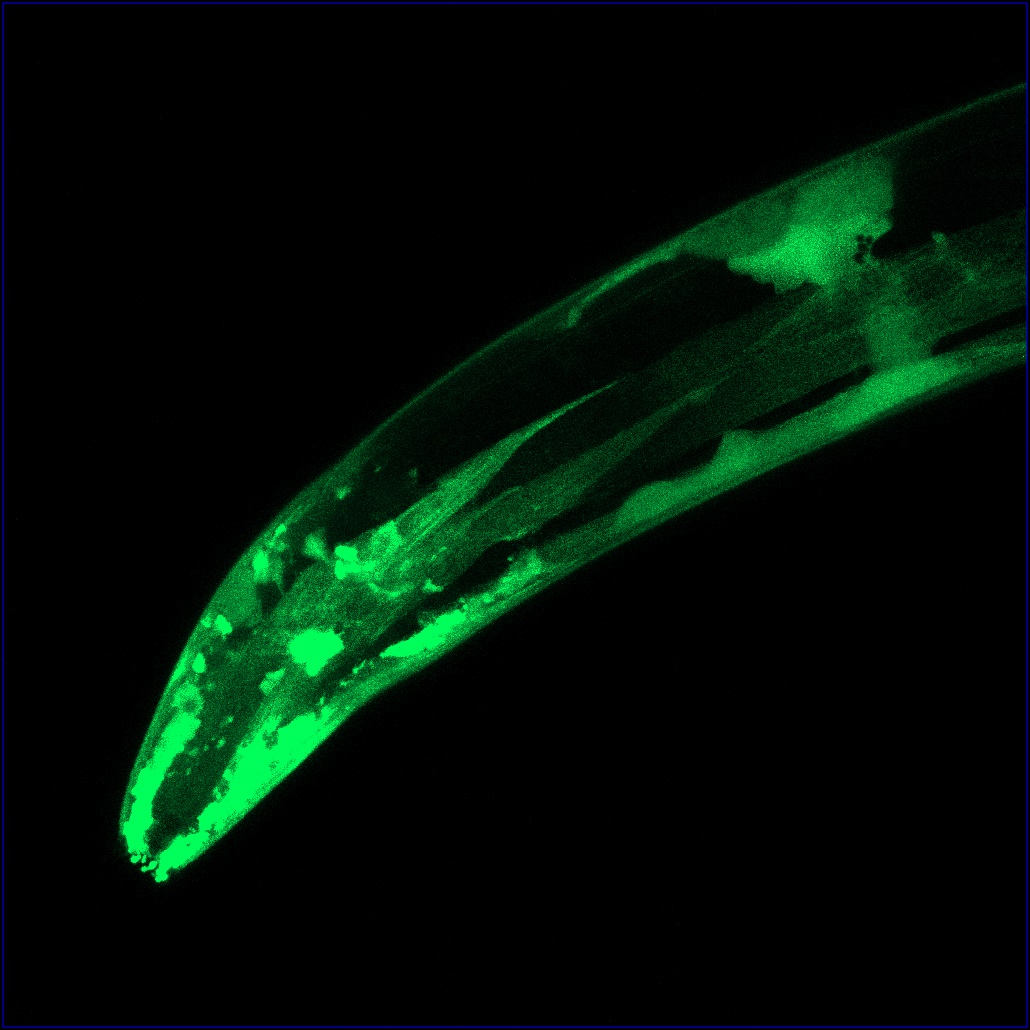

Supplement: Supplementary file 9 — Source data Fig. 5 [file 44319_2025_368_MOESM9_ESM.zip › Fig. 5 source data/5H/control+50 uM MG132.jpg]

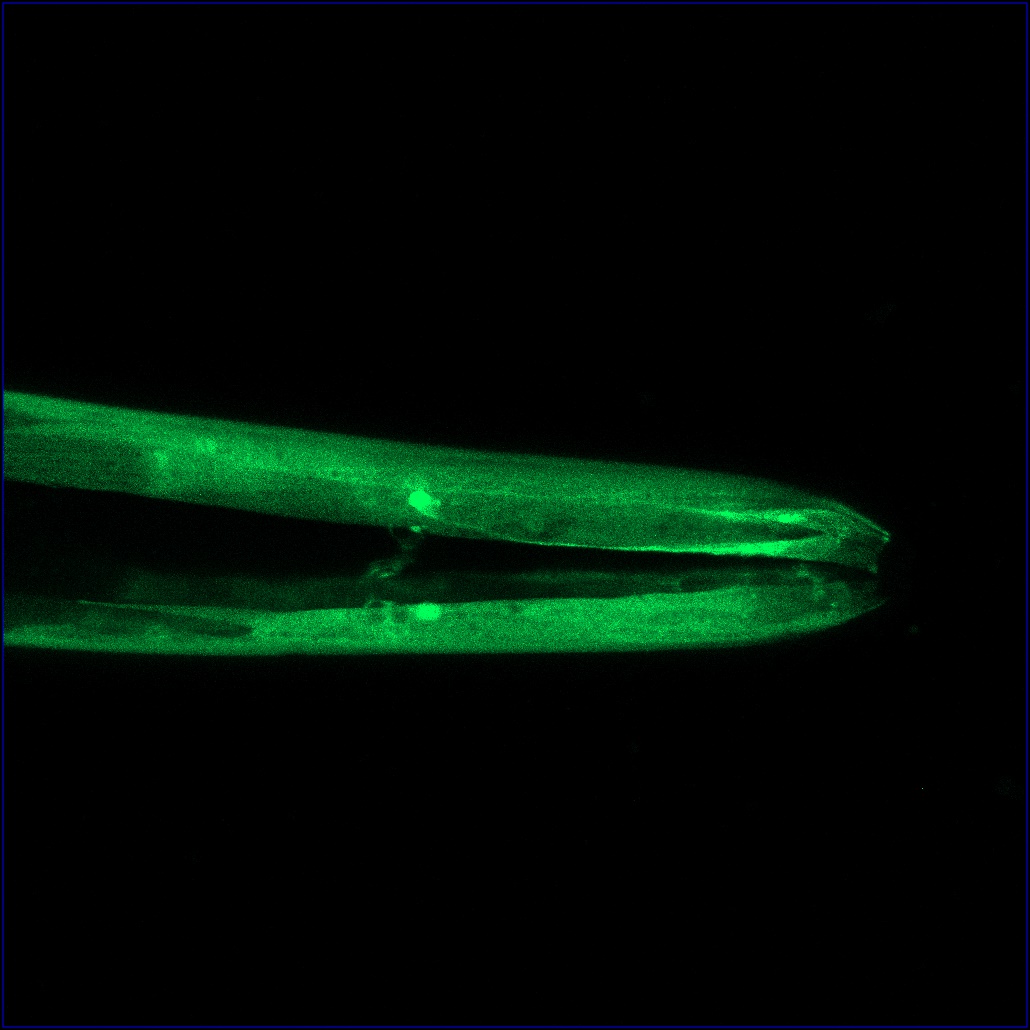

Supplement: Supplementary file 9 — Source data Fig. 5 [file 44319_2025_368_MOESM9_ESM.zip › Fig. 5 source data/5H/control.jpg]

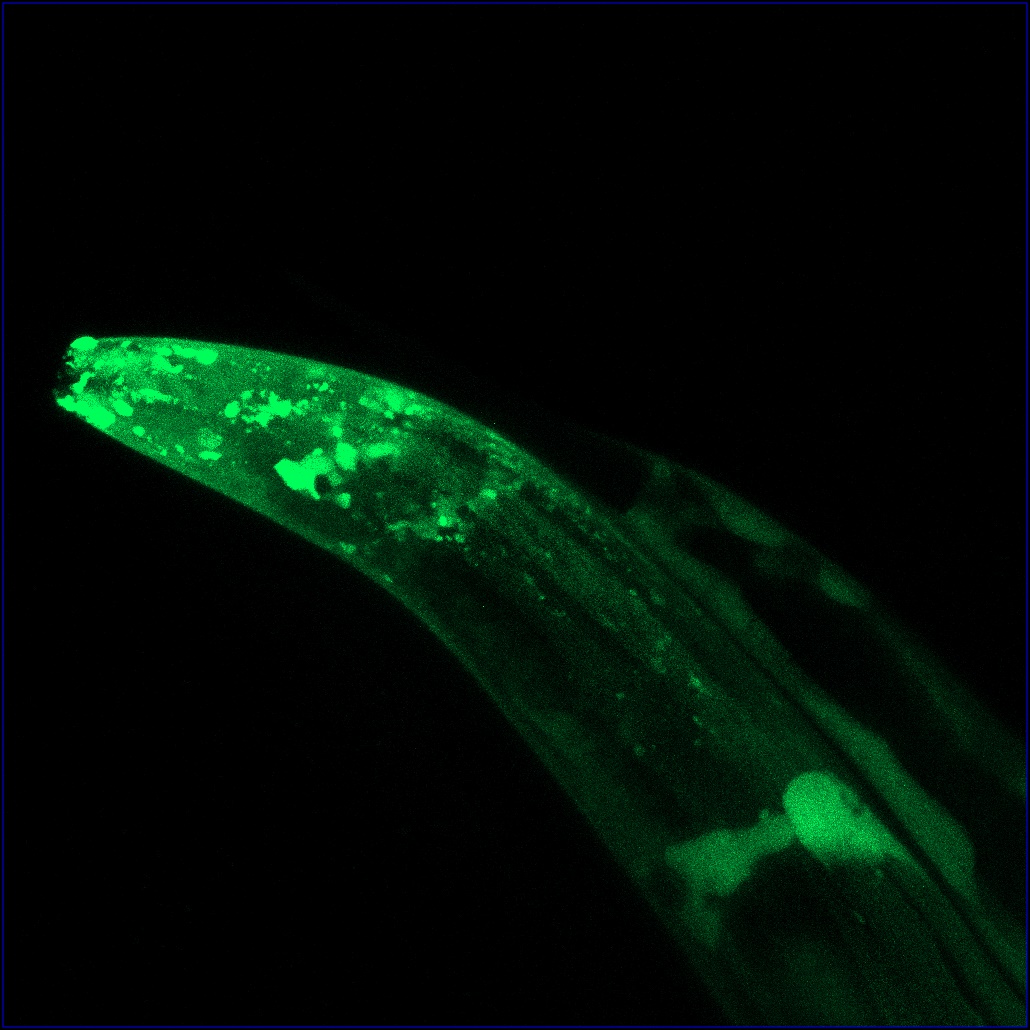

Supplement: Supplementary file 9 — Source data Fig. 5 [file 44319_2025_368_MOESM9_ESM.zip › Fig. 5 source data/5H/daf-18(D137A)+50uM MG132.jpg]

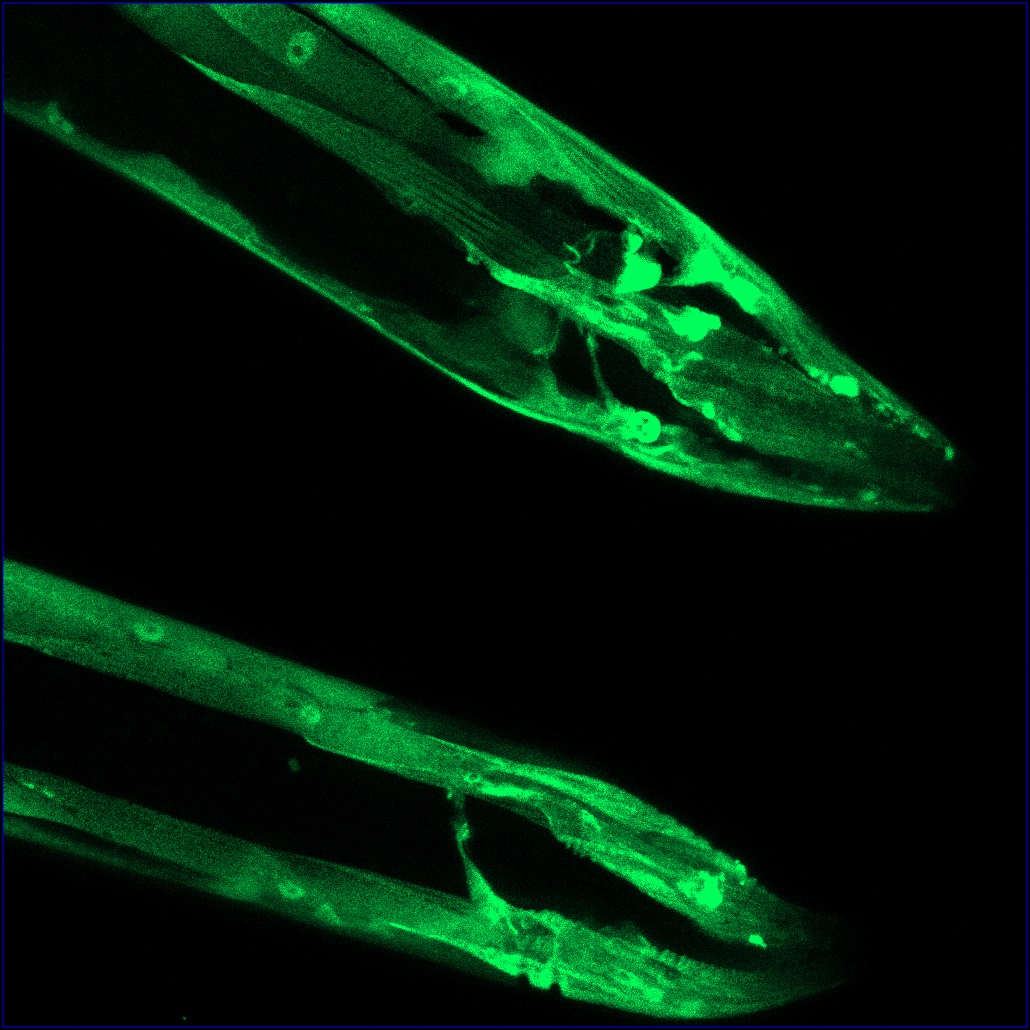

Supplement: Supplementary file 9 — Source data Fig. 5 [file 44319_2025_368_MOESM9_ESM.zip › Fig. 5 source data/5H/daf-18(D137A).jpg]

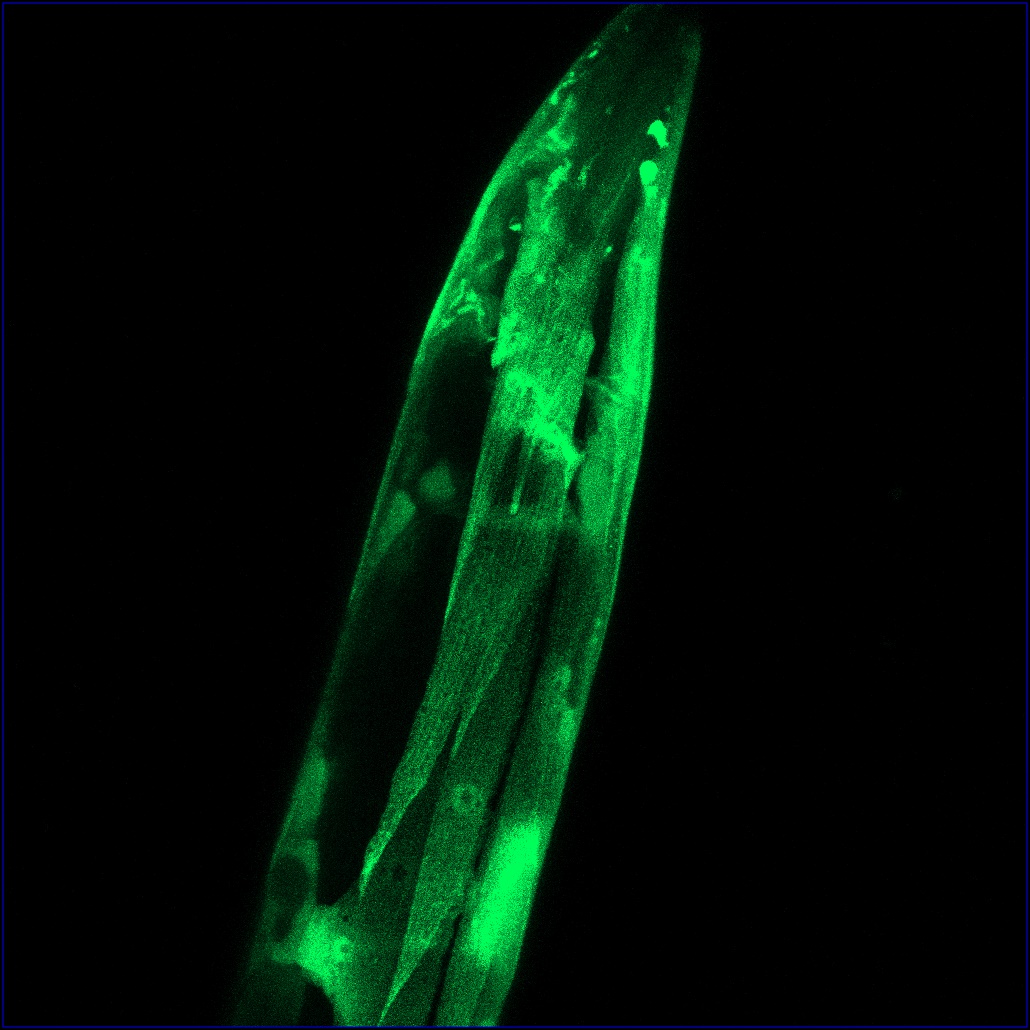

Supplement: Supplementary file 9 — Source data Fig. 5 [file 44319_2025_368_MOESM9_ESM.zip › Fig. 5 source data/5H/daf-18(D137A);unc-23 RNAi.jpg]

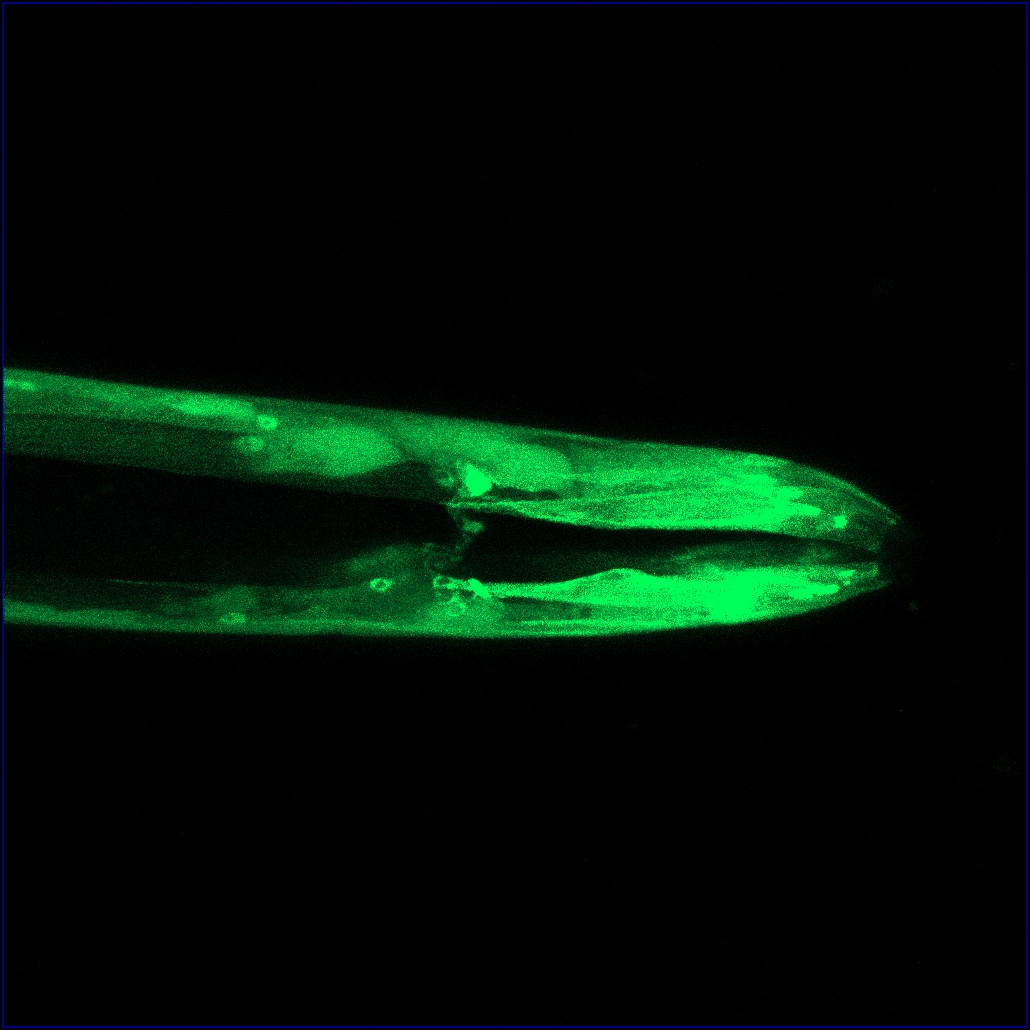

Supplement: Supplementary file 9 — Source data Fig. 5 [file 44319_2025_368_MOESM9_ESM.zip › Fig. 5 source data/5H/unc-23 RNAi.jpg]

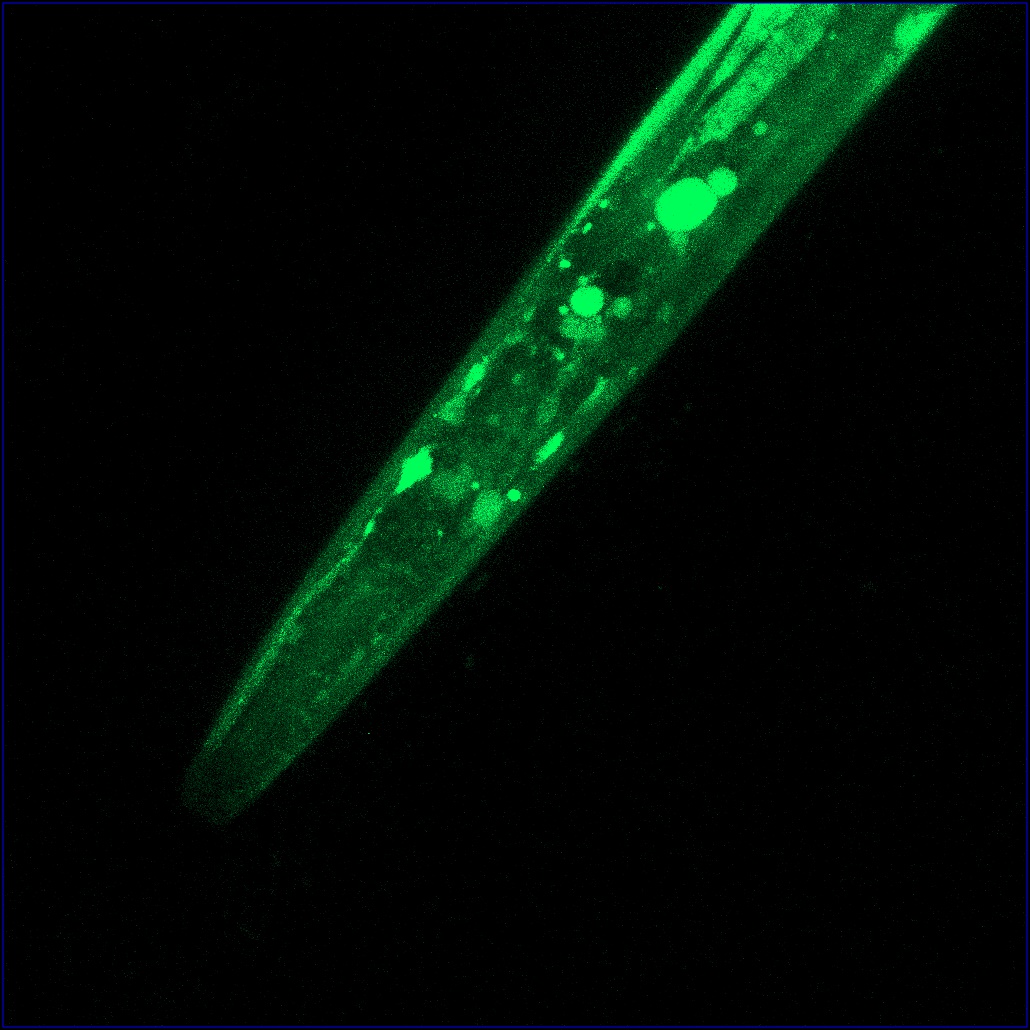

Supplement: Supplementary file 9 — Source data Fig. 5 [file 44319_2025_368_MOESM9_ESM.zip › Fig. 5 source data/5I/control + 50uM MG132.jpg]

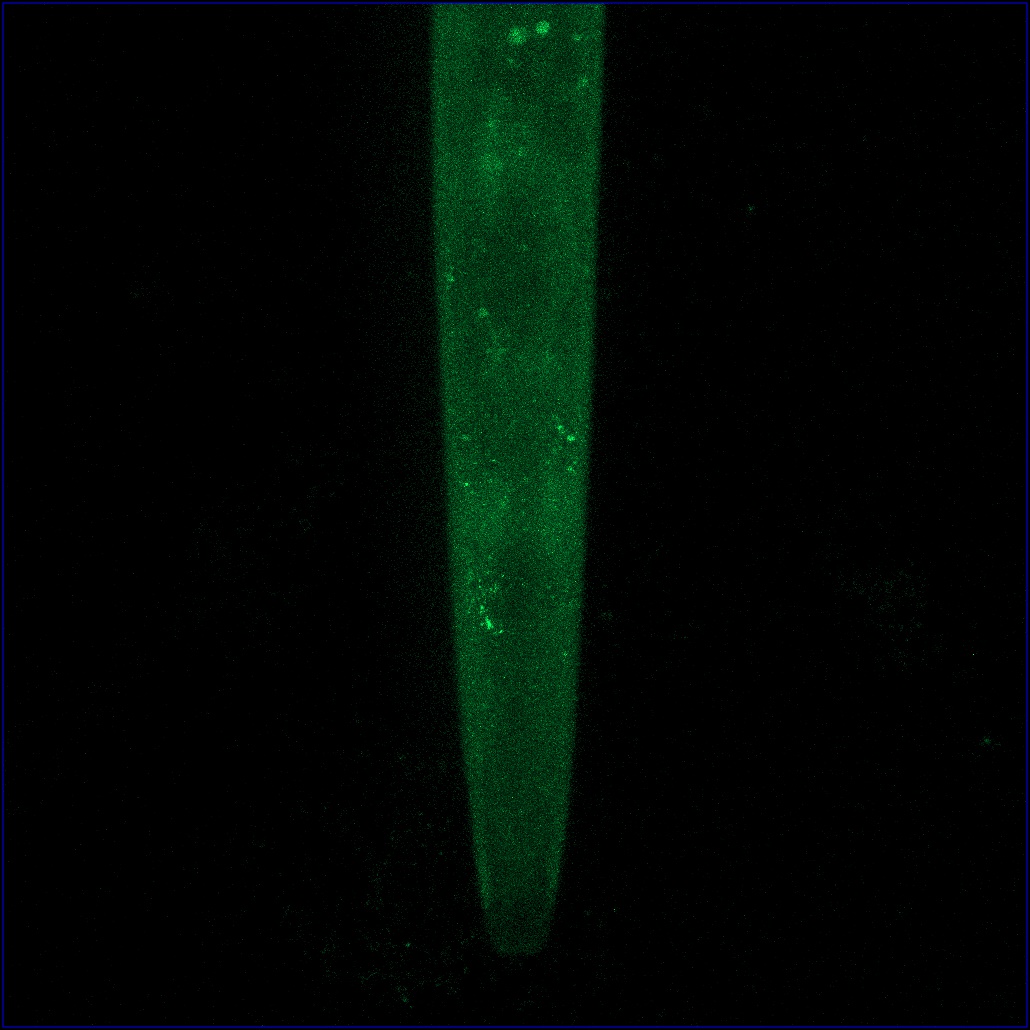

Supplement: Supplementary file 9 — Source data Fig. 5 [file 44319_2025_368_MOESM9_ESM.zip › Fig. 5 source data/5I/Control.jpg]

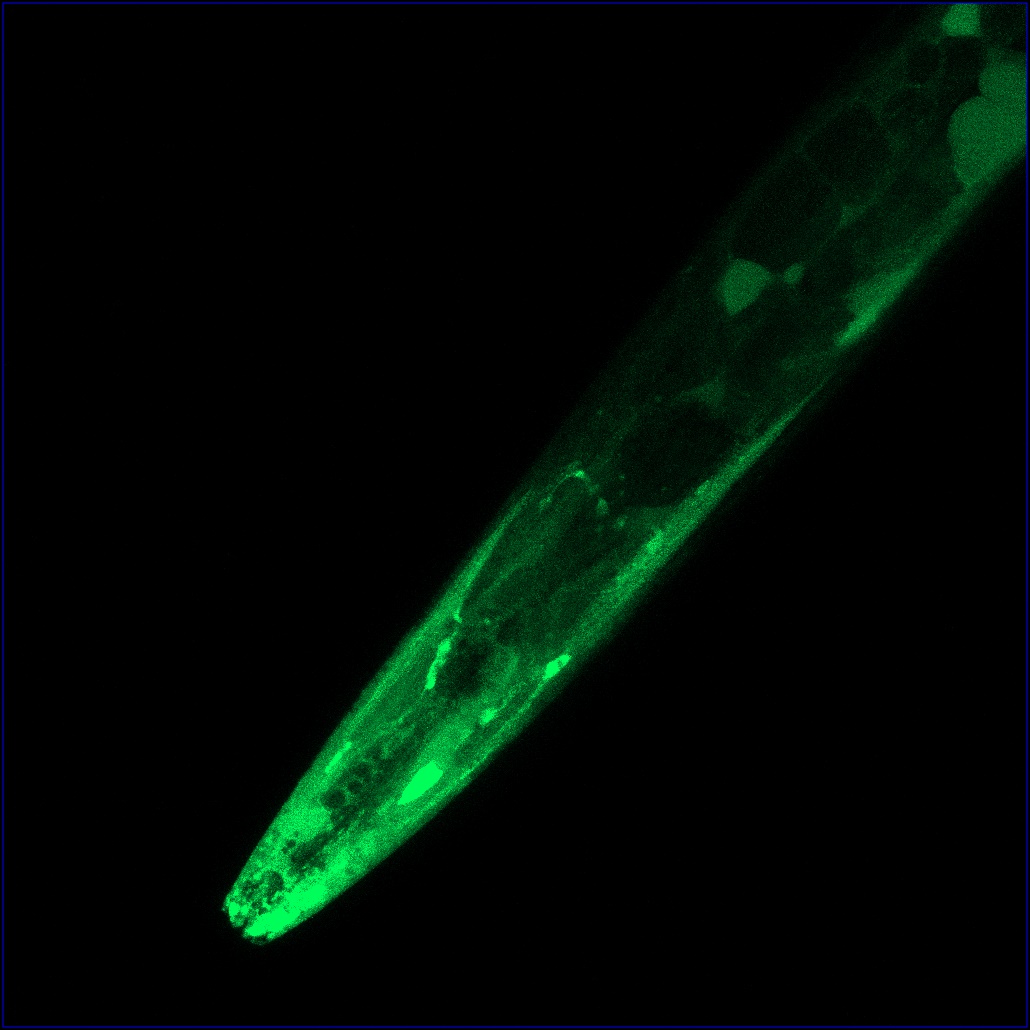

Supplement: Supplementary file 9 — Source data Fig. 5 [file 44319_2025_368_MOESM9_ESM.zip › Fig. 5 source data/5I/daf-18(D137A)+50 uM MG132.jpg]

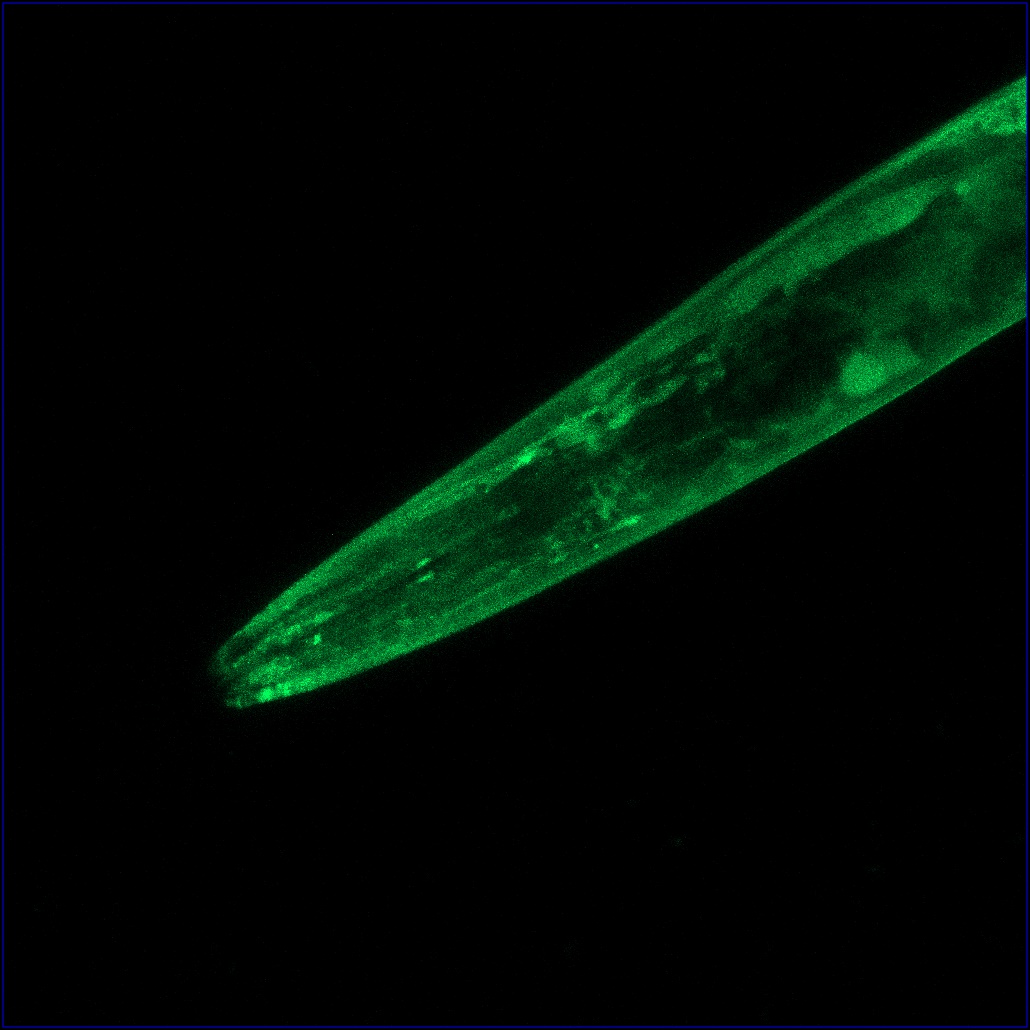

Supplement: Supplementary file 9 — Source data Fig. 5 [file 44319_2025_368_MOESM9_ESM.zip › Fig. 5 source data/5I/daf-18(D137A).jpg]

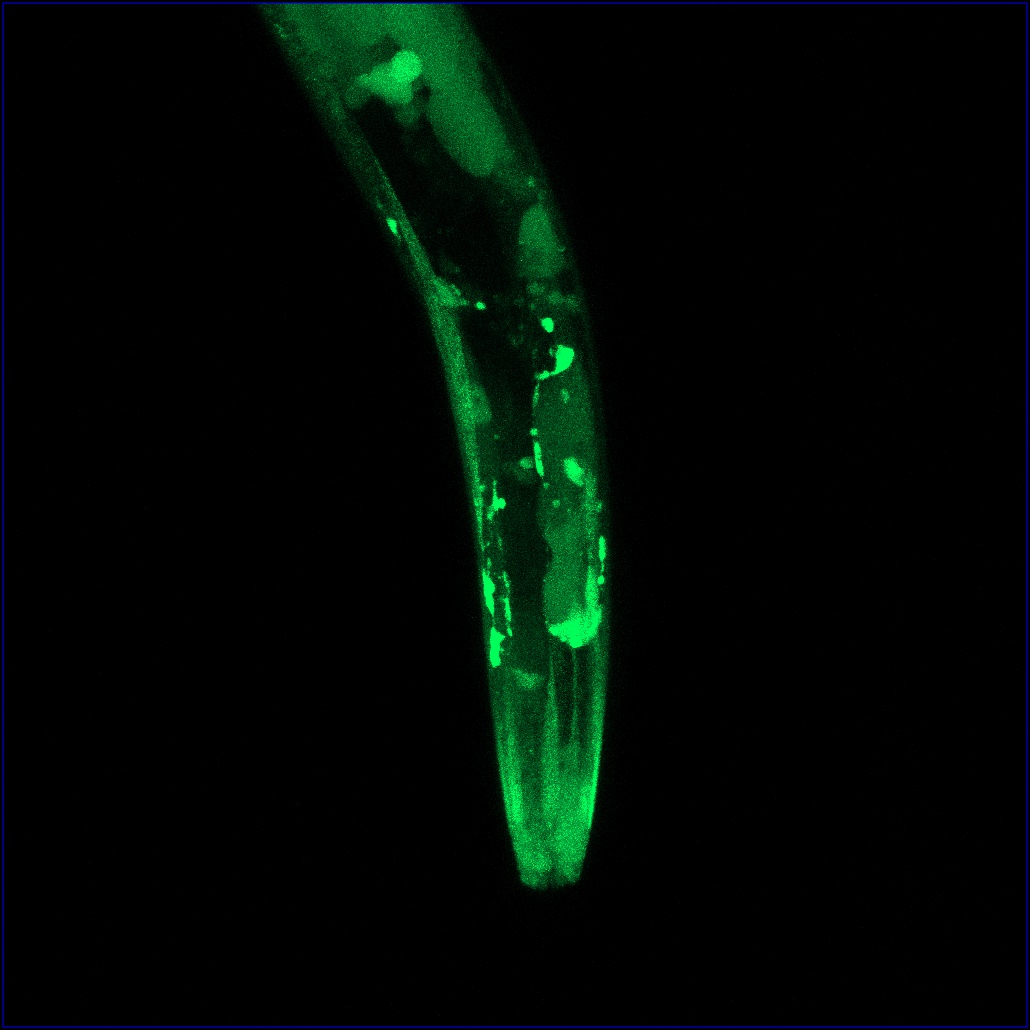

Supplement: Supplementary file 9 — Source data Fig. 5 [file 44319_2025_368_MOESM9_ESM.zip › Fig. 5 source data/5I/daf-18(D137A);unc-23 RNAi.jpg]

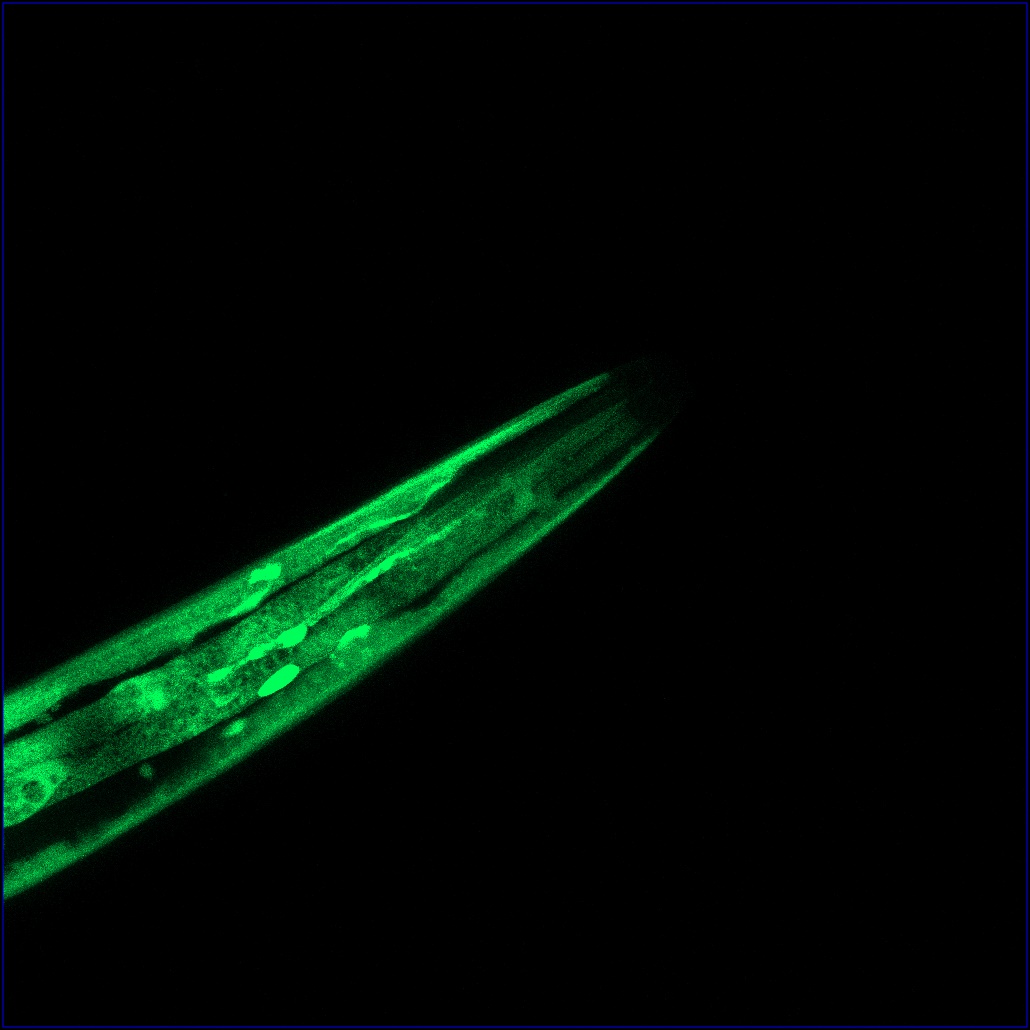

Supplement: Supplementary file 9 — Source data Fig. 5 [file 44319_2025_368_MOESM9_ESM.zip › Fig. 5 source data/5I/unc-23 RNAi.jpg]

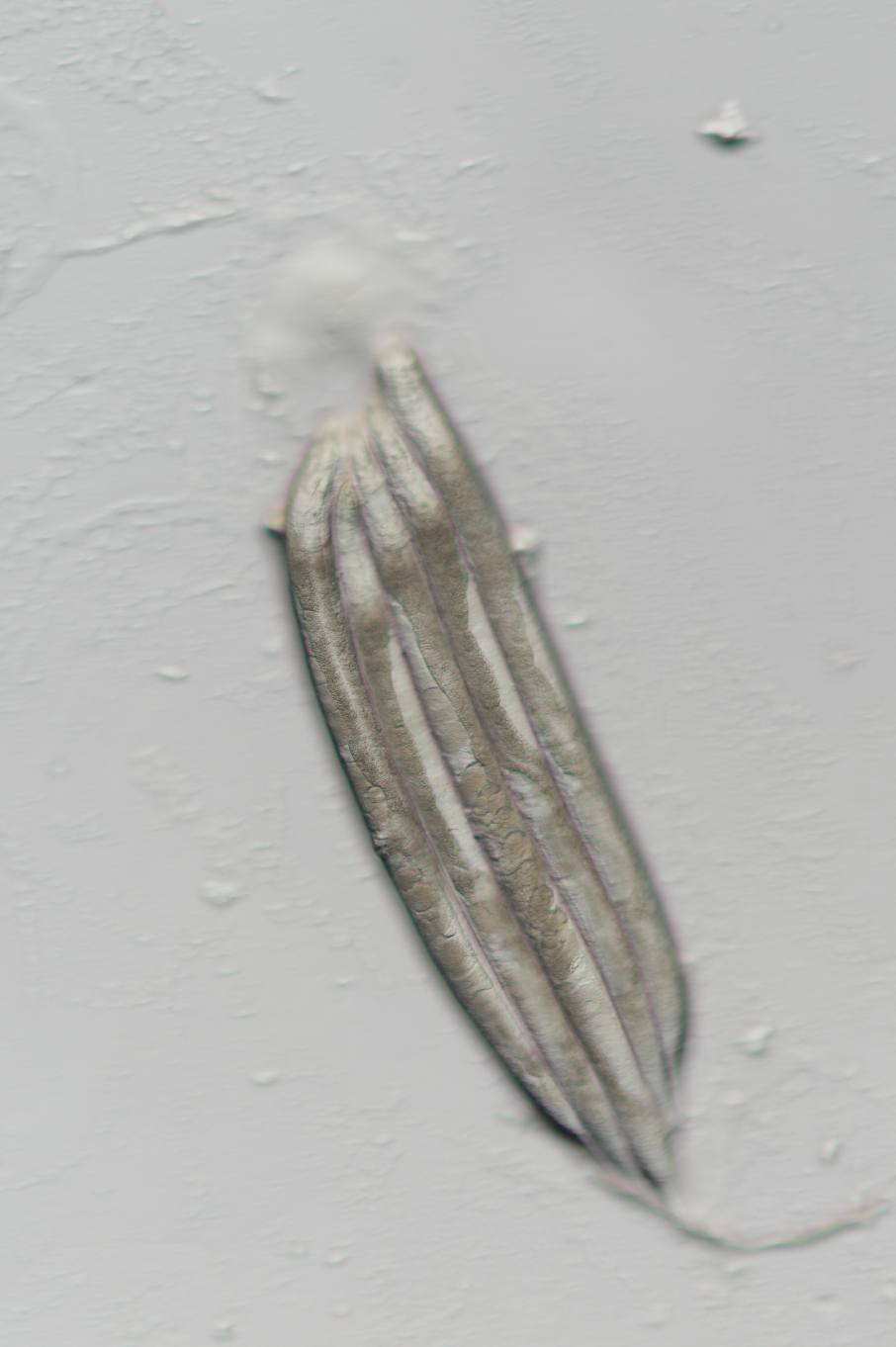

Supplement: Supplementary file 10 — Source data Fig. 6 [file 44319_2025_368_MOESM10_ESM.zip › Fig. 6 source data/6F/C18E9.2(D137A),.tif]

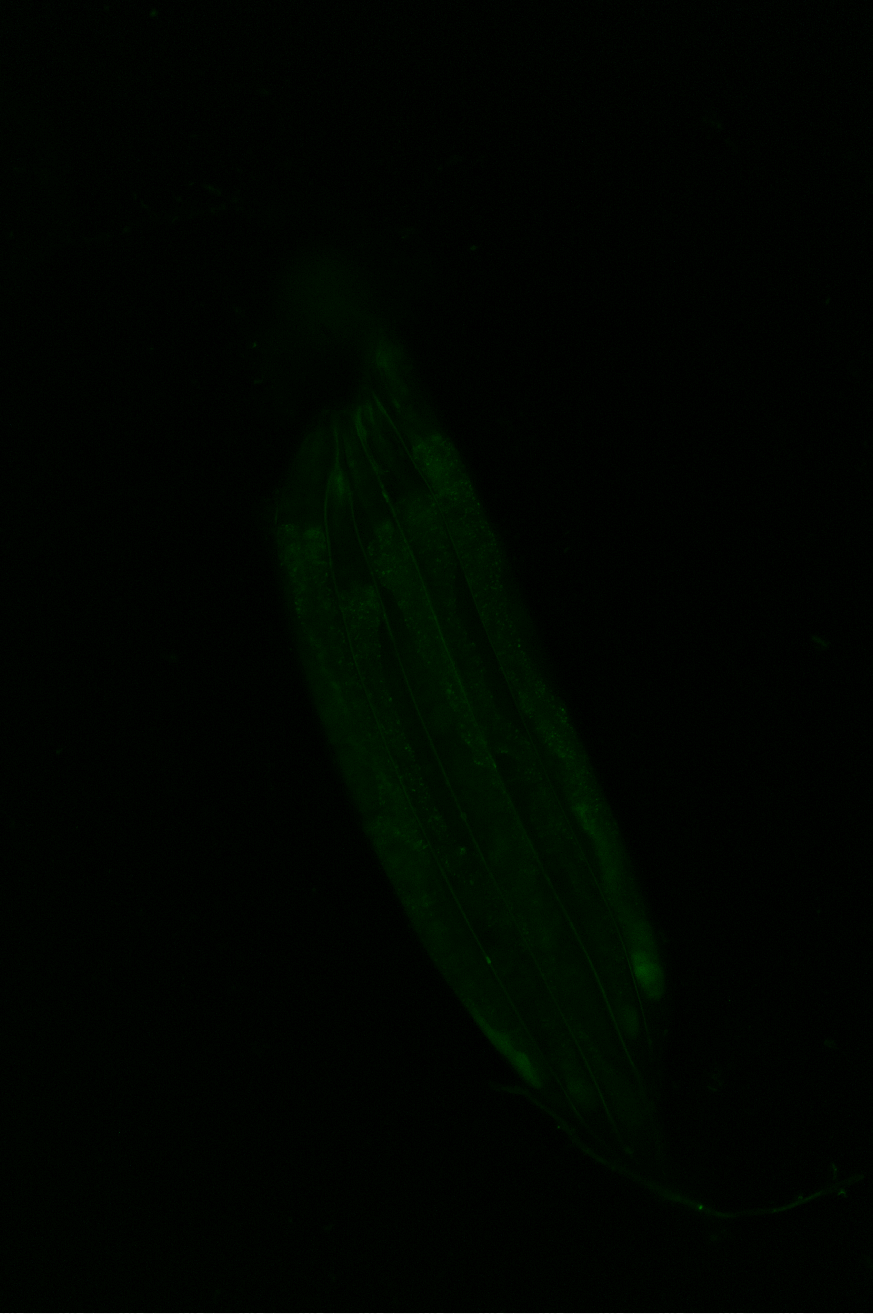

Supplement: Supplementary file 10 — Source data Fig. 6 [file 44319_2025_368_MOESM10_ESM.zip › Fig. 6 source data/6F/C18E9.2(D137A).tif]

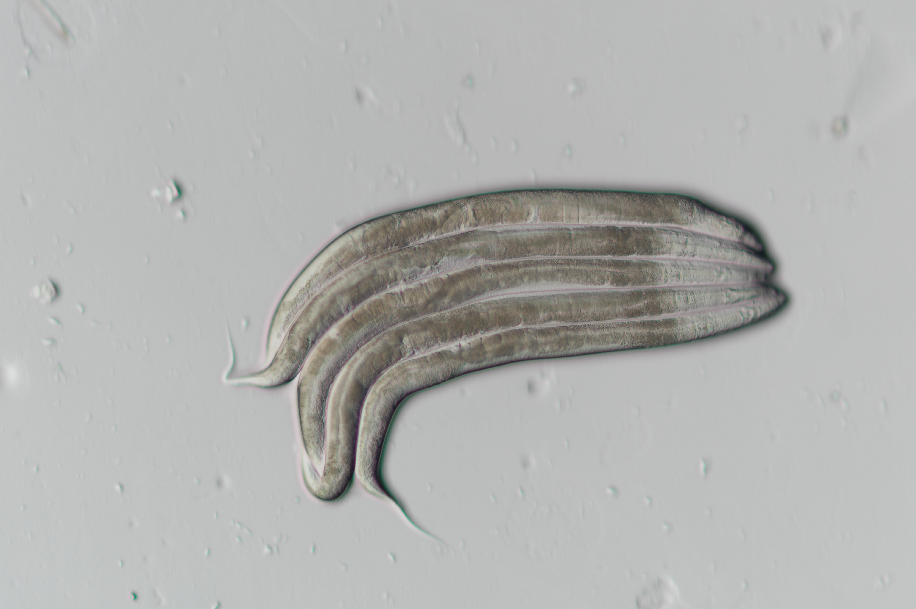

Supplement: Supplementary file 10 — Source data Fig. 6 [file 44319_2025_368_MOESM10_ESM.zip › Fig. 6 source data/6F/Control,.tif]

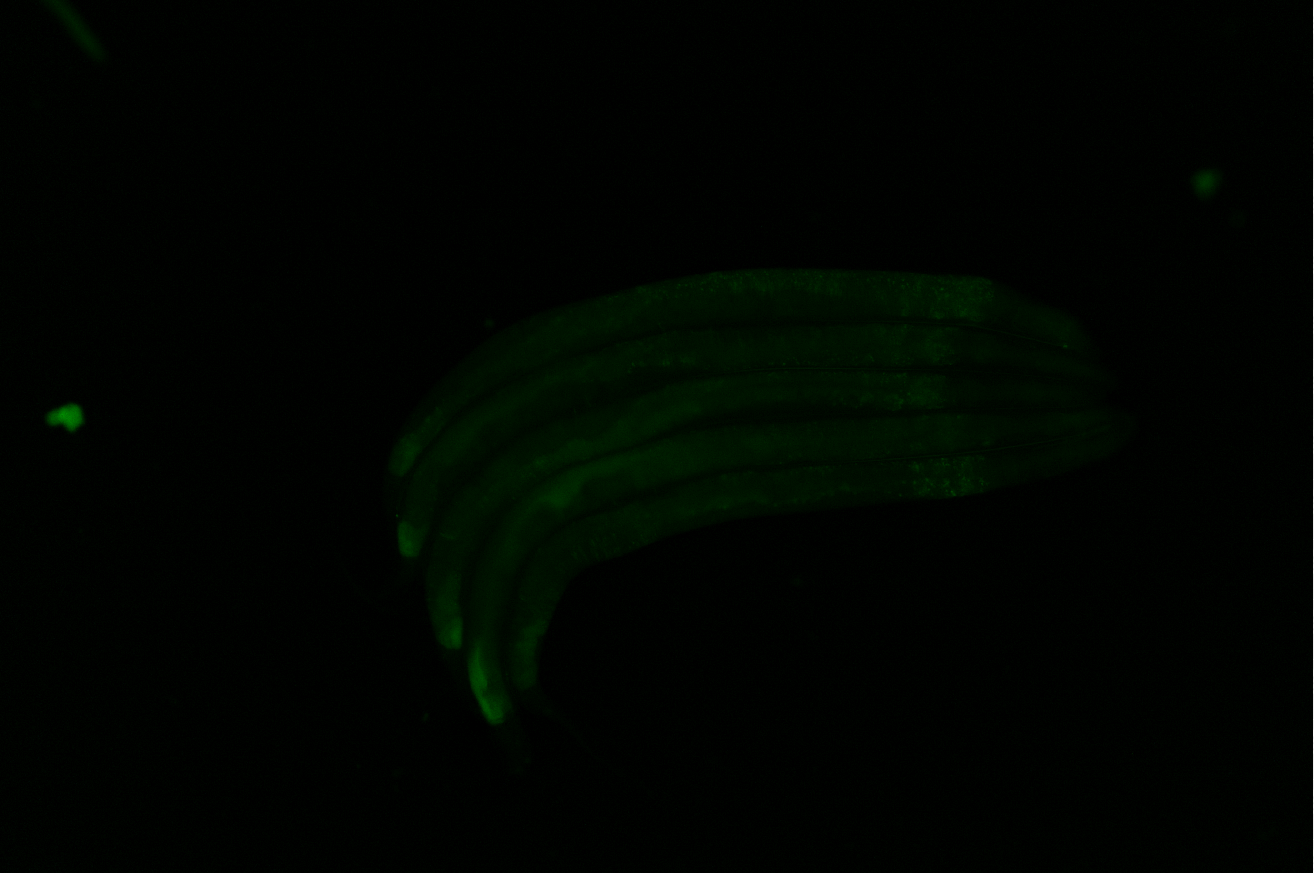

Supplement: Supplementary file 10 — Source data Fig. 6 [file 44319_2025_368_MOESM10_ESM.zip › Fig. 6 source data/6F/Control.tif]

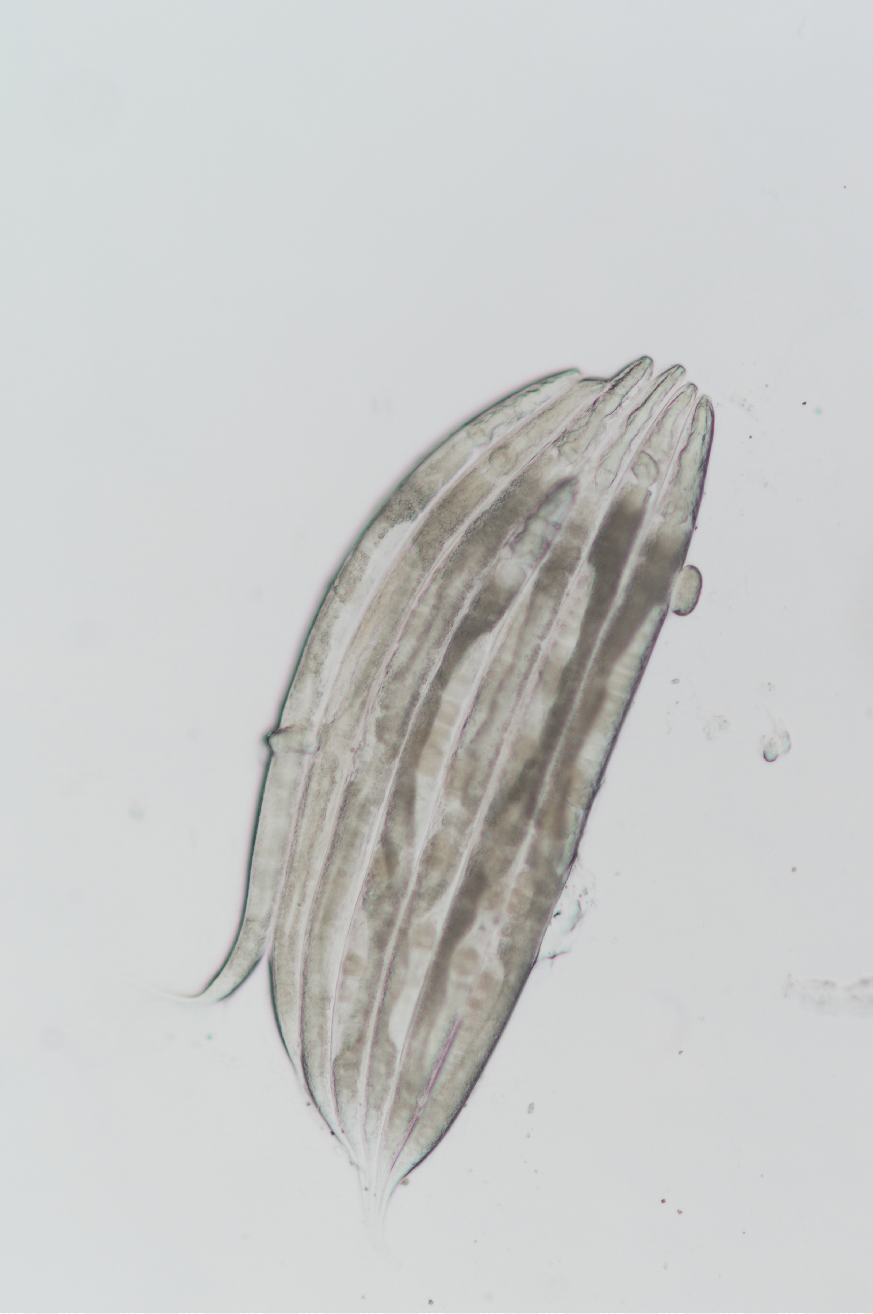

Supplement: Supplementary file 10 — Source data Fig. 6 [file 44319_2025_368_MOESM10_ESM.zip › Fig. 6 source data/6F/daf-18(D137A),.tif]

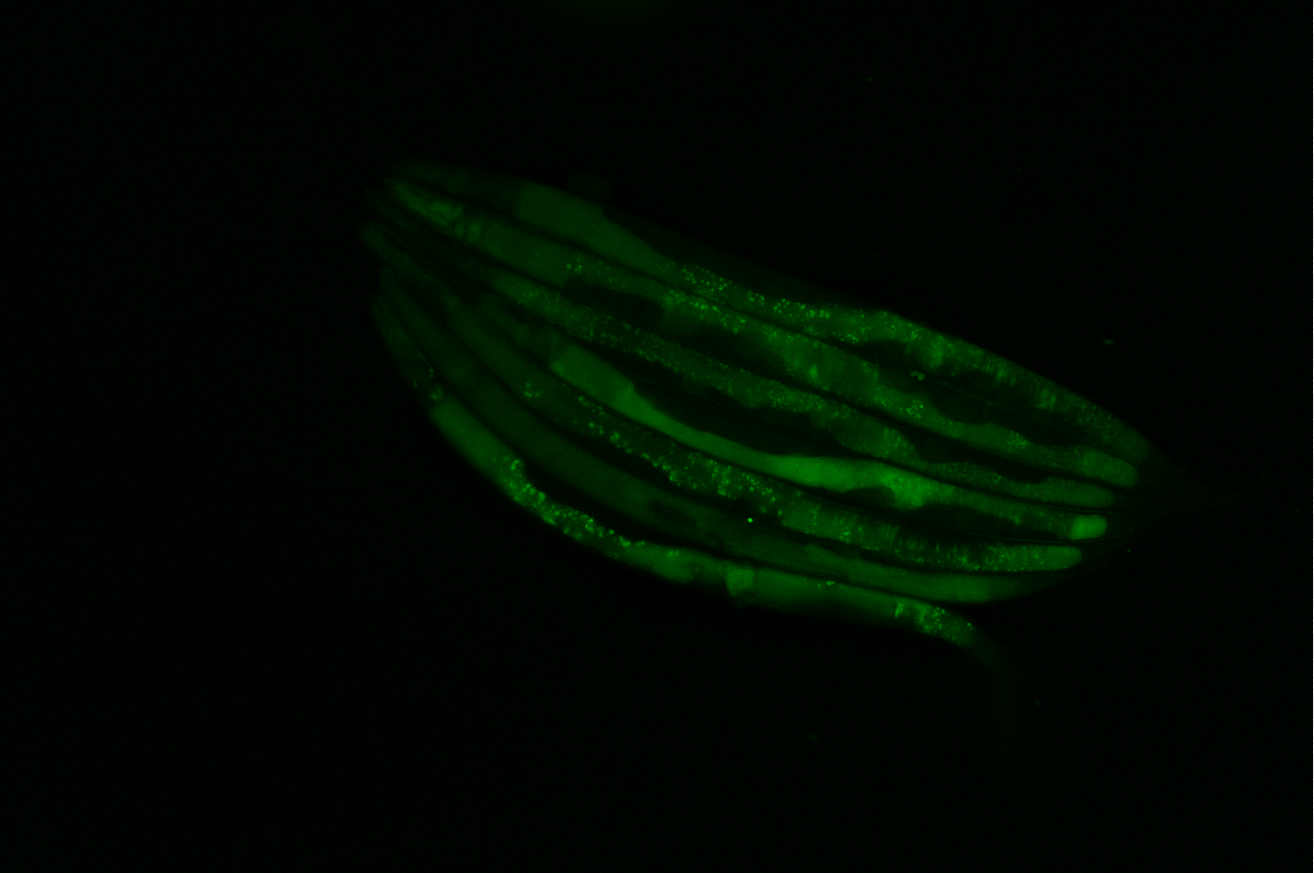

Supplement: Supplementary file 10 — Source data Fig. 6 [file 44319_2025_368_MOESM10_ESM.zip › Fig. 6 source data/6F/daf-18(D137A).tif]

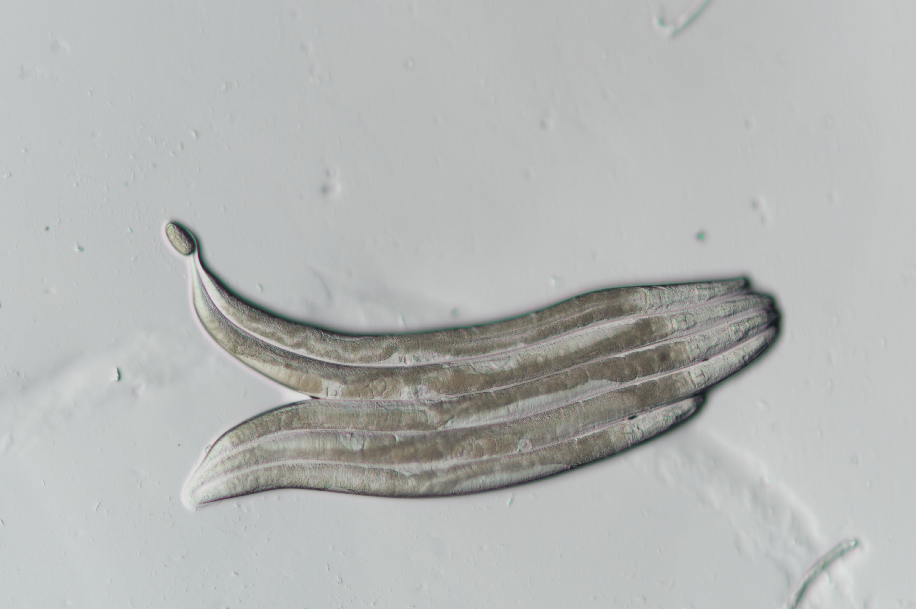

Supplement: Supplementary file 10 — Source data Fig. 6 [file 44319_2025_368_MOESM10_ESM.zip › Fig. 6 source data/6F/daf-18(D137A);C18E9.2(S301A),.tif]

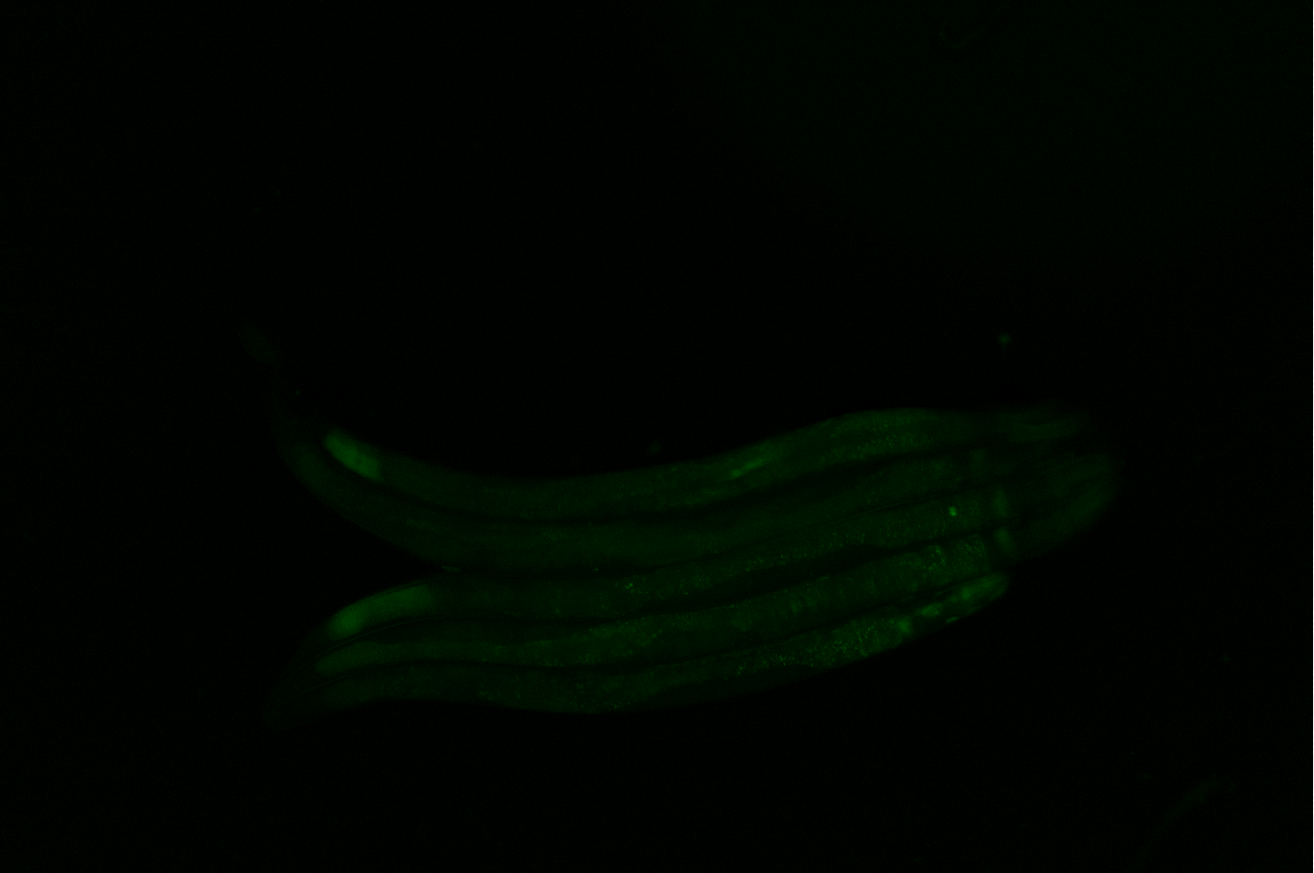

Supplement: Supplementary file 10 — Source data Fig. 6 [file 44319_2025_368_MOESM10_ESM.zip › Fig. 6 source data/6F/daf-18(D137A);C18E9.2(S301A).tif]

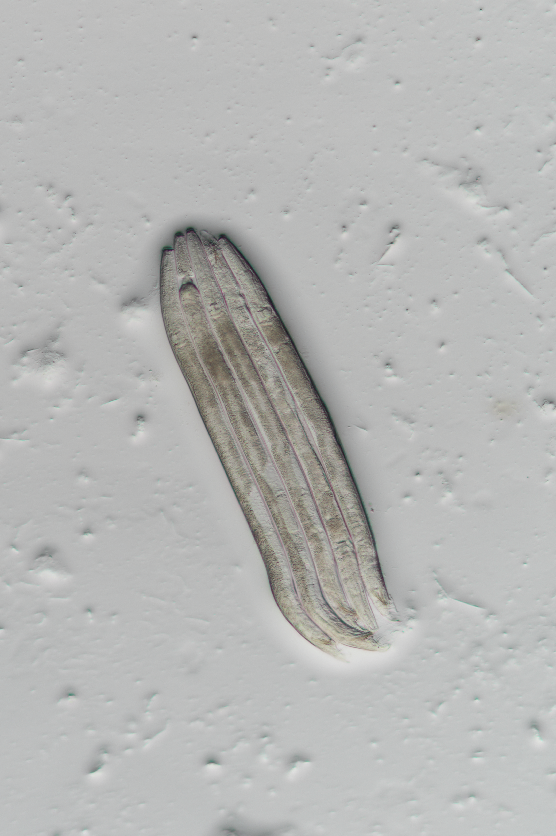

Supplement: Supplementary file 10 — Source data Fig. 6 [file 44319_2025_368_MOESM10_ESM.zip › Fig. 6 source data/6G/C18E9.2(S301E),.tif]

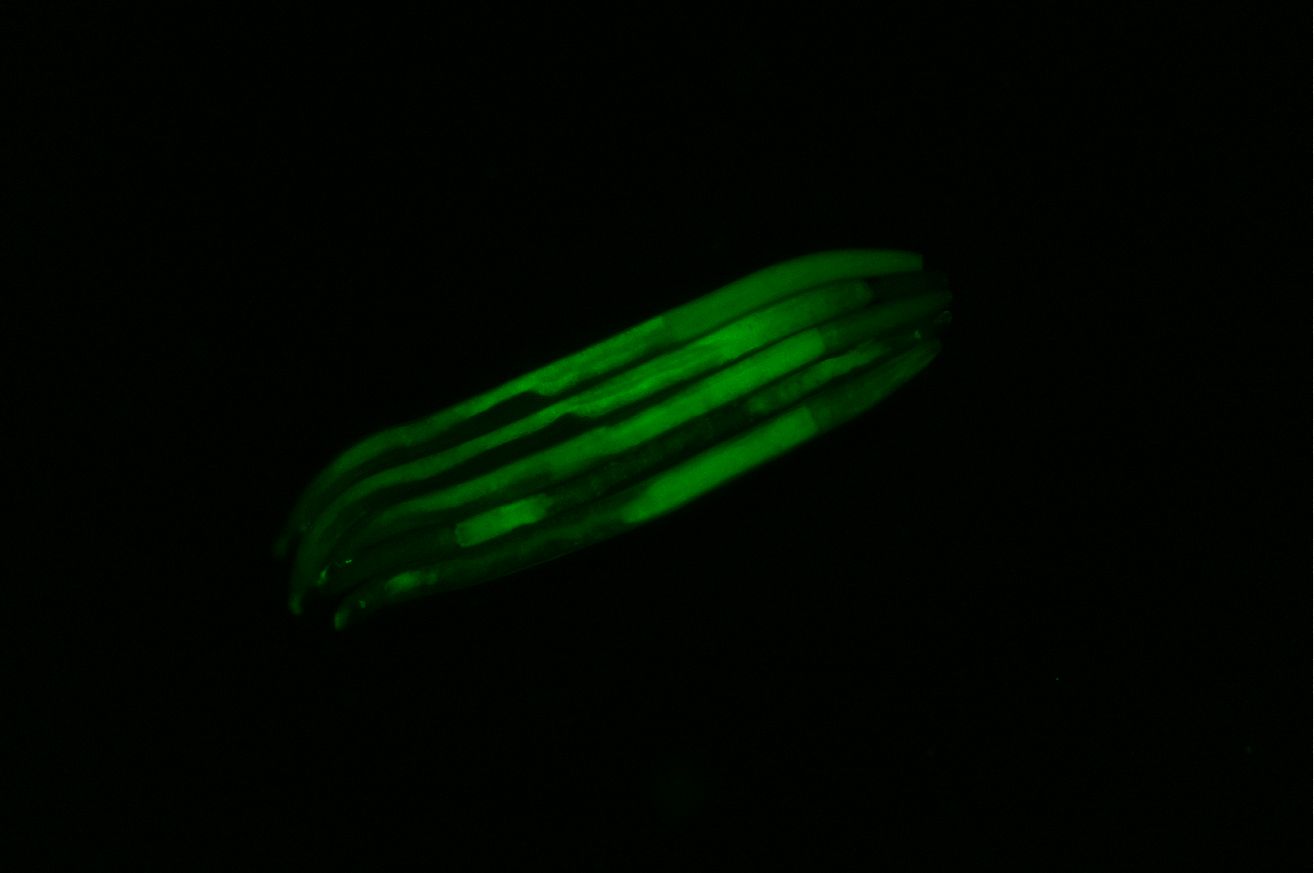

Supplement: Supplementary file 10 — Source data Fig. 6 [file 44319_2025_368_MOESM10_ESM.zip › Fig. 6 source data/6G/C18E9.2(S301E).tif]

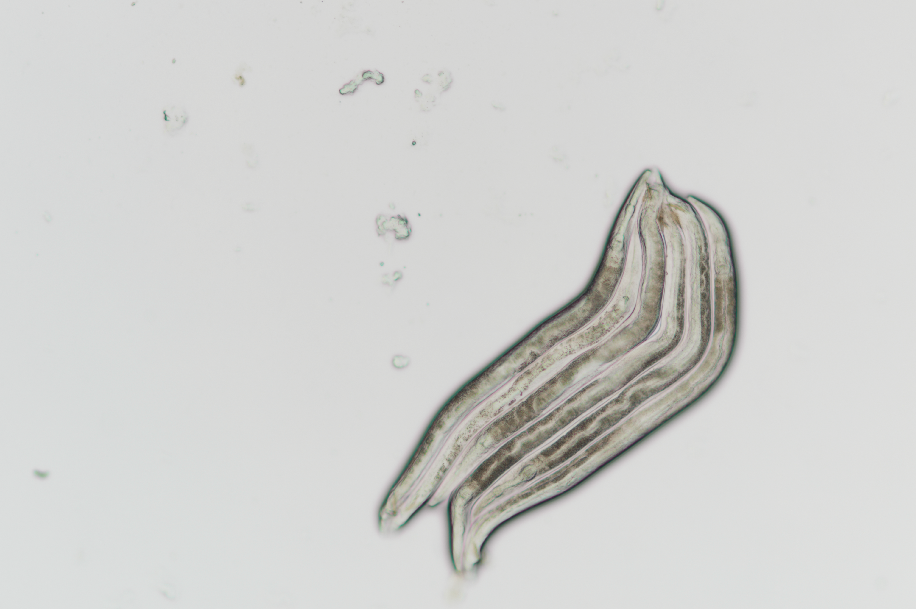

Supplement: Supplementary file 10 — Source data Fig. 6 [file 44319_2025_368_MOESM10_ESM.zip › Fig. 6 source data/6G/Control,.tif]

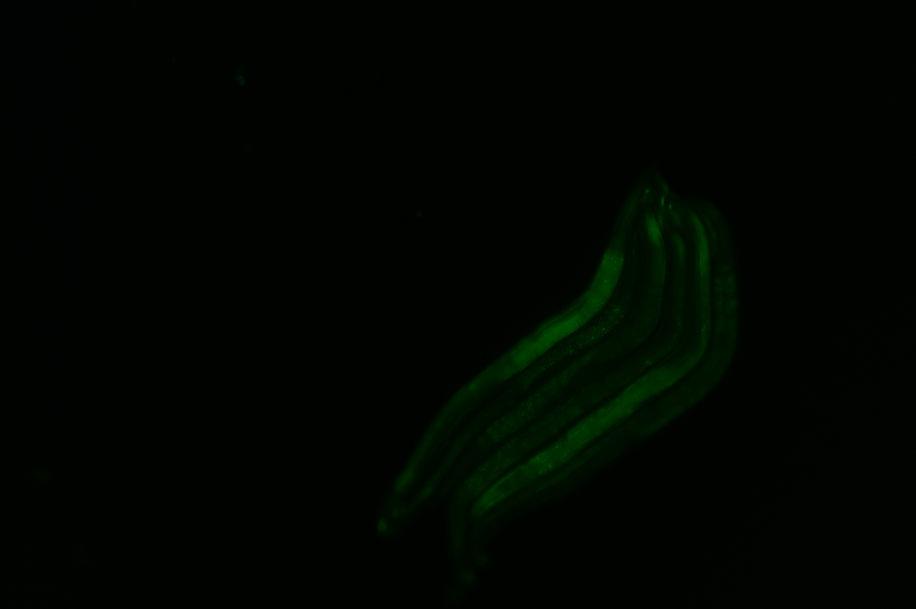

Supplement: Supplementary file 10 — Source data Fig. 6 [file 44319_2025_368_MOESM10_ESM.zip › Fig. 6 source data/6G/Control.tif]

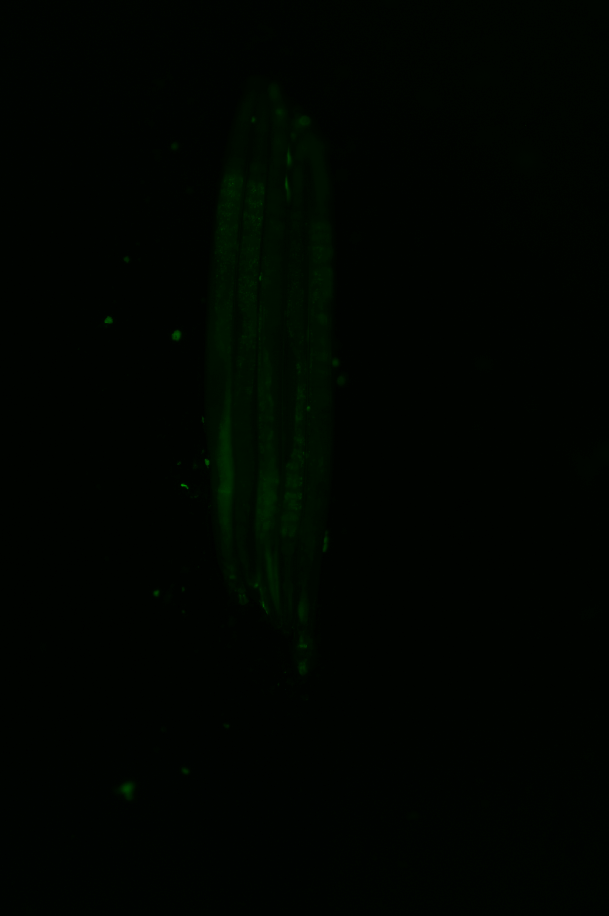

Supplement: Supplementary file 10 — Source data Fig. 6 [file 44319_2025_368_MOESM10_ESM.zip › Fig. 6 source data/6G/daf-18(D137A),.tif]

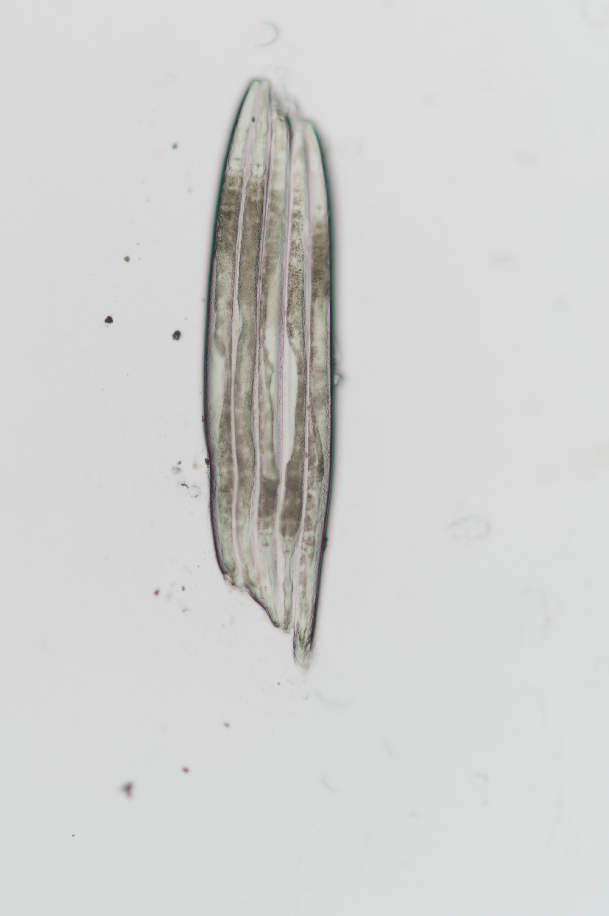

Supplement: Supplementary file 10 — Source data Fig. 6 [file 44319_2025_368_MOESM10_ESM.zip › Fig. 6 source data/6G/daf-18(D137A).tif]

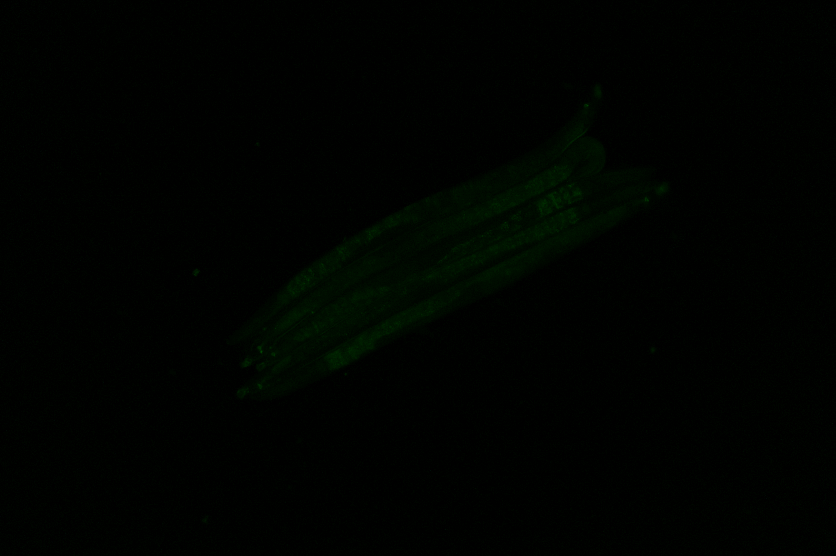

Supplement: Supplementary file 10 — Source data Fig. 6 [file 44319_2025_368_MOESM10_ESM.zip › Fig. 6 source data/6G/daf-18(D137A);C18E9.2(S301E),.tif]

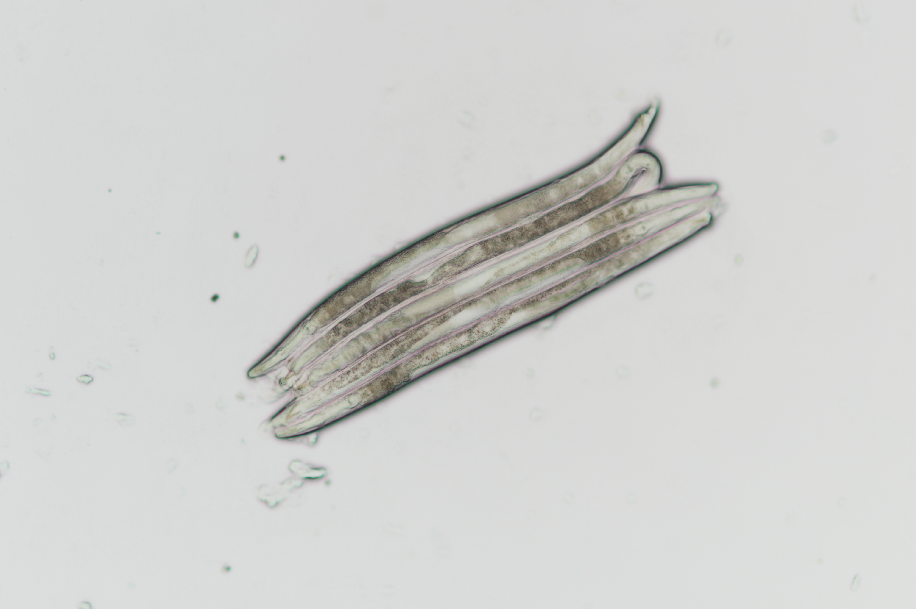

Supplement: Supplementary file 10 — Source data Fig. 6 [file 44319_2025_368_MOESM10_ESM.zip › Fig. 6 source data/6G/daf-18(D137A);C18E9.2(S301E).tif]

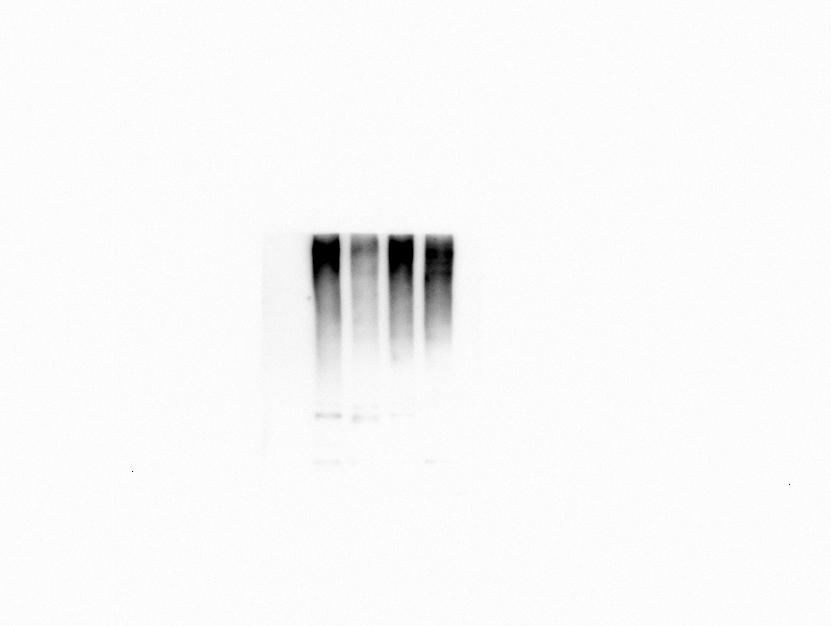

Supplement: Supplementary file 10 — Source data Fig. 6 [file 44319_2025_368_MOESM10_ESM.zip › Fig. 6 source data/6J/Fig.6J-k48.png]

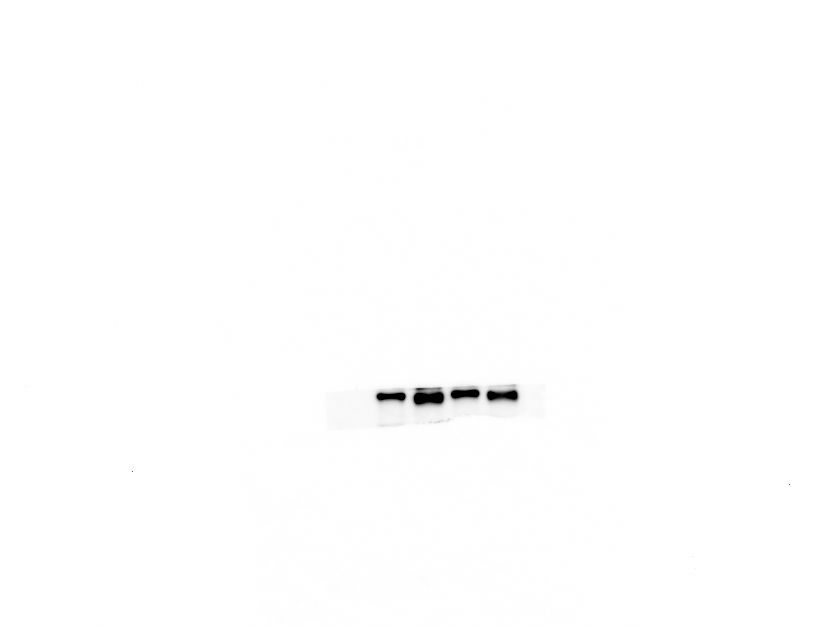

Supplement: Supplementary file 10 — Source data Fig. 6 [file 44319_2025_368_MOESM10_ESM.zip › Fig. 6 source data/6J/Fig.6J-tubulin.png]

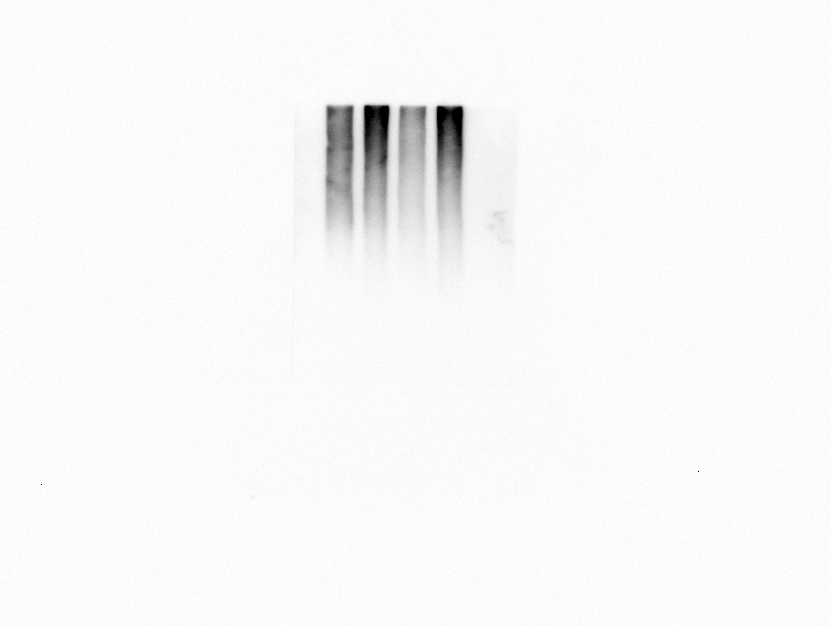

Supplement: Supplementary file 10 — Source data Fig. 6 [file 44319_2025_368_MOESM10_ESM.zip › Fig. 6 source data/6K/Fig.6K-K48.png]

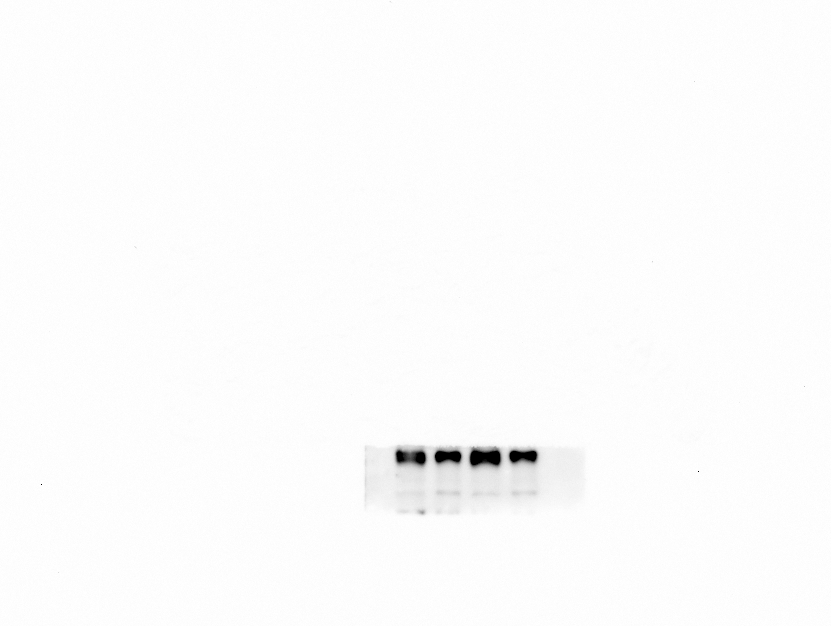

Supplement: Supplementary file 10 — Source data Fig. 6 [file 44319_2025_368_MOESM10_ESM.zip › Fig. 6 source data/6K/Fig.6K-tubulin.png]

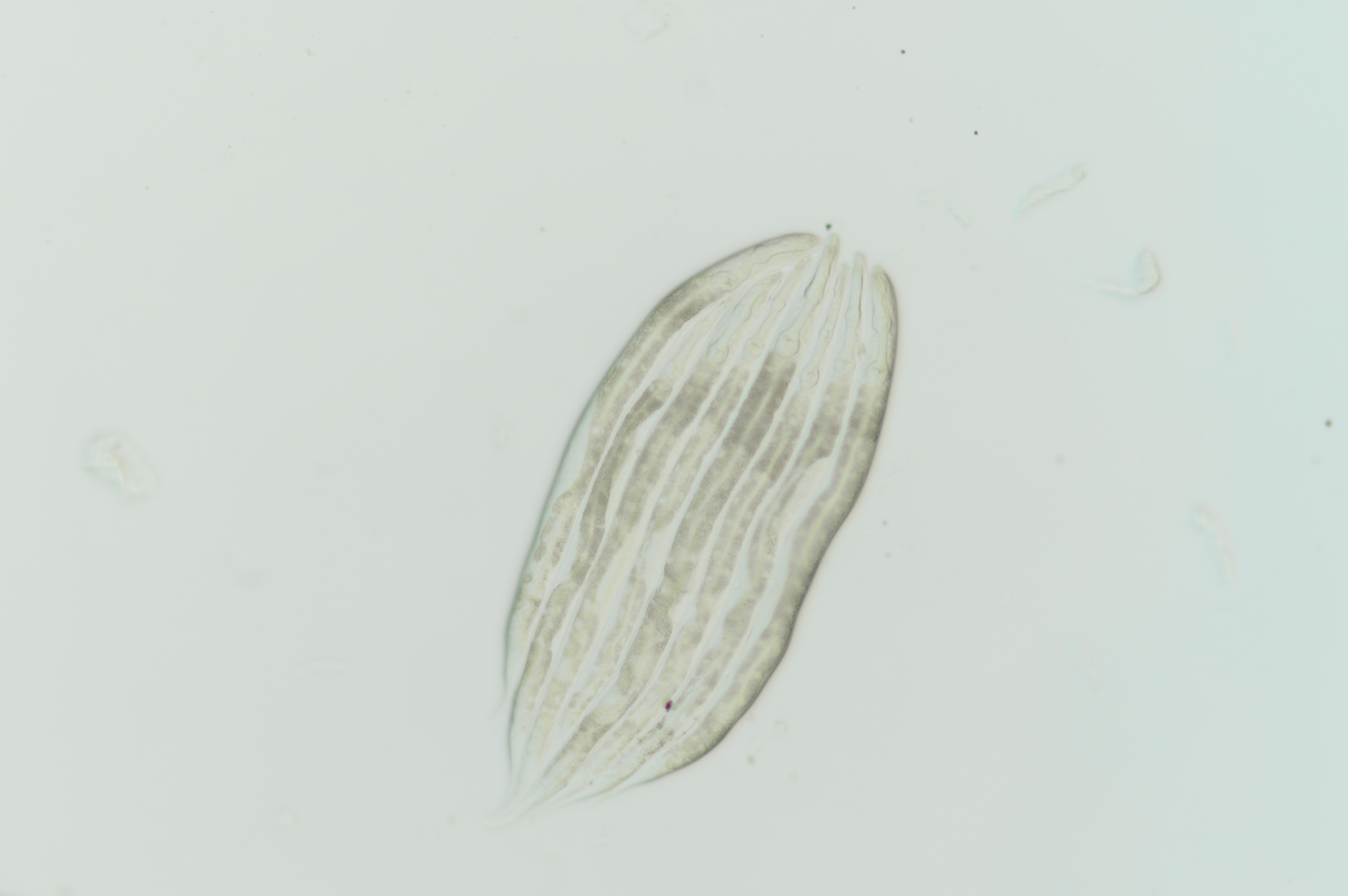

Supplement: Supplementary file 11 — Source data Fig. 7 [file 44319_2025_368_MOESM11_ESM.zip › Fig. 7 source data/7L/daf-18(ok480);him-5(e1490);control,.tif]

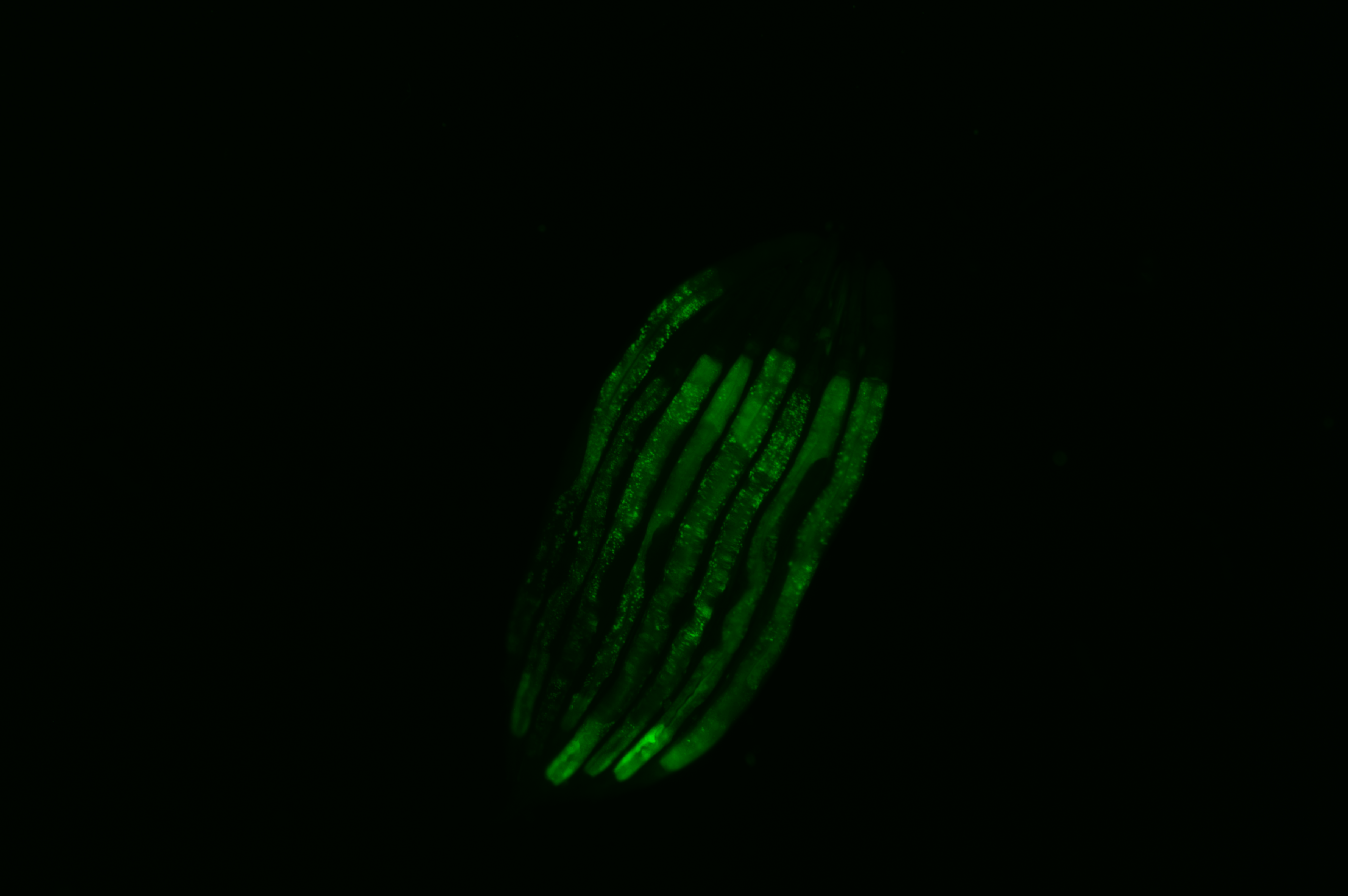

Supplement: Supplementary file 11 — Source data Fig. 7 [file 44319_2025_368_MOESM11_ESM.zip › Fig. 7 source data/7L/daf-18(ok480);him-5(e1490);control.tif]

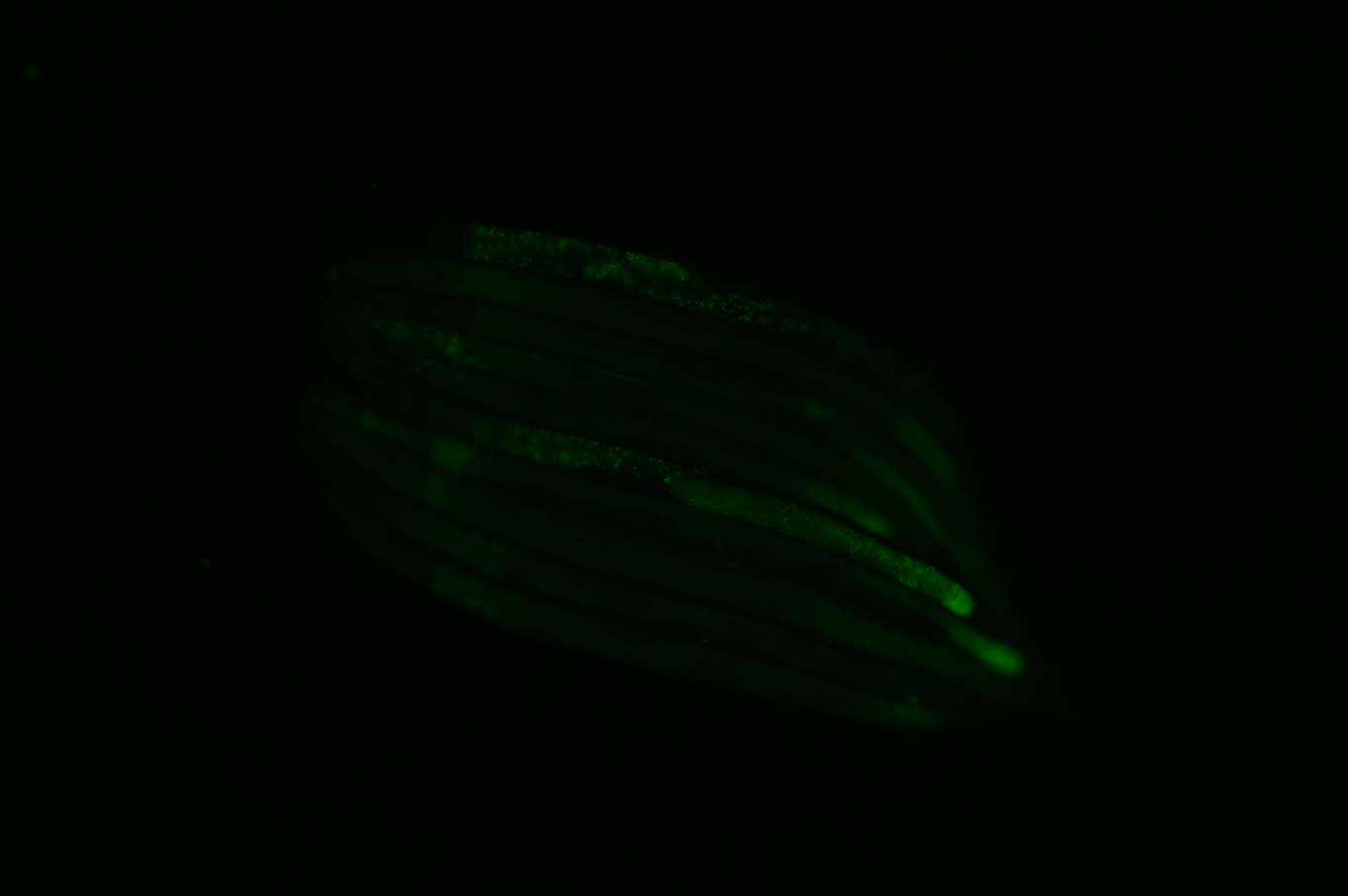

Supplement: Supplementary file 11 — Source data Fig. 7 [file 44319_2025_368_MOESM11_ESM.zip › Fig. 7 source data/7L/daf-18(ok480);him-5(e1490);tra-3 RNAi,.tif]

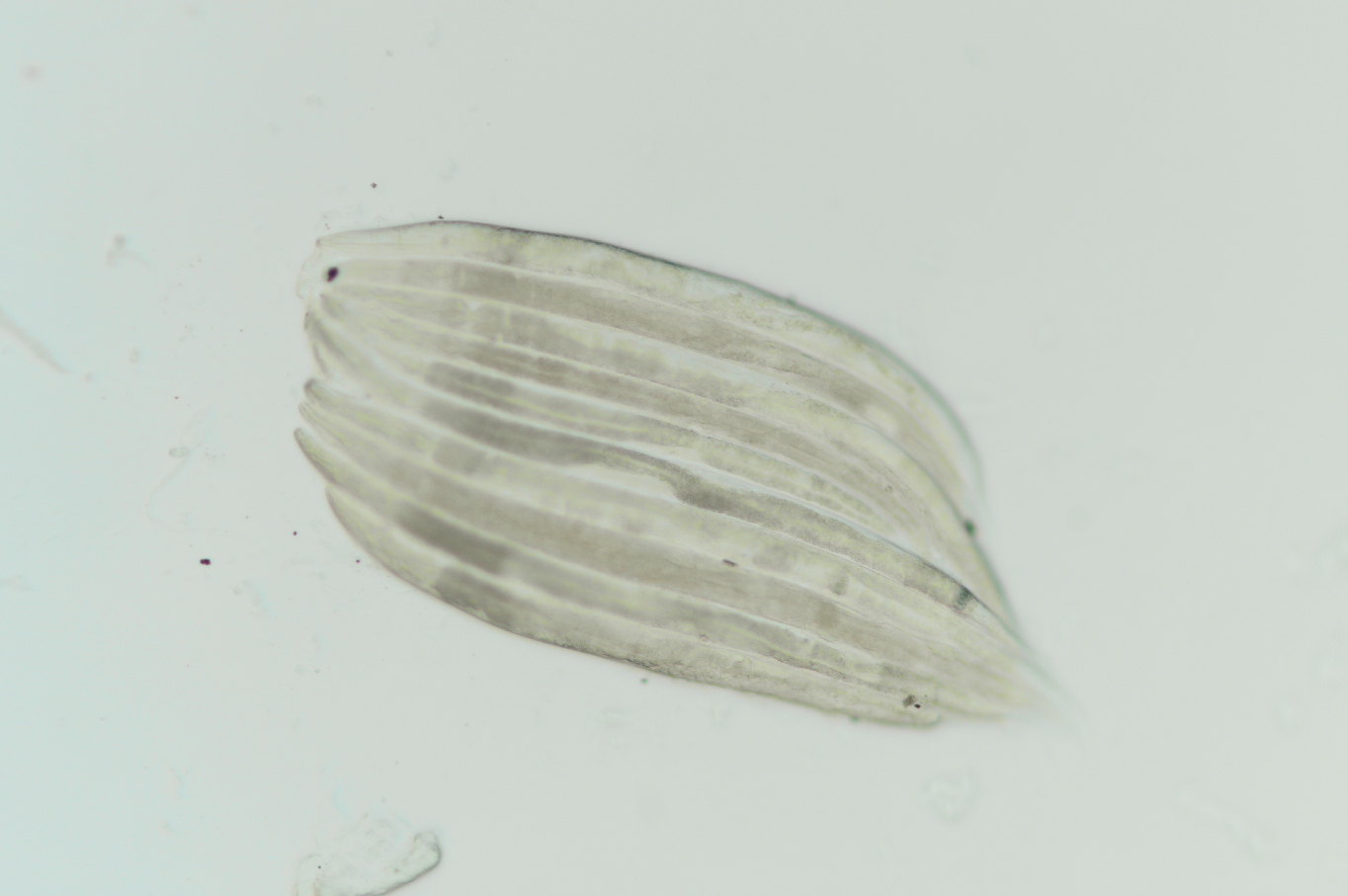

Supplement: Supplementary file 11 — Source data Fig. 7 [file 44319_2025_368_MOESM11_ESM.zip › Fig. 7 source data/7L/daf-18(ok480);him-5(e1490);tra-3 RNAi.tif]

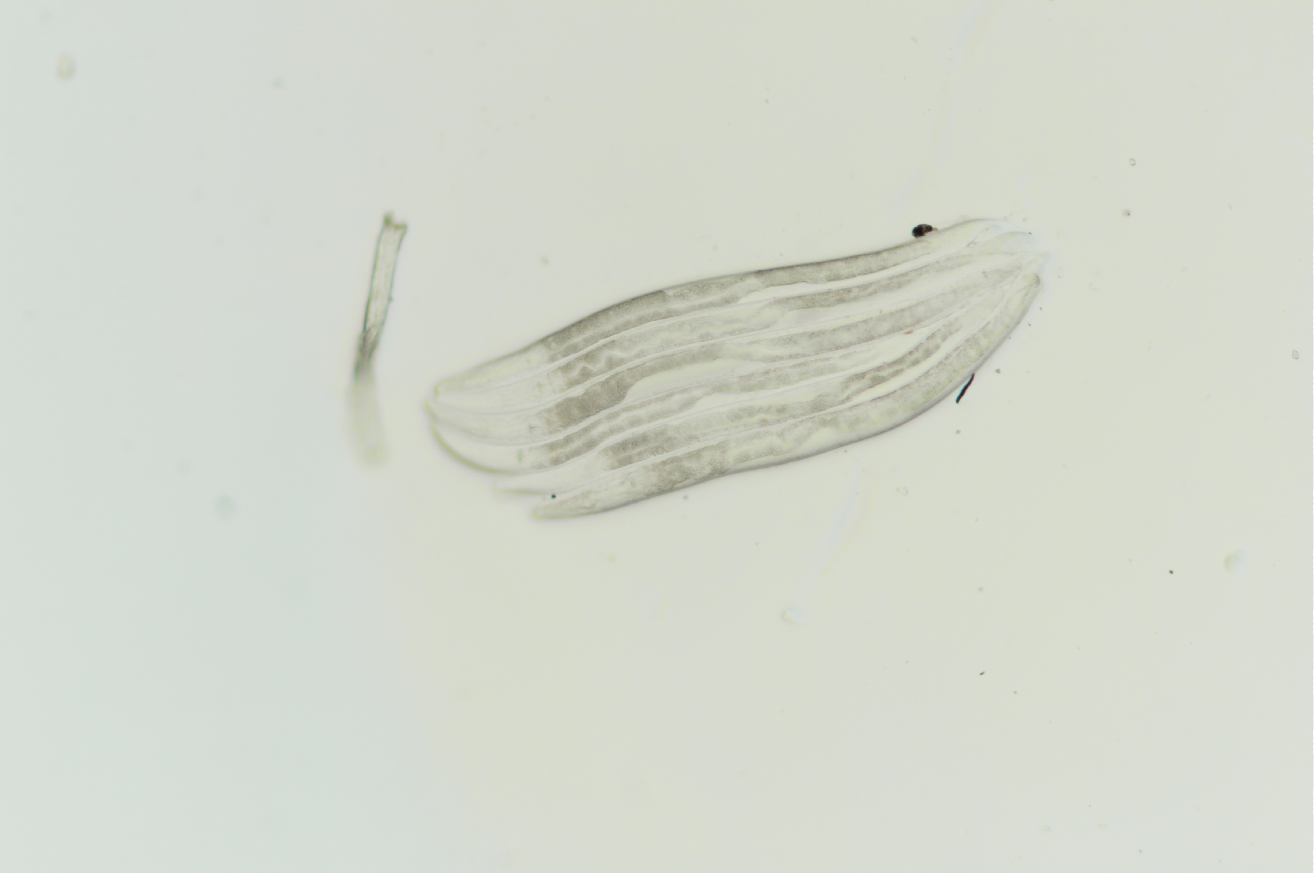

Supplement: Supplementary file 11 — Source data Fig. 7 [file 44319_2025_368_MOESM11_ESM.zip › Fig. 7 source data/7N/daf-18(ok480);him-5(e1490);control.tif]

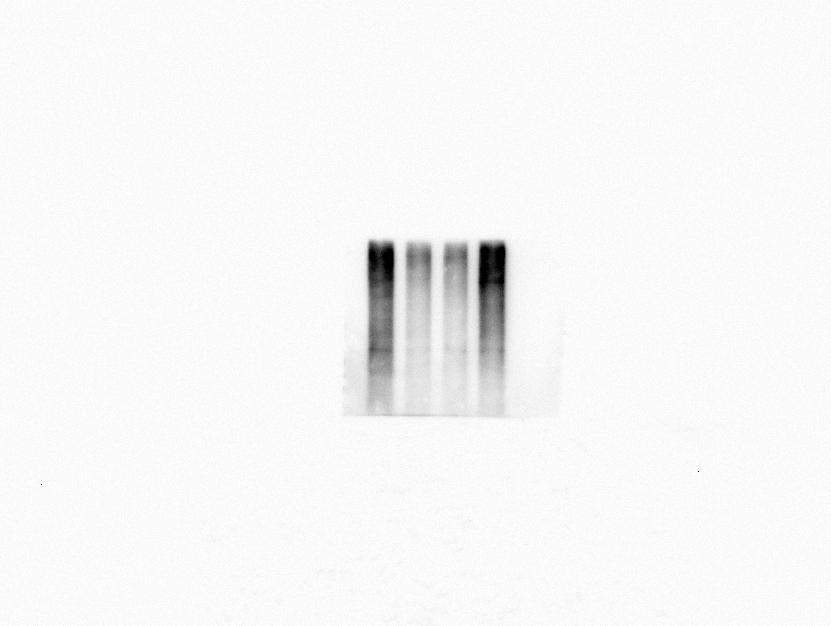

Supplement: Supplementary file 11 — Source data Fig. 7 [file 44319_2025_368_MOESM11_ESM.zip › Fig. 7 source data/7P/Fig.7P-k48.png]

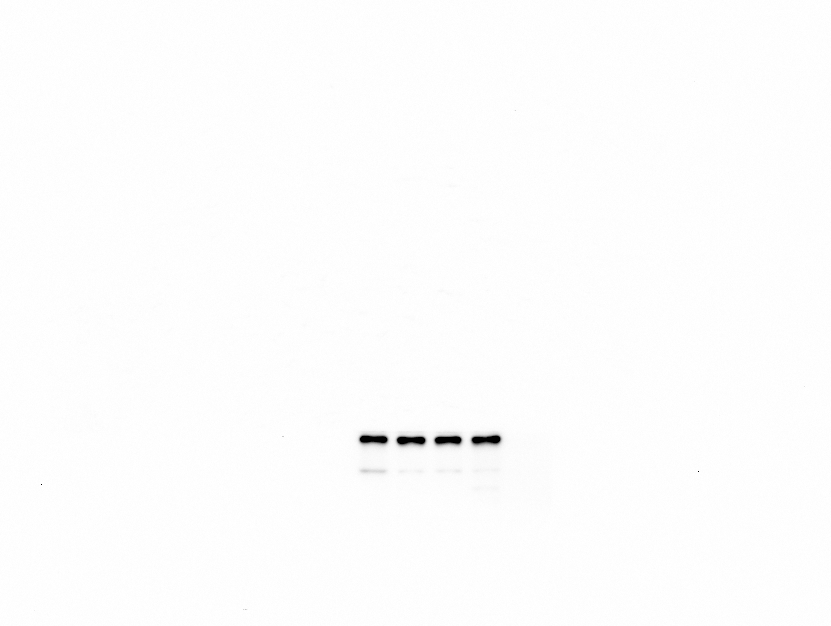

Supplement: Supplementary file 11 — Source data Fig. 7 [file 44319_2025_368_MOESM11_ESM.zip › Fig. 7 source data/7P/Fig.7P-tubulin.png]

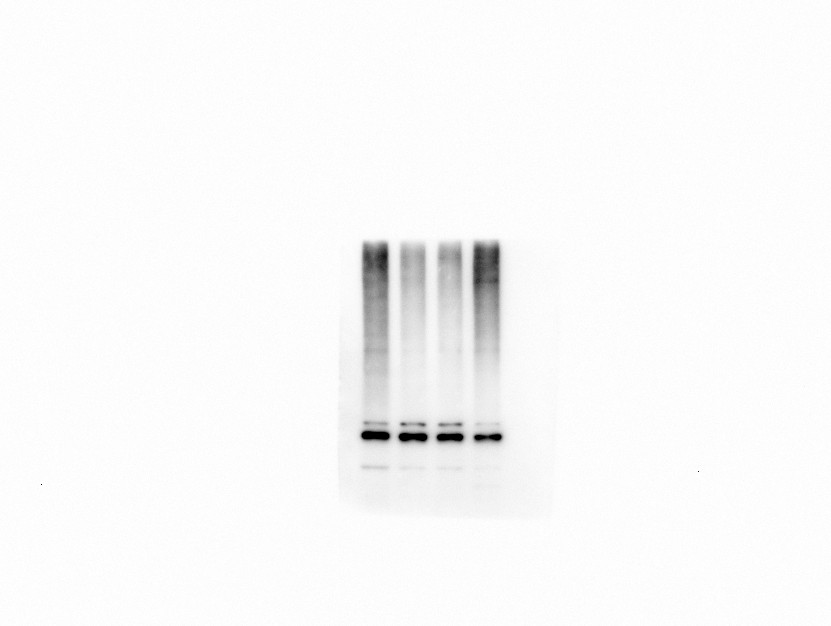

Supplement: Supplementary file 11 — Source data Fig. 7 [file 44319_2025_368_MOESM11_ESM.zip › Fig. 7 source data/7P/Fig.7P.png]

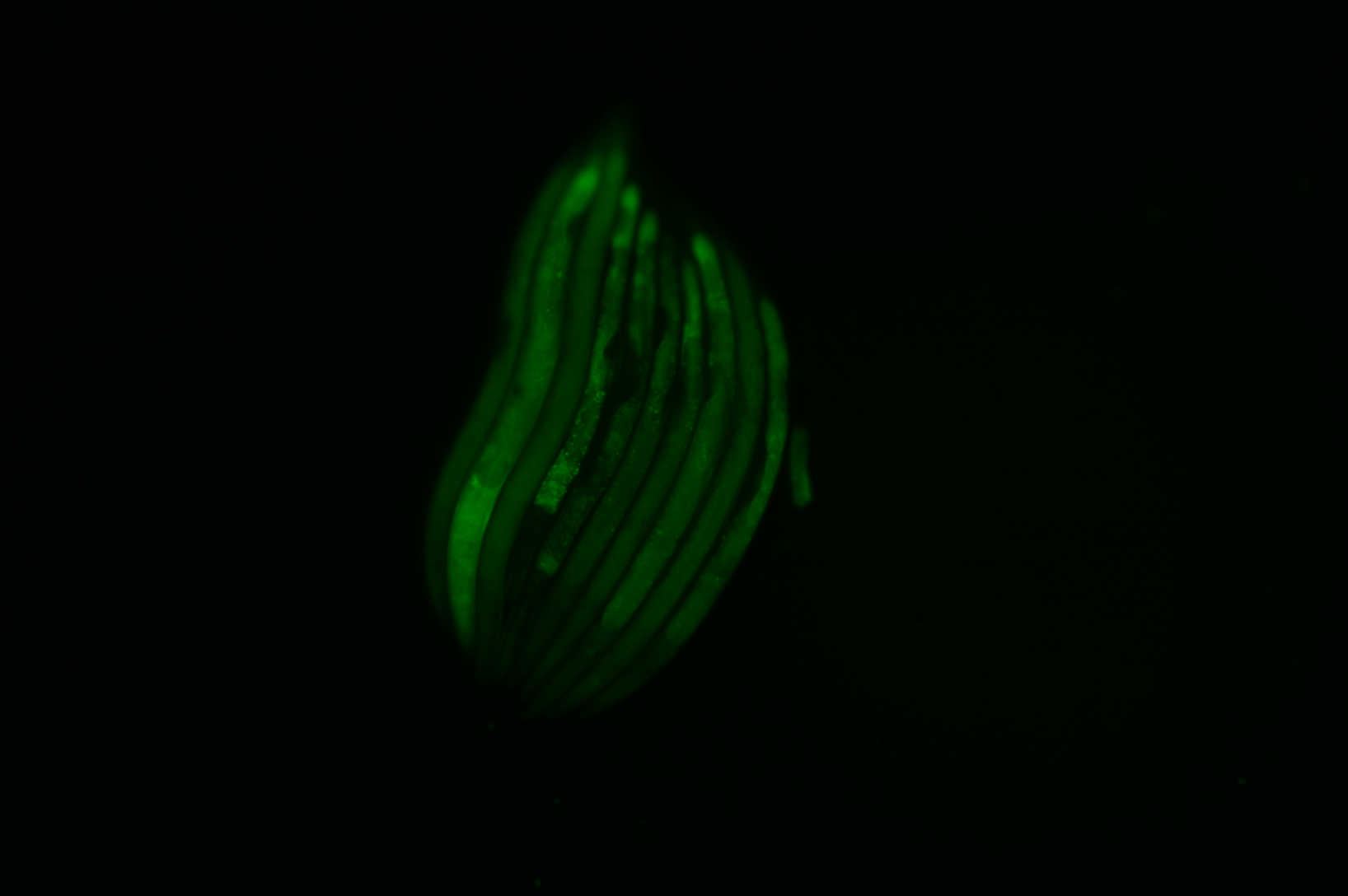

Supplement: Supplementary file 12 — Source data Fig. 8 [file 44319_2025_368_MOESM12_ESM.zip › Fig. 8 source data/8C/daf-18(ok480); unc-23(e25);control,.tif]

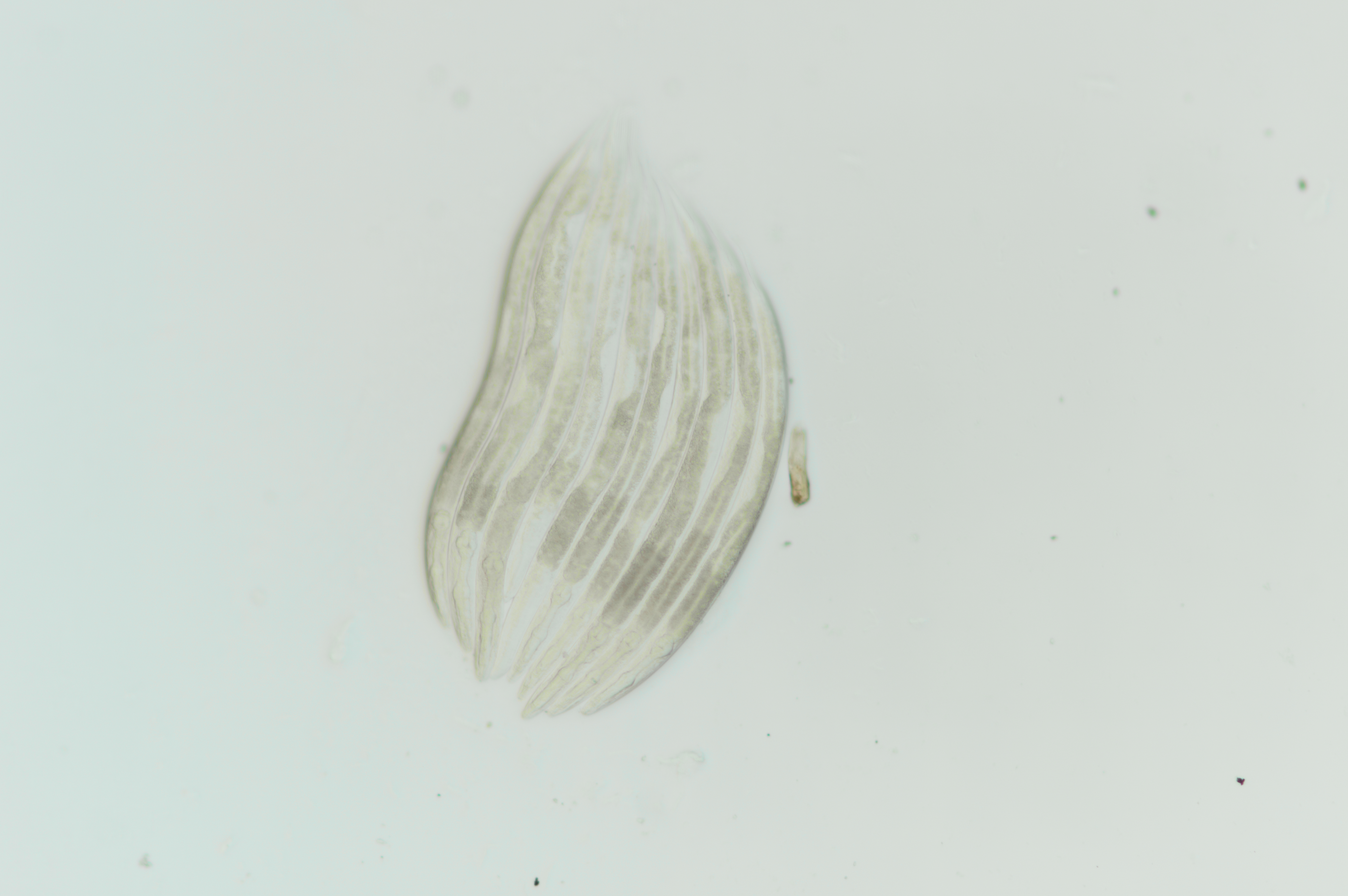

Supplement: Supplementary file 12 — Source data Fig. 8 [file 44319_2025_368_MOESM12_ESM.zip › Fig. 8 source data/8C/daf-18(ok480); unc-23(e25);control.tif]

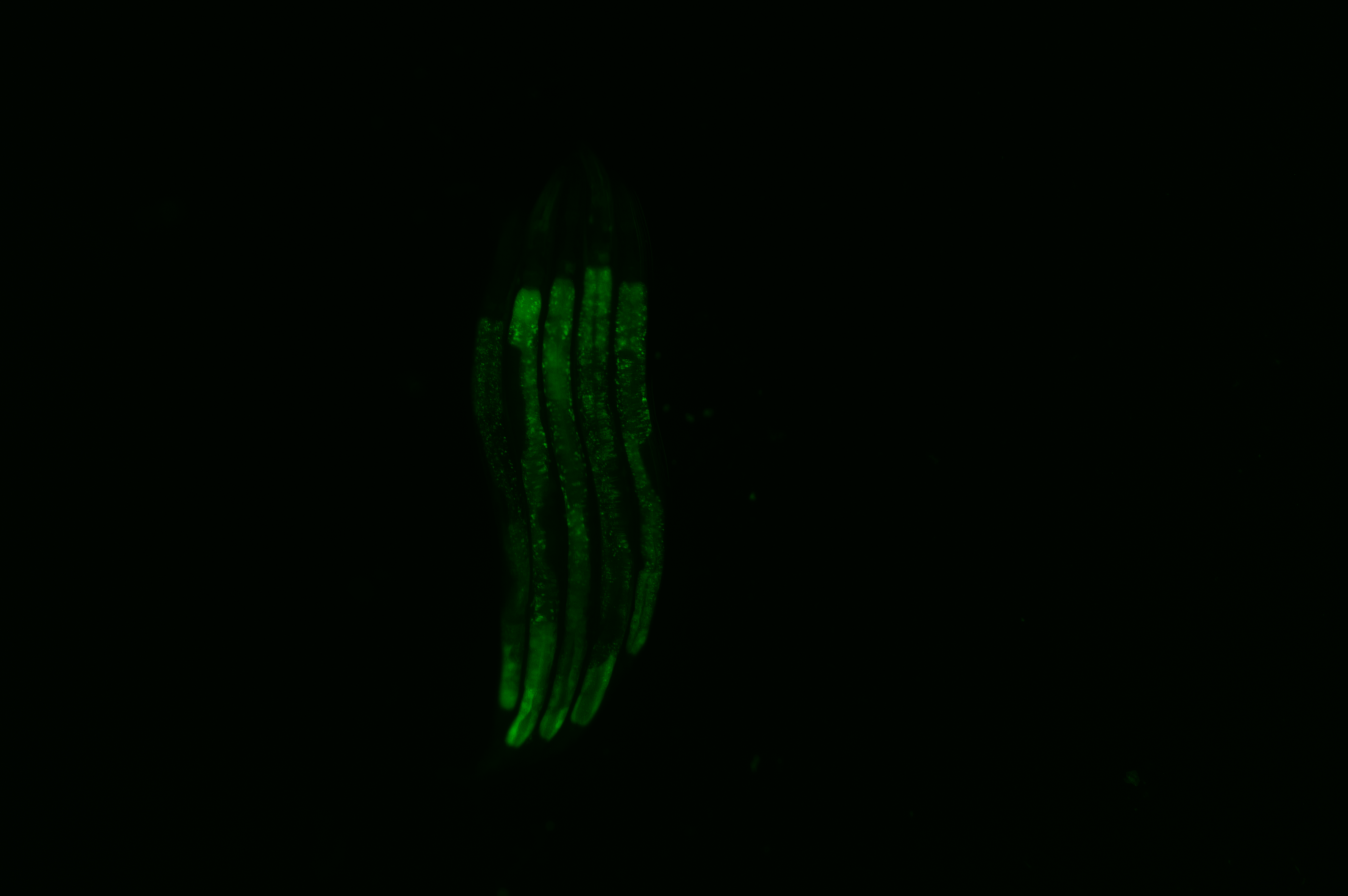

Supplement: Supplementary file 12 — Source data Fig. 8 [file 44319_2025_368_MOESM12_ESM.zip › Fig. 8 source data/8C/daf-18(ok480); unc-23(e25);tra-3 RNAi,.tif]

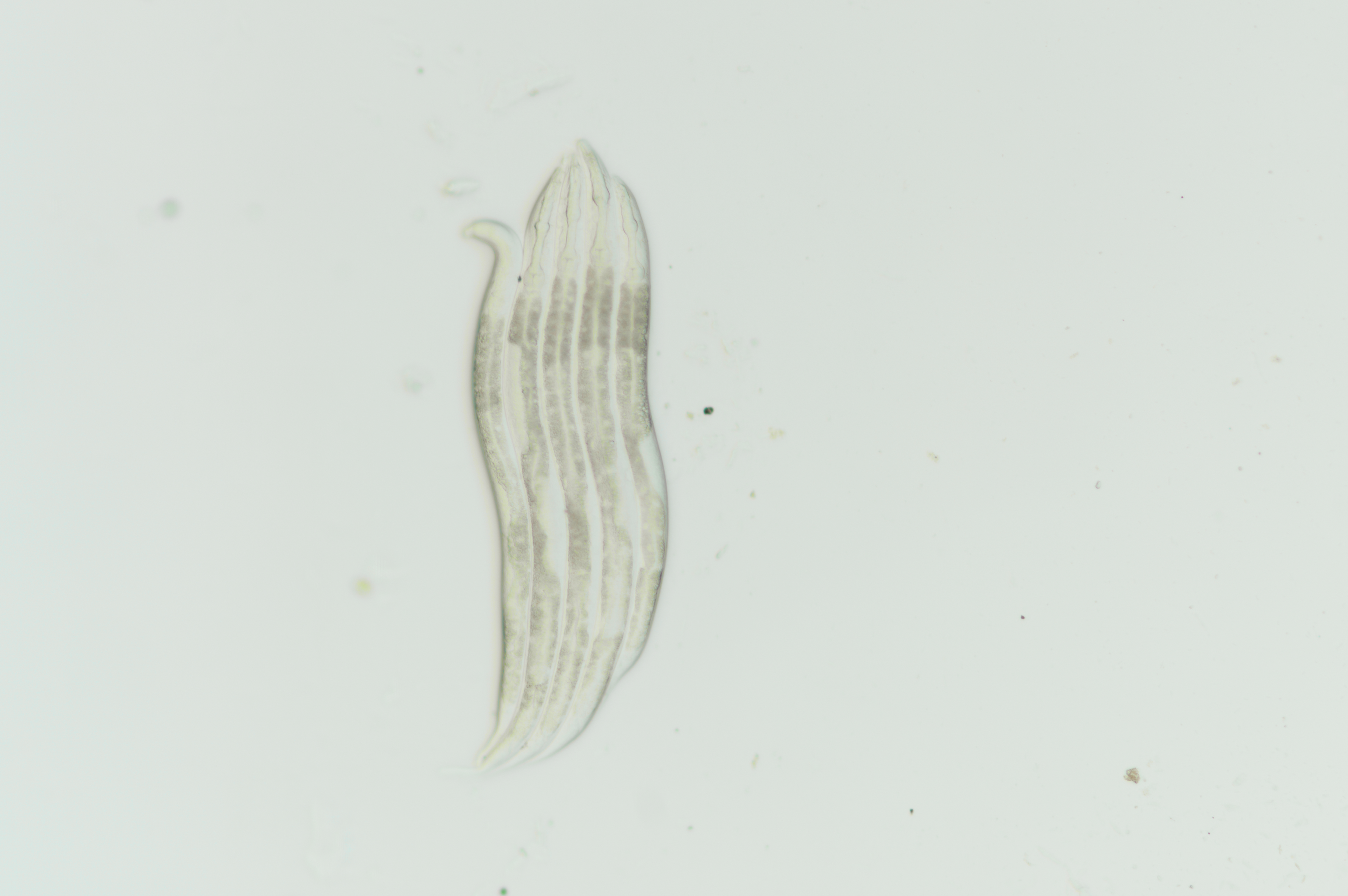

Supplement: Supplementary file 12 — Source data Fig. 8 [file 44319_2025_368_MOESM12_ESM.zip › Fig. 8 source data/8C/daf-18(ok480); unc-23(e25);tra-3 RNAi.tif]

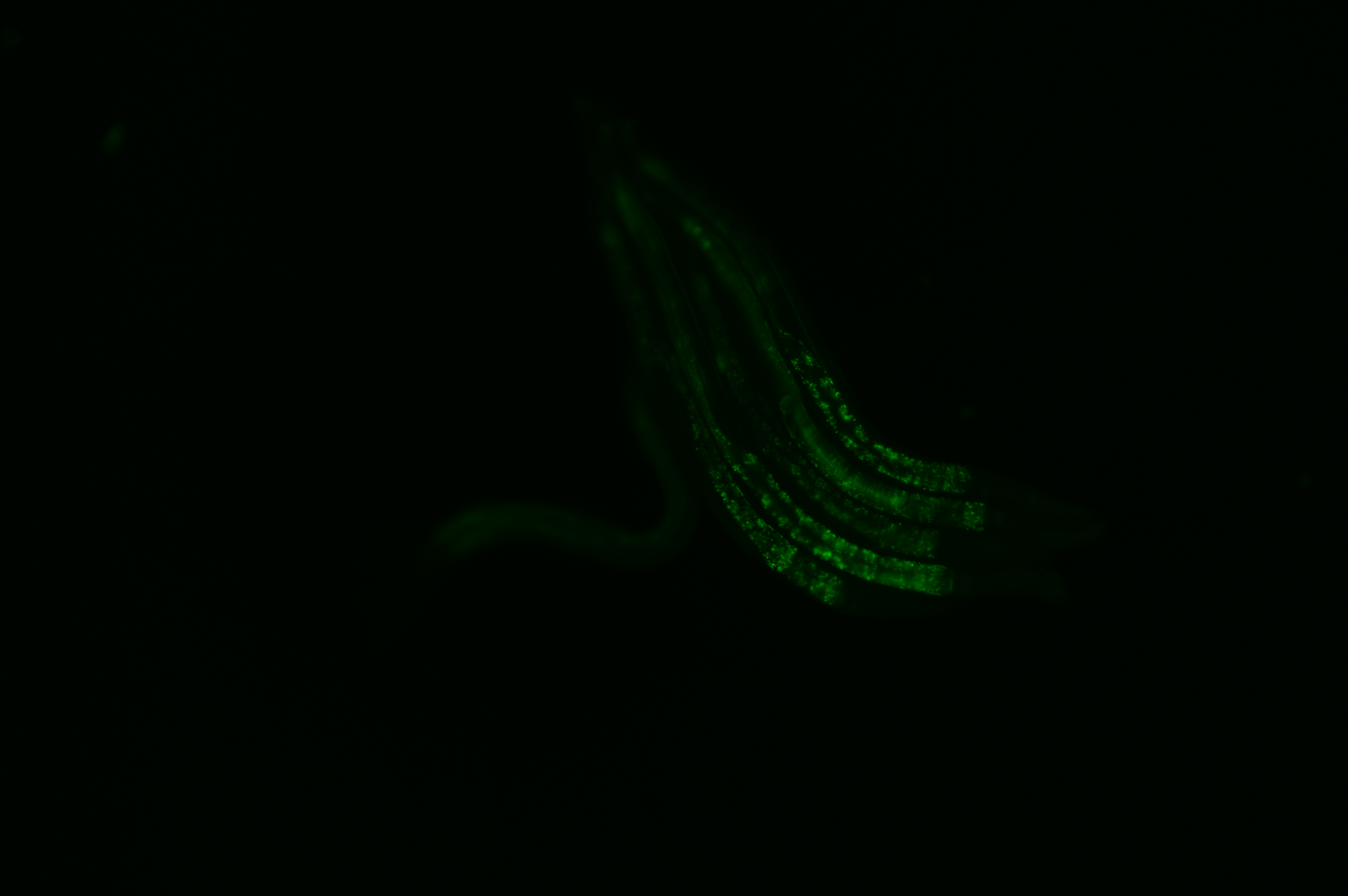

Supplement: Supplementary file 12 — Source data Fig. 8 [file 44319_2025_368_MOESM12_ESM.zip › Fig. 8 source data/8E/daf-18(ok480);fem-2(b245); control,.tif]

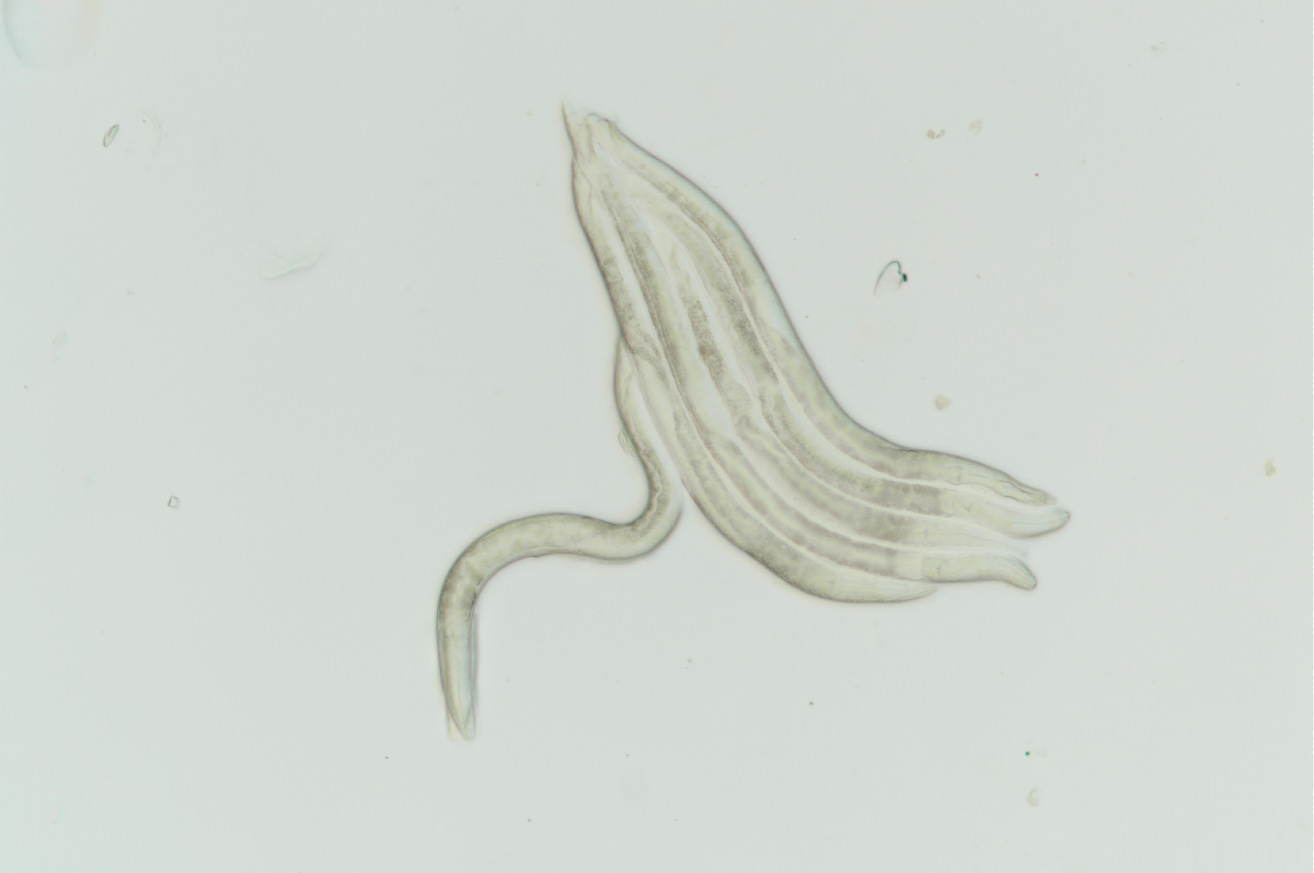

Supplement: Supplementary file 12 — Source data Fig. 8 [file 44319_2025_368_MOESM12_ESM.zip › Fig. 8 source data/8E/daf-18(ok480);fem-2(b245); control.tif]

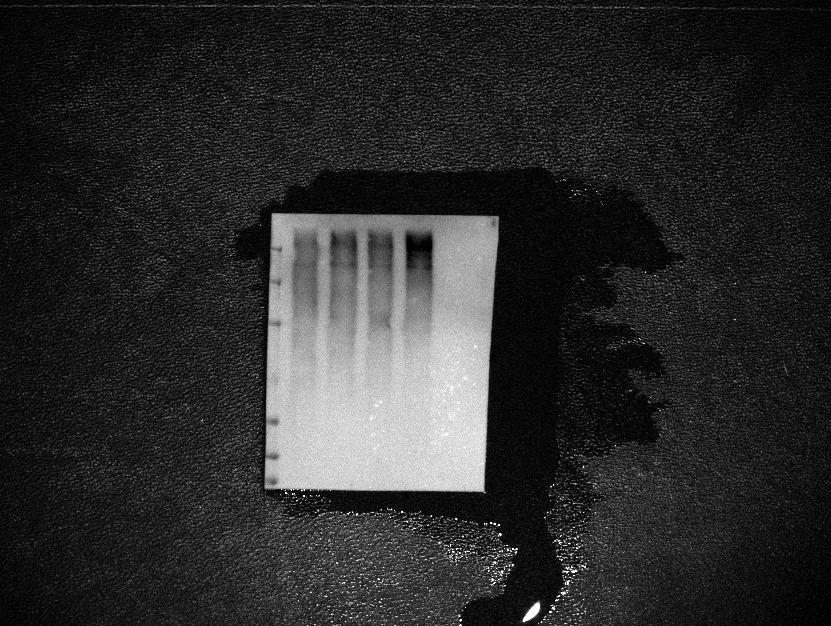

Supplement: Supplementary file 12 — Source data Fig. 8 [file 44319_2025_368_MOESM12_ESM.zip › Fig. 8 source data/8H/Fig.8H-k48 raw.png]

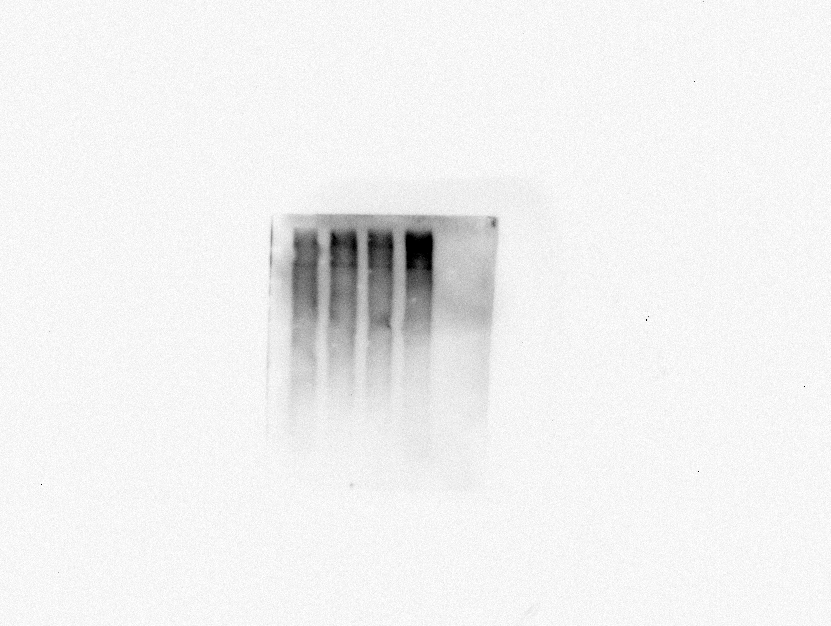

Supplement: Supplementary file 12 — Source data Fig. 8 [file 44319_2025_368_MOESM12_ESM.zip › Fig. 8 source data/8H/Fig.8H-k48.png]

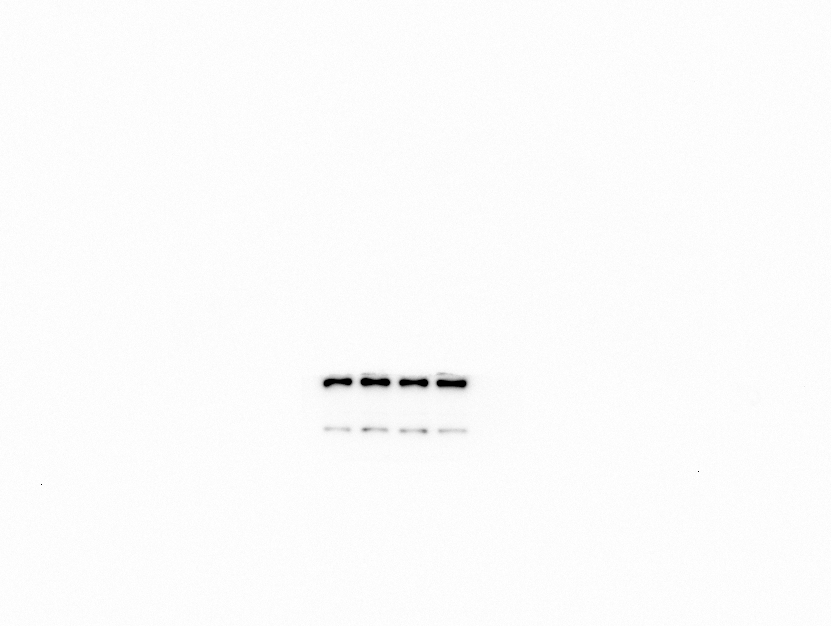

Supplement: Supplementary file 12 — Source data Fig. 8 [file 44319_2025_368_MOESM12_ESM.zip › Fig. 8 source data/8H/Fig.8H-tubulin.png]

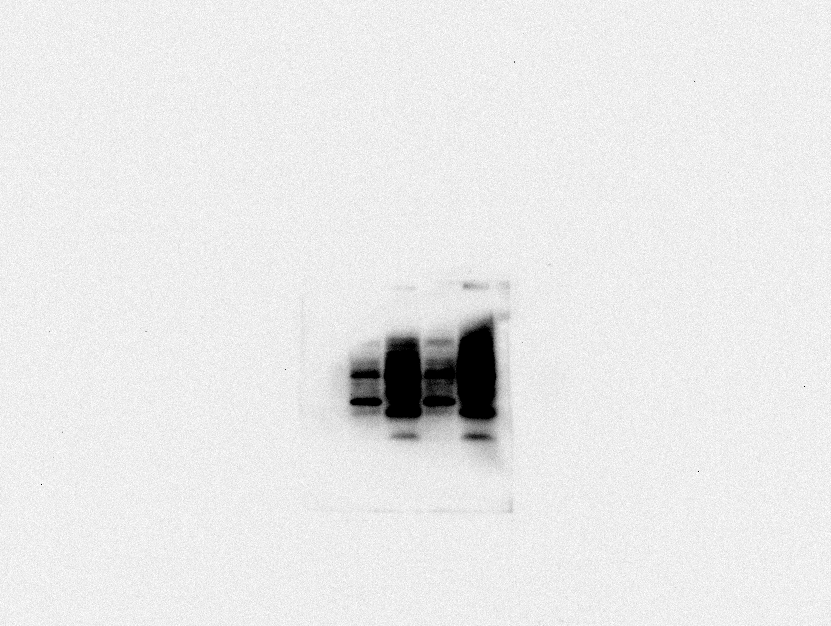

Supplement: Supplementary file 13 — Source data Fig. 9 [file 44319_2025_368_MOESM13_ESM.zip › Fig. 9 source data/9F/Fig.9F-A beta.png]

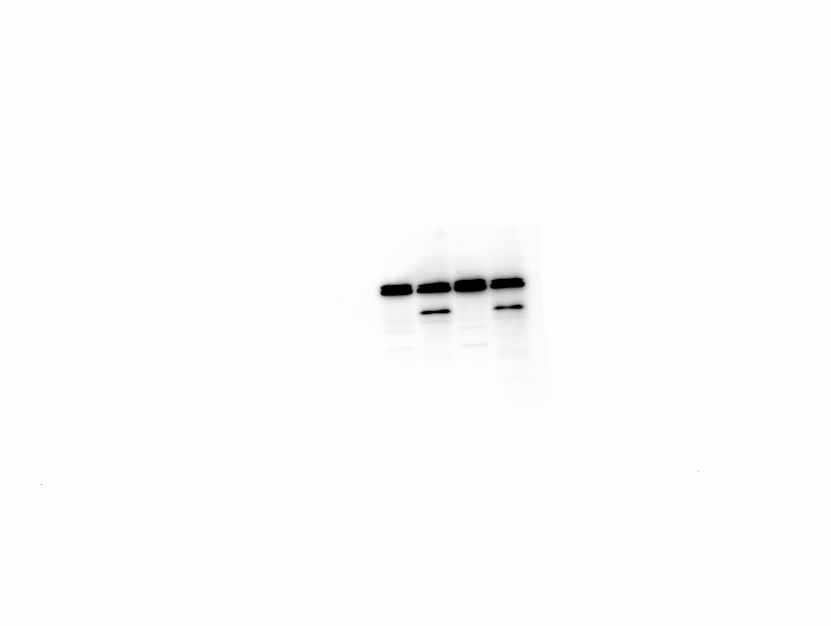

Supplement: Supplementary file 13 — Source data Fig. 9 [file 44319_2025_368_MOESM13_ESM.zip › Fig. 9 source data/9F/Fig.9F-tubulin.png]

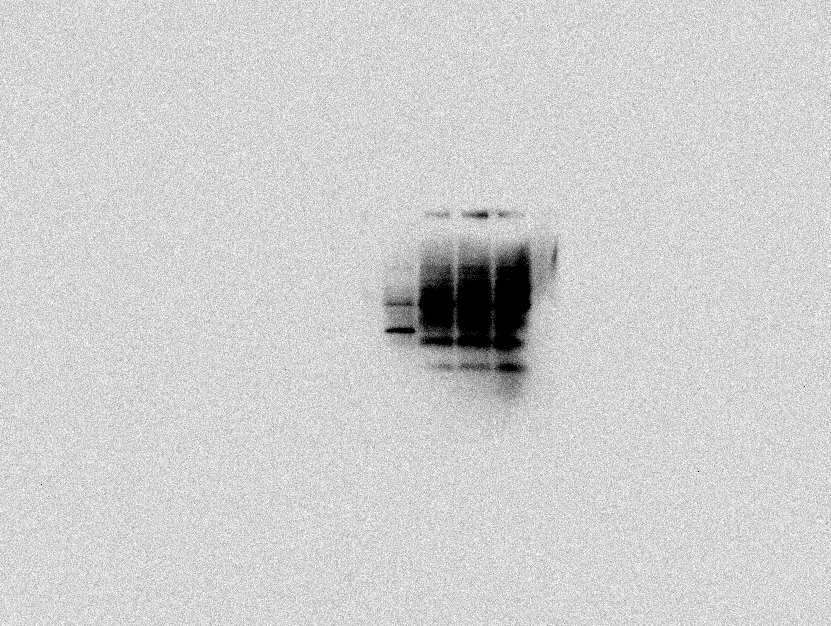

Supplement: Supplementary file 13 — Source data Fig. 9 [file 44319_2025_368_MOESM13_ESM.zip › Fig. 9 source data/9G/Fig.9G-A beta.png]

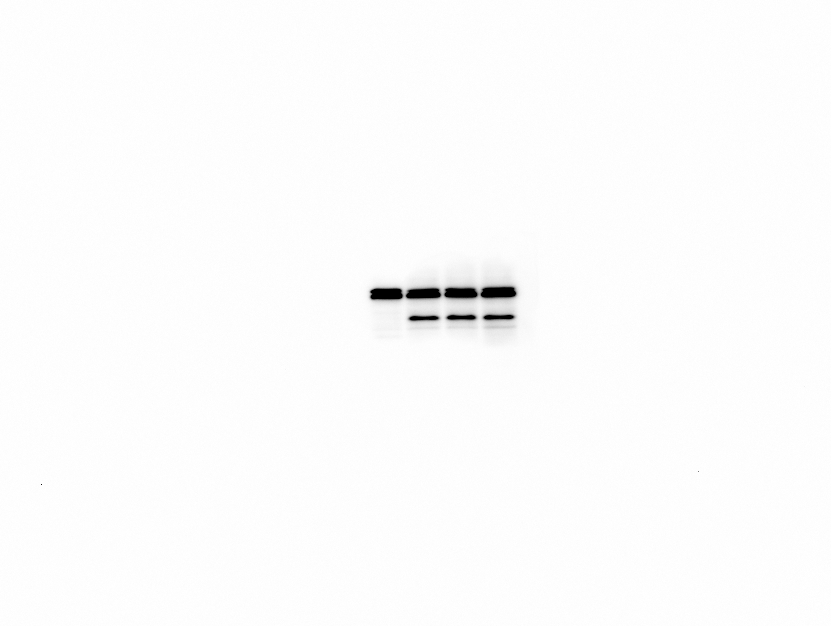

Supplement: Supplementary file 13 — Source data Fig. 9 [file 44319_2025_368_MOESM13_ESM.zip › Fig. 9 source data/9G/Fig.9G-tubulin.png]

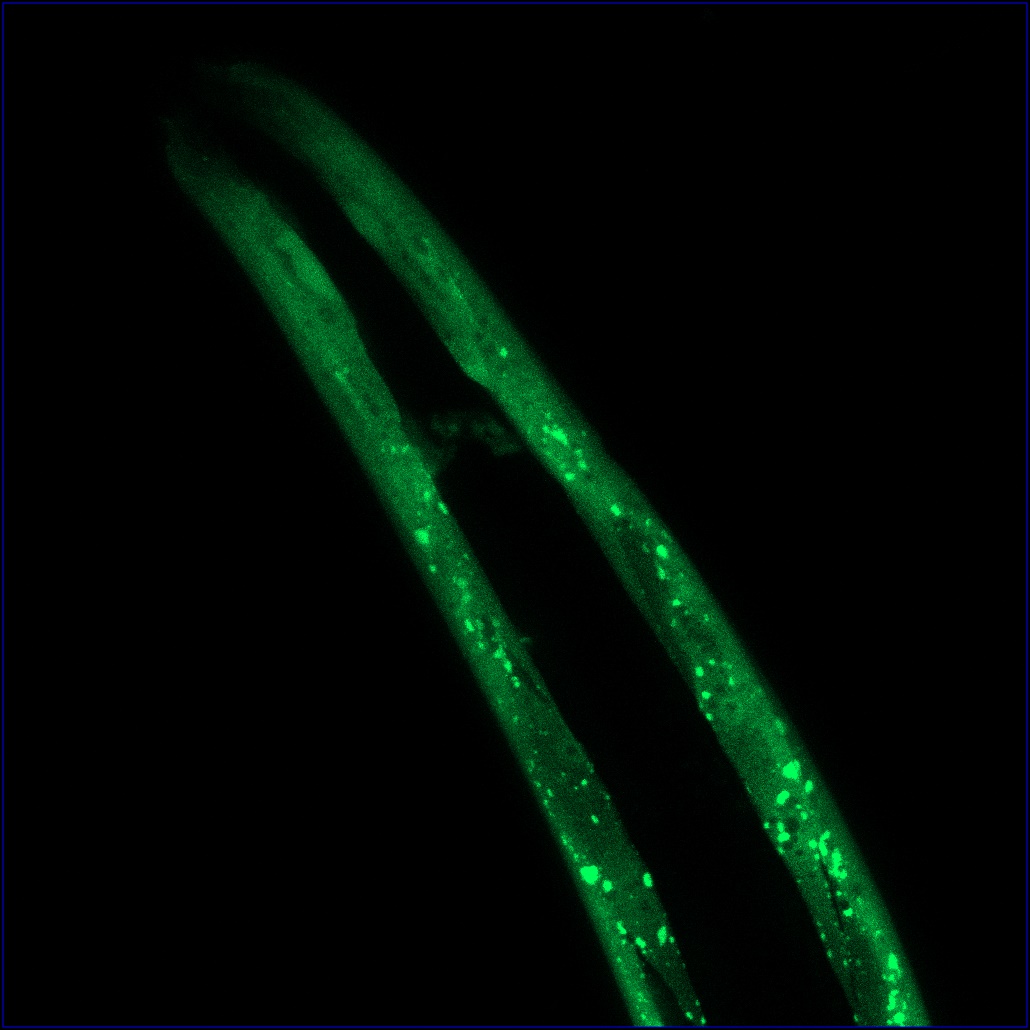

Supplement: Supplementary file 13 — Source data Fig. 9 [file 44319_2025_368_MOESM13_ESM.zip › Fig. 9 source data/9H/Control+50 uM MG132.jpg]

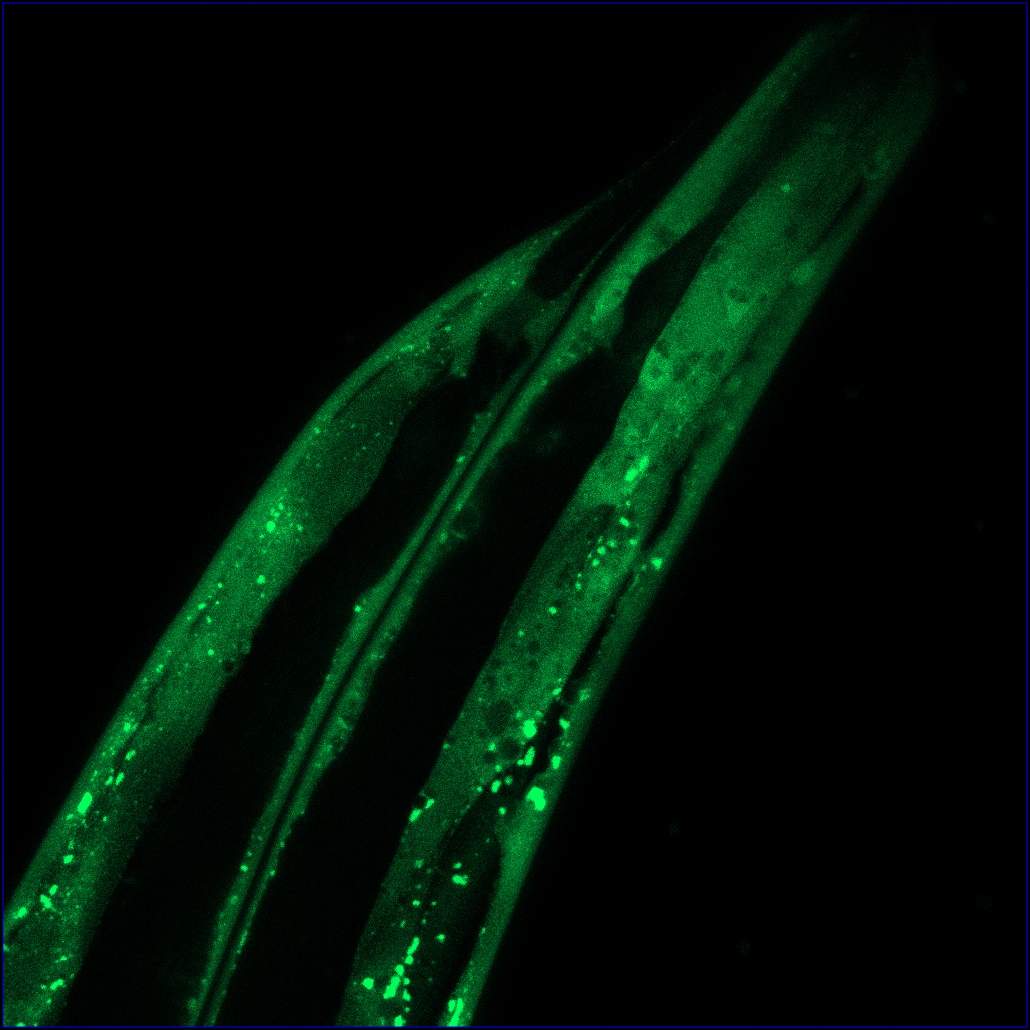

Supplement: Supplementary file 13 — Source data Fig. 9 [file 44319_2025_368_MOESM13_ESM.zip › Fig. 9 source data/9H/control.jpg]

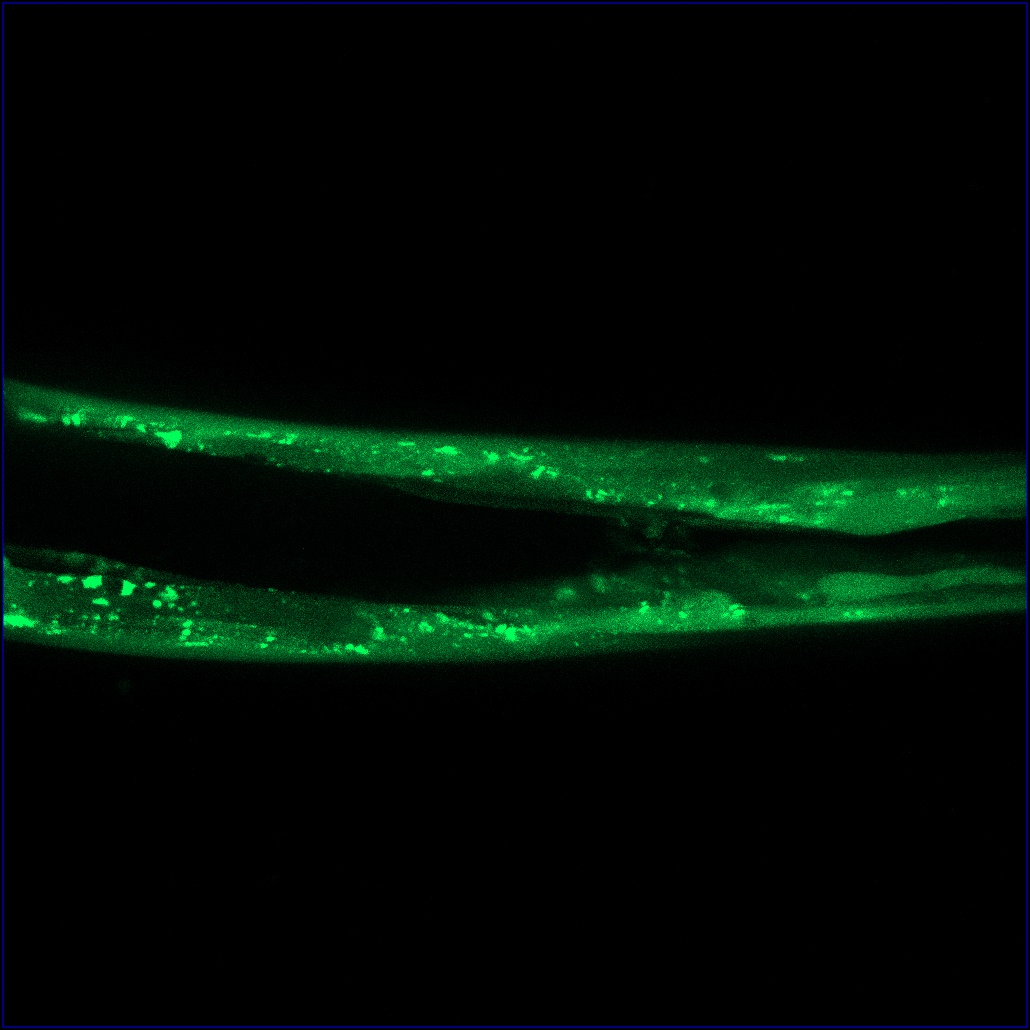

Supplement: Supplementary file 13 — Source data Fig. 9 [file 44319_2025_368_MOESM13_ESM.zip › Fig. 9 source data/9H/tra-3 RNAi+50 uM MG132.jpg]

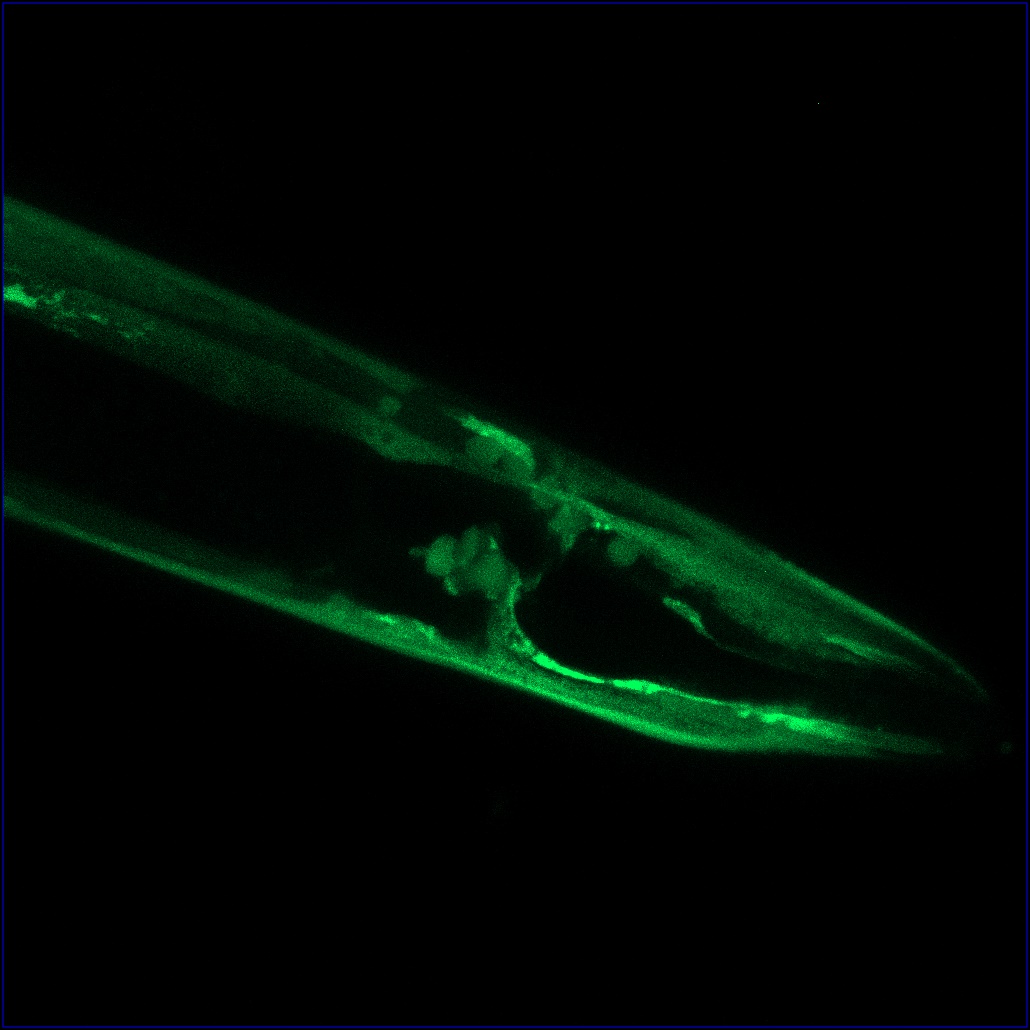

Supplement: Supplementary file 13 — Source data Fig. 9 [file 44319_2025_368_MOESM13_ESM.zip › Fig. 9 source data/9H/tra-3 RNAi.jpg]

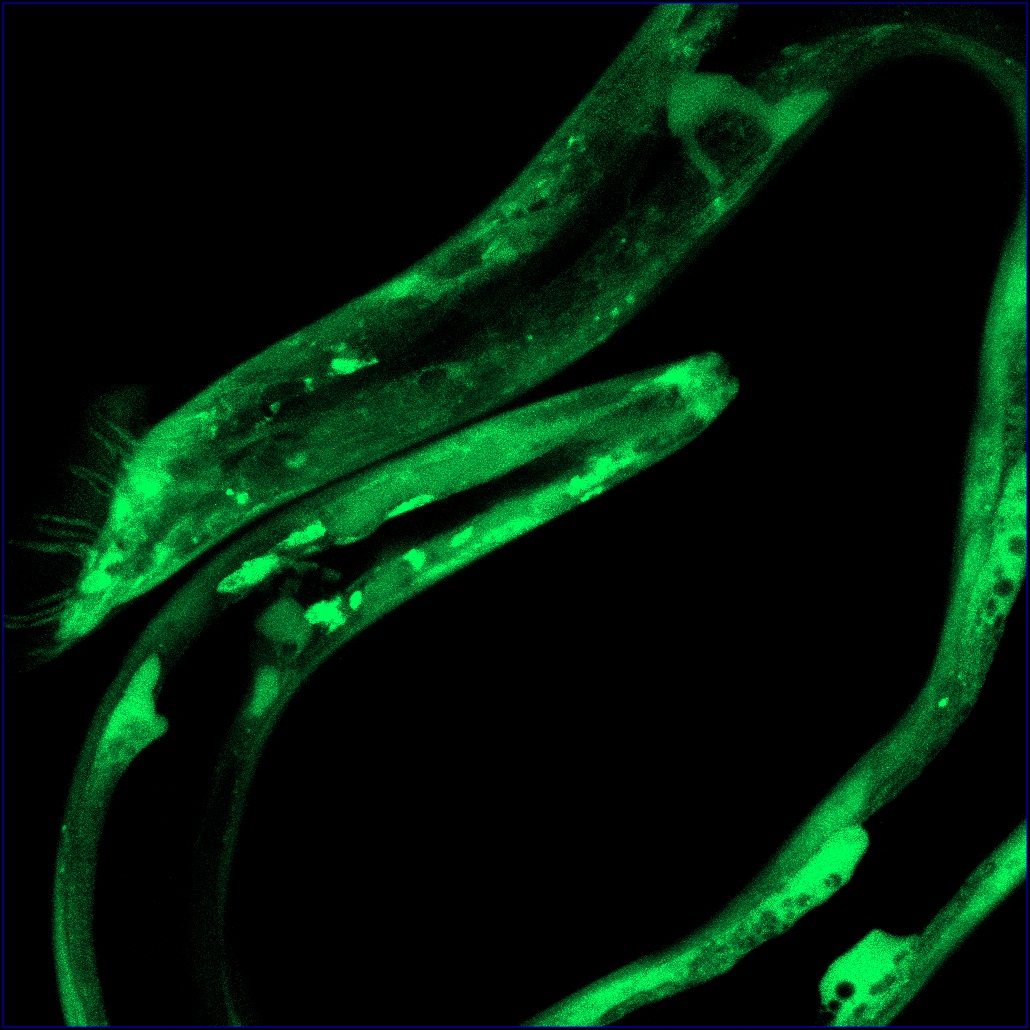

Supplement: Supplementary file 13 — Source data Fig. 9 [file 44319_2025_368_MOESM13_ESM.zip › Fig. 9 source data/9I/Control +50uM MG132.jpg]

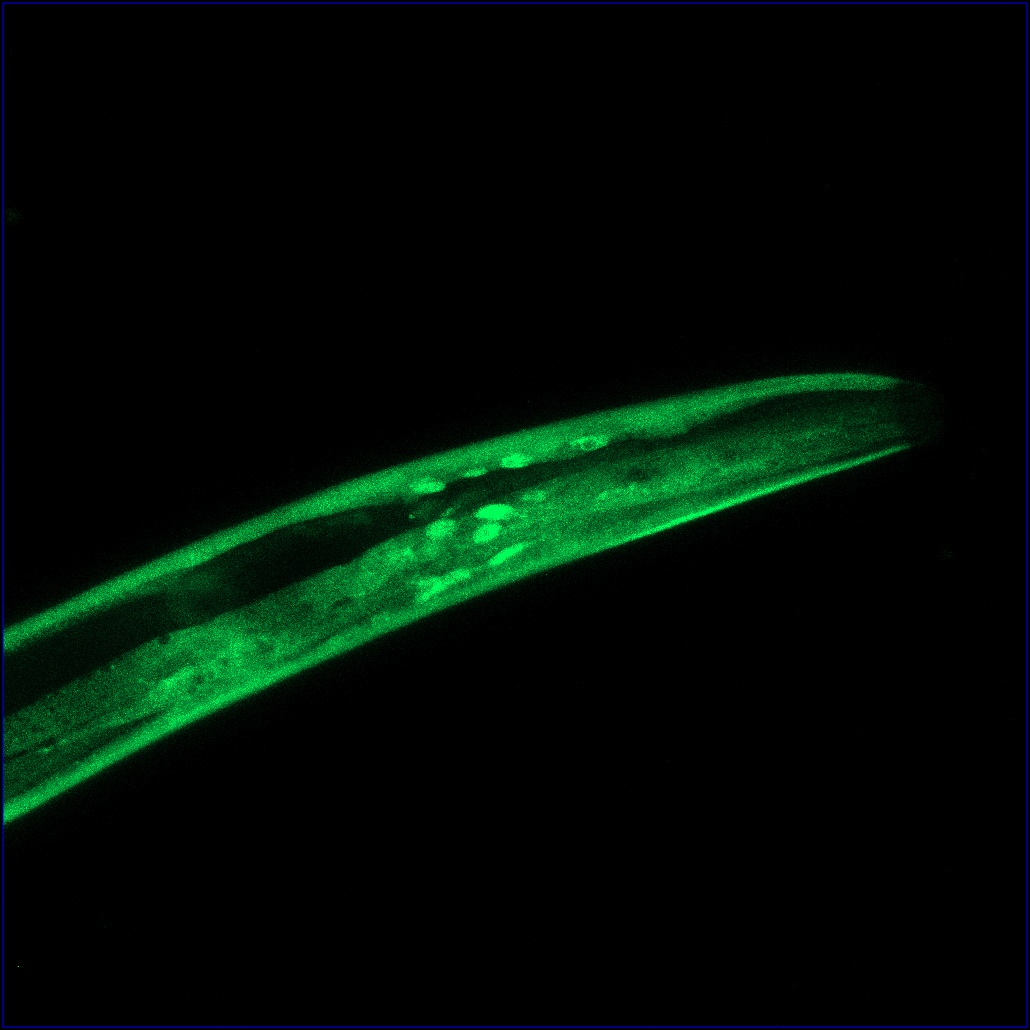

Supplement: Supplementary file 13 — Source data Fig. 9 [file 44319_2025_368_MOESM13_ESM.zip › Fig. 9 source data/9I/control+50uM MG132.jpg]

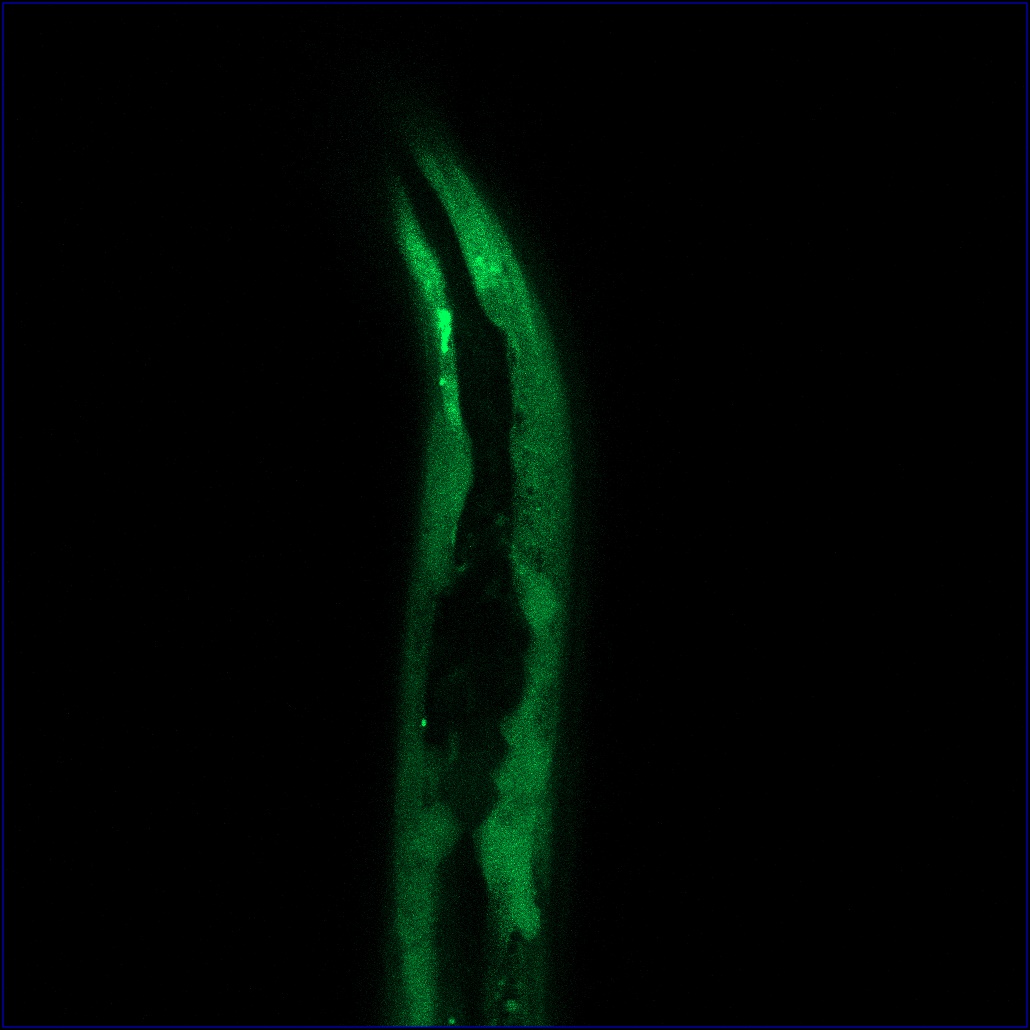

Supplement: Supplementary file 13 — Source data Fig. 9 [file 44319_2025_368_MOESM13_ESM.zip › Fig. 9 source data/9I/Control.jpg]

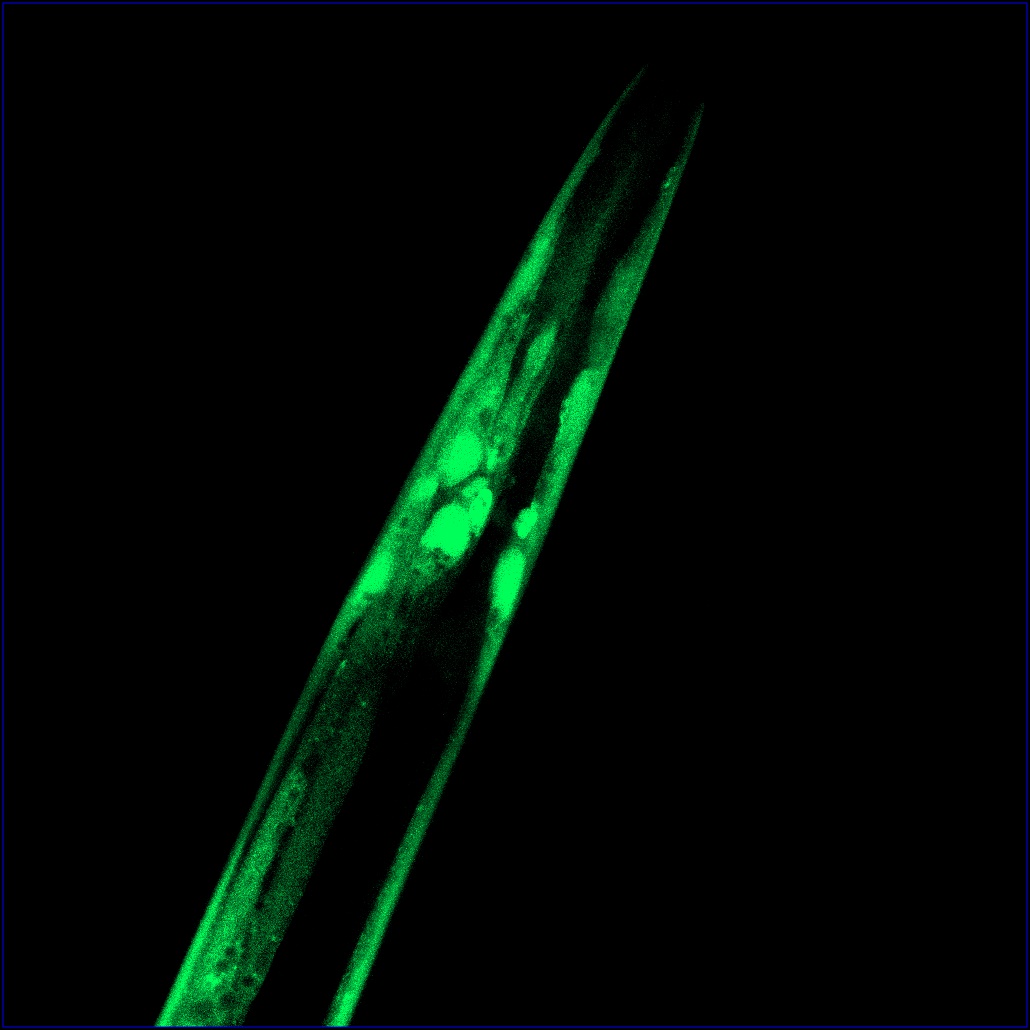

Supplement: Supplementary file 13 — Source data Fig. 9 [file 44319_2025_368_MOESM13_ESM.zip › Fig. 9 source data/9I/fem-2 RNAi +50uM MG132.jpg]
