# Supplementary material for: Male sex determination maintains proteostasis and extends lifespan of daf-18/PTEN deficient C. elegans
Source: EMBO Rep. 2025 Jan 16;26(4):1084–113. doi: 10.1038/s44319-025-00368-x (PMC11850635; doi:10.1038/s44319-025-00368-x)
Supplement: Supplementary file 14 — Expanded View Figures [file 44319_2025_368_MOESM14_ESM.pdf]

Expanded View Figures

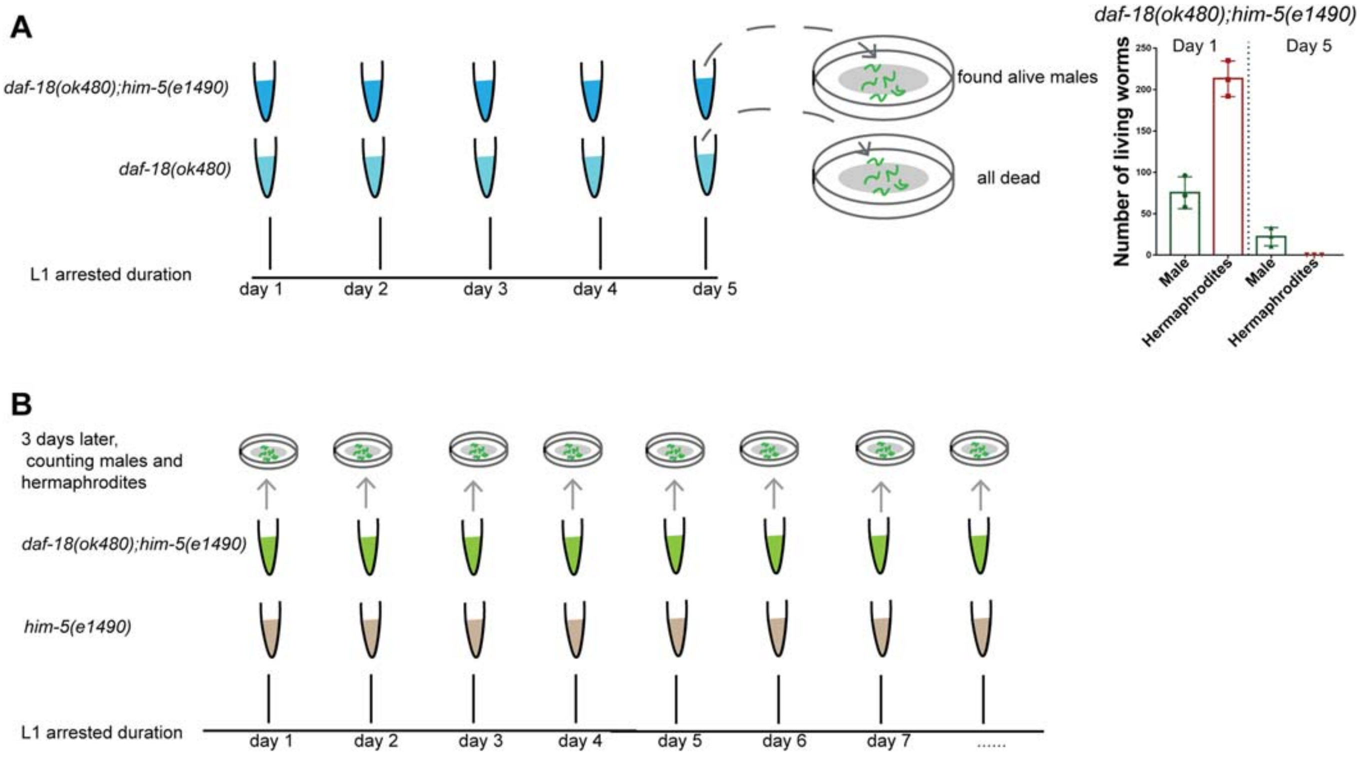

Figure EV1. ***daf-18(ok480)*** **males live longer than hermaphrodites in L1 arrest stage.**

(**A**) L1 arrested *daf-18(ok480)* hermaphrodites normally live about 4 days in M9, males were found alive after day 4. The surviving L1 arrested worms were recovered into NGM plates seeded with OP50, and checked after 3 days culturing at 20°C. 100 µL liquid with L1 arrested worms were recovered each repeat. (**B**) *him-5(e1490)* and *daf-18(ok480);him-5(e1490)* worms were maintained in M9 added with 0.08% (v/v) ethanol, transferred 50-100 µL liquid with L1 arrested worms (more than 100) every day into NGM plates seeded with OP50, and checked 3 days after culturing at 20 °C. The percentages of males and hermaphrodites were calculated every day. The experiment was repeated three times independently. The data show the average of three independent repeats, and the error bars show the standard deviations.

© The Author(s)

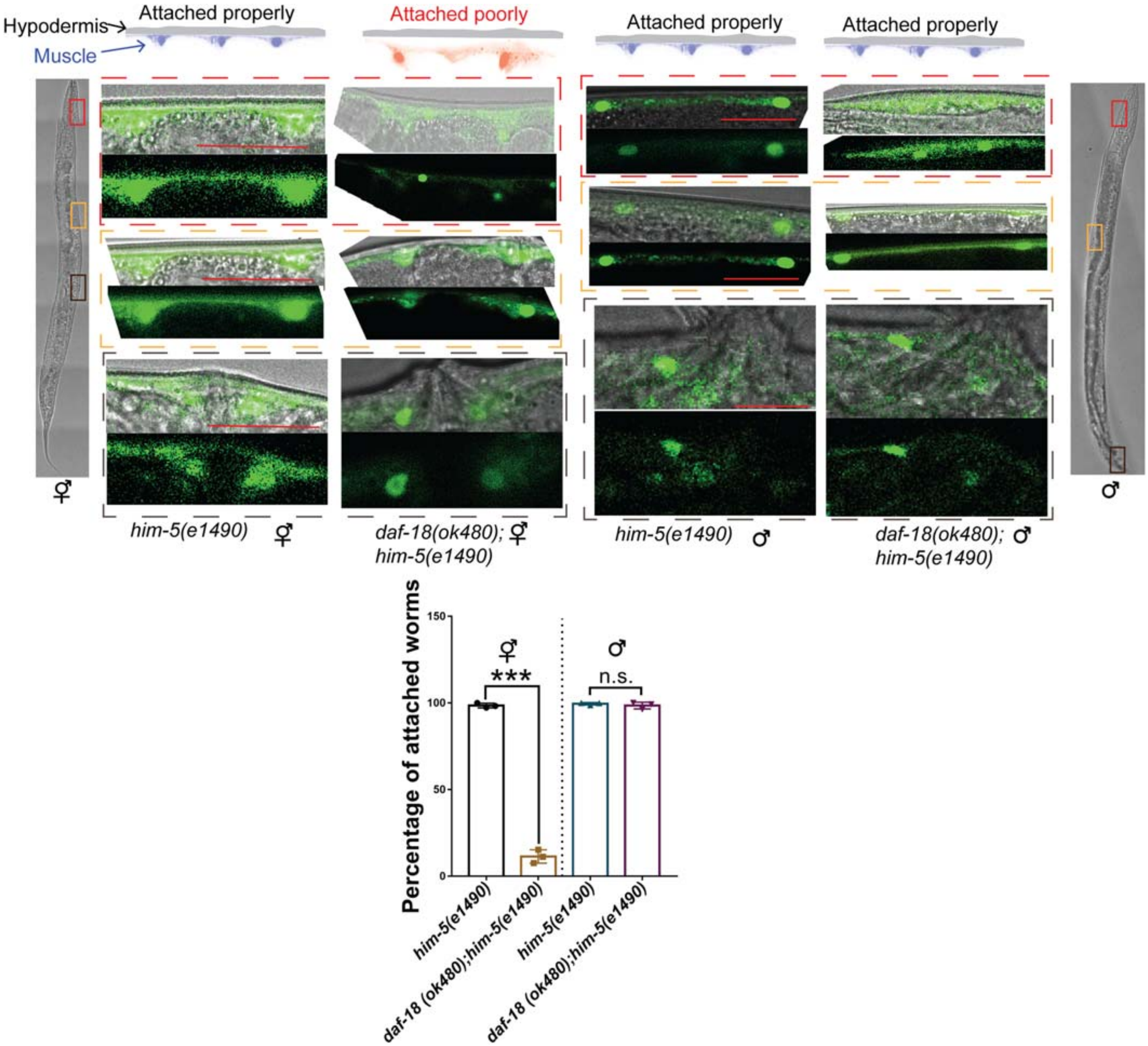

Figure EV2. ***daf-18(ok480)*** **hermaphrodites reduce the muscle attachment with hypodermis.**

him-5(e1490) and *daf-18(ok480);him-5(e1490)* hermaphrodites were checked for muscle attachment with hypodermis at head, vulva and worm body. The status of attachment at all these three points was counted as "Attached properly". *him-5(e1490)* and *daf-18(ok480);him-5(e1490)* males were checked for muscle attachment with hypodermis at head, tail and worm body. The status of males at all these three points was counted as "Attached properly". The experiment was repeated three times independently, with sample size (n) = 60 for each mutant. The data show the average of three independent repeats, and the error bars show the standard deviations. P value was determined by using two-tailed *t-test*. ***P = 3.11987E-06, n.s.: no significant difference=0.42777. Red scale bar: 50 μm. As *unc-23* is responsible for the proper attachment of hypodermis and muscles, the downregulated *unc-23* in *daf-18* hermaphrodites resulted in detachment. However, males had no significant change, which also suggest males may up-regulate *unc-23* expression to defense against the loss of *daf-18*.

 © The Author(s)

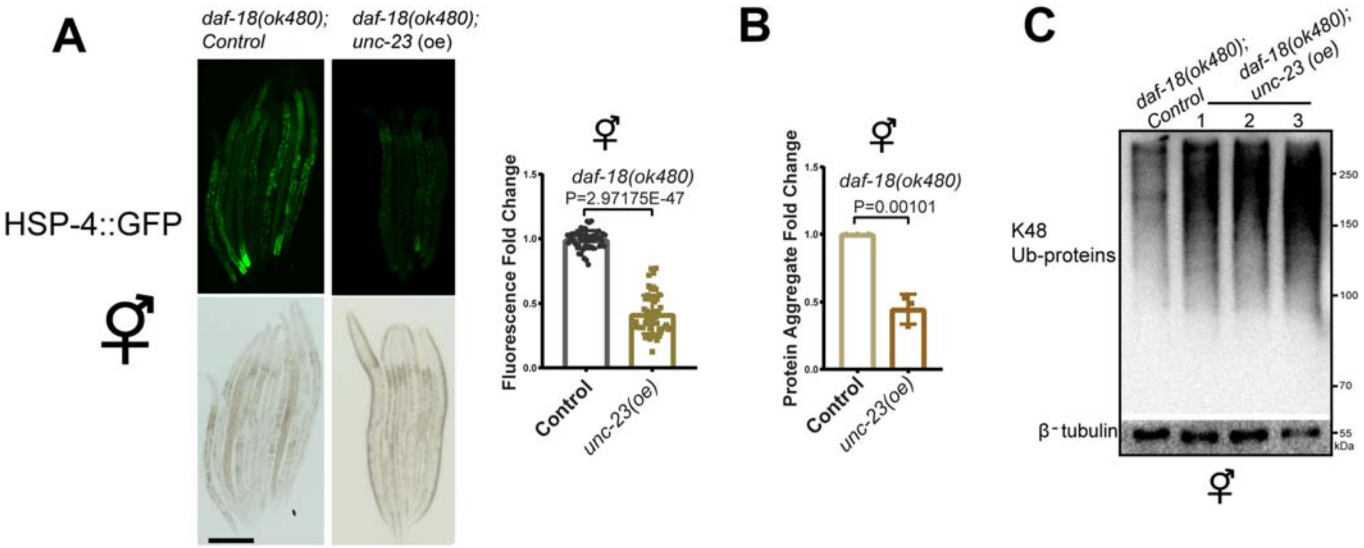

Figure EV3. *unc-23* overexpression in *daf-18(ok480)* hermaphrodites rescued the protein homeostasis.

(**A**) The unfold protein marker *hsp-4p*::GFP in hermaphrodites. Each experiment set has three independent repeats, sample size (n)=54. Control: the transgenic injection strains with empty expression vector L2528. The data shows the values of all samples from three replicates, with error bars representing the averages and standard deviations. *P* values were determined by using two-tailed *t-test* (*P* value: 2.97175E-47). Scale bar: 200 μm. (**B**) The total level of protein aggregation in hermaphrodites analyzed by using PROTEOSTAT Protein Aggregation Assay. The fold changes of protein aggregation in hermaphrodites of three independent experiments. Control: the transgenic injection strains with empty expression vector L2528. The data show the average of three independent repeats, and the error bars show the standard deviations. *P* value was determined by using two-tailed *t-test* (*P* value: 0.00101). (**C**) The k48 linked protein ubiquitination of hermaphrodites. Control: the transgenic injection strains with empty expression vector L2528. Each immunoblot shows representative of three times independent experiments, all the data details summarized in Appendix Table S5.

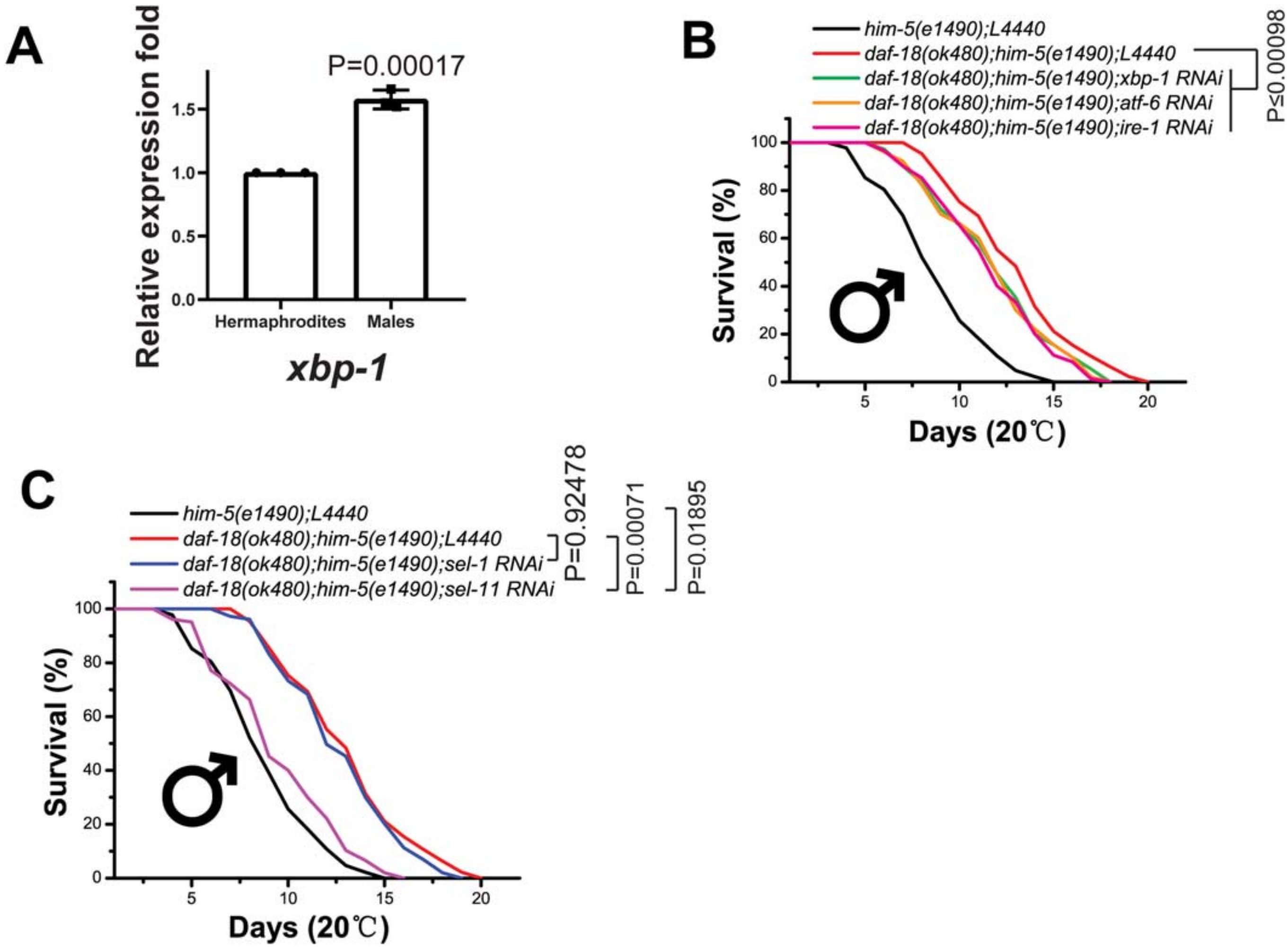

Figure EV4. The HRD1/SEL-11 and XBP-1/ERAD may be involved in regulating the male survival advantage when *daf-18* is lost.

(**A**) The expression of *xbp-1* in *daf-18(ok480)* males is higher than that in hermaphrodites. The data show the average of three independent repeats, and the error bars show the standard deviations. *P* value was determined by using two-tailed *t-test* (*P* value: 0.00017). (**B**) Knocking down *xbp-1 (n = 65)*, *atf-6 (n = 102)* and *ire-1 (n = 69)* can reduce the adult lifespan of *daf-18(ok480)* males. (**C**) Knocking down *sel-11 (n = 97)*, not *sel-1 (n = 95)*, significantly change the adult lifespan of *daf-18(ok480)* males. Control: RNAi control clones containing the empty vector L4440. Each lifespan experiment set was repeated three times. The mean survival rates were calculated using the Kaplan–Meier method, and *P* values were determined by using the log rank test (*P* values; **B**, *daf-18(ok480);him-5(e1490);*L4440 males vs *xbp-1 RNAi* males: 0.00027; *daf-18(ok480);him-5(e1490);*L4440 males vs *atf-6 RNAi* males: 0.00089; *daf-18(ok480);him-5(e1490);*L4440 males vs *ire-1 RNAi* males: 0.00075; **C**, *daf-18(ok480);him-5(e1490);*L4440 males vs *sel-1 RNAi* males: 0.92478; *daf-18(ok480);him-5(e1490);*L4440 males vs *sel-11 RNAi* males: 0.00071; *him-5(e1490);*L4440 males vs *daf-18(ok480);him-5(e1490);sel-11 RNAi* males: 0.01895). All the lifespan data are summarized in Appendix Table S4.

© The Author(s)

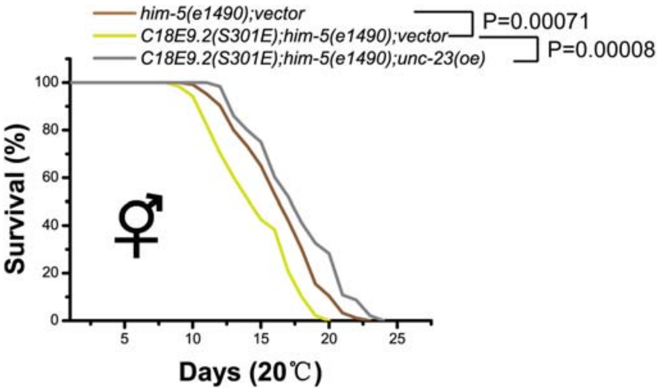

Figure EV5. Overexpression of *unc-23* can extend the adult lifespan of *C18E9.2(S301E)* hermaphrodites.

The lifespan of *C18E9.2(S301E)* can be affected by overexpression of *unc-23*. Sample size (n) = 68 (*him-5 vector*), 74 (*C18E9.2 vector*), 63 (*unc-23 oe*). Each lifespan experiment set was repeated three times. The mean survival rates were calculated using the Kaplan–Meier method, and *P* values were determined by using the log rank test (*P* values; *him-5(e1490);vector* hermaphrodites *vs* *C18E9.2(S301E);him-5(e1490);vector* hermaphrodites: 0.00071; *C18E9.2(S301E);-him-5(e1490);vector* hermaphrodites vs *C18E9.2(S301E);him-5(e1490);unc-23(oe)* hermaphrodites: 0.00008). All the lifespan data are summarized in Appendix Table S4.

© The Author(s)

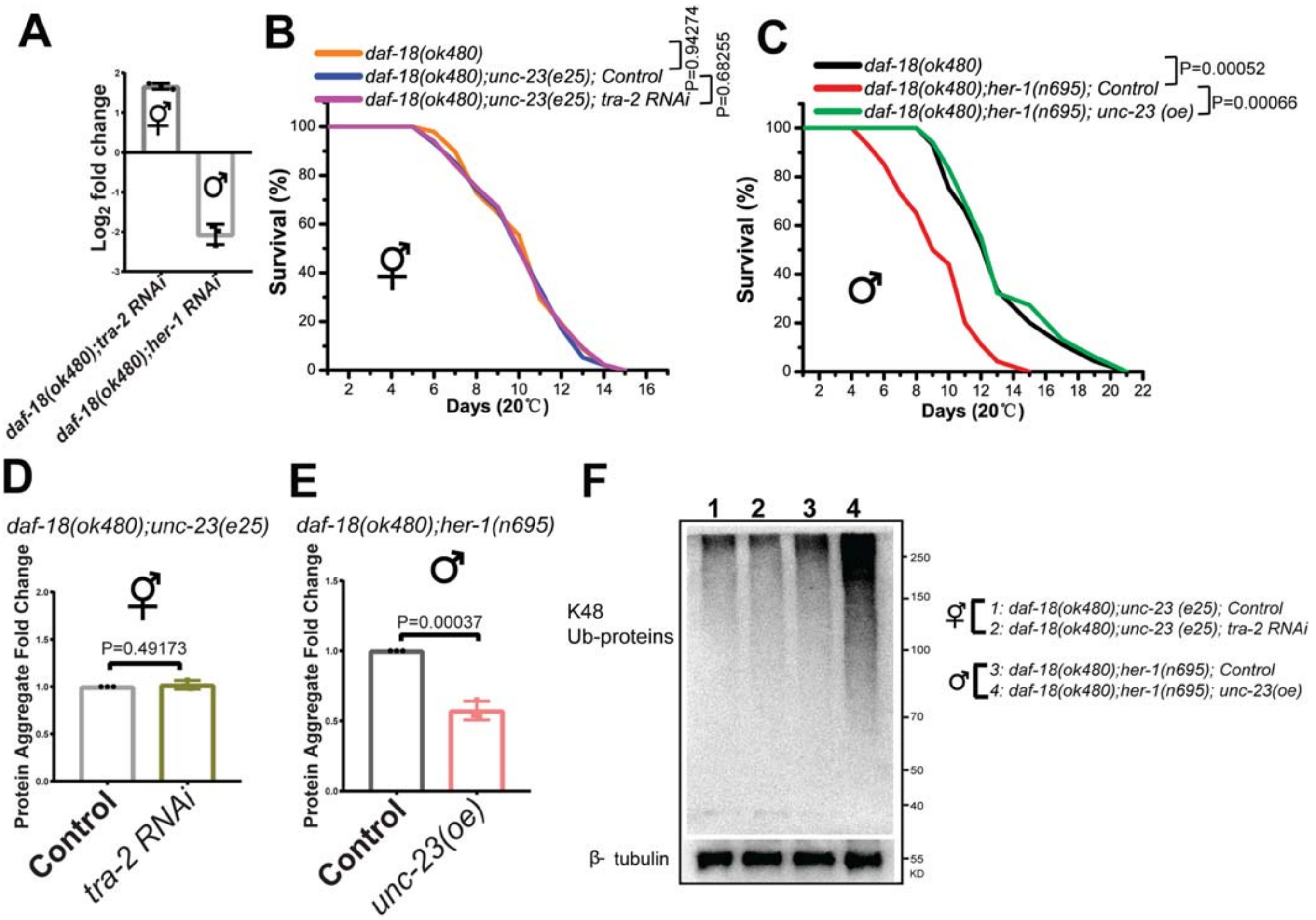

Figure EV6. ***unc-23* may be regulated by male sex determination pathway.**

(**A**) Real-time PCR tested the expression changes of *unc-23*. All these genes were significantly up- or downregulated by at least 2-fold. Log2FC: log2 (fold change) (> 1 or < −1). The data show the average of three independent repeats, and the error bars show the standard deviations. (**B**) Enhancing male sex determination signaling by knocking down *tra-2* failed to extend the lifespan *daf-18(ok480);unc-23(e25)* hermaphrodites. Control: Feeding RNAi control clones with empty vector L4440. Sample size (n) = 96 (*daf-18*), 156 (*control*), 88 (*tra-2*). (**C**) Overexpressed *unc-23* in *daf-18(ok480); her-1(n695)* rescued the shorted lifespan. Control: the transgenic injection strains with empty expression vector L2528. Sample size (n) = 94 (*daf-18*), 72 (*control*), 63 (*unc-23 oe*). Each lifespan experiment set was repeated three times. The mean survival rates were calculated using the Kaplan–Meier method, and P values were determined by using the log rank test (P values; **B**, *daf-18(ok480)* hermaphrodites vs *daf-18(ok480);unc-23(e25)* hermaphrodites: 0.94274; *daf-18(ok480);unc-23(e25)* hermaphrodites vs *daf-18(ok480);unc-23(e25);tra-2 RNAi* hermaphrodites: 0.68255; **C**, *daf-18(ok480)* males vs *daf-18(ok480);her-1(n695)* males: 0.00052; *daf-18(ok480);her-1(n695)* males vs *daf-18(ok480);her-1(n695);unc-23(oe)* males: 0.00066). (**D**) The fold changes of protein aggregation in *daf-18(ok480);unc-23(e25)* hermaphrodites of three independent experiments when knocking down *tra-2*. Control: Feeding RNAi control clones with empty vector L4440. (**E**) The fold changes of protein aggregation in *daf-18 (ok480);her-1(n695)* males of three independent experiments when overexpressing *unc-23*. Control: the transgenic injection strains with empty expression vector L2528. The data show the average of three independent repeats, and the error bars show the standard deviations. P value was determined by using two-tailed *t-test* (P values; **D**, *control* hermaphrodites vs *tra-2 RNAi* hermaphrodites: 0.49173; **E**, *control* males *vs unc-23(oe)* males: 0.00037). (**F**) The k48 linked protein ubiquitination. Control for RNAi: Feeding RNAi control clones with empty vector L4440. Control for overexpression: the transgenic injection strains with empty expression vector L2528. All the lifespan data were summarized in Appendix Table S4. Each immunoblot shows representative of three times independent experiments, all the data details summarized in Appendix Table S5.

© The Author(s)
